# Synthesis and Evaluation of Novel Iminosugars Prepared from Natural Amino Acids

**DOI:** 10.3390/molecules26020394

**Published:** 2021-01-13

**Authors:** Alejandro Puet, Gema Domínguez, Francisco Javier Cañada, Javier Pérez-Castells

**Affiliations:** 1 Department of Chemistry and Biochemistry, Facultad de Farmacia, Universidad San Pablo-CEU, CEU Universities, Urbanización Montepríncipe, Boadilla del Monte, 28660 Madrid, Spain; ale.puet.ce@ceindo.ceu.es (A.P.); gdommar@ceu.es (G.D.); 2 Centro de Investigaciones Biológicas Margarita Salas, CSIC, Ramiro de Maetzu 9, 28040 Madrid, Spain; jcanada@cib.csic.es; 3 CIBER de Enfermedades Respiratorias (CIBERES) Avda, Monforte de Lemos 3-5, 28029 Madrid, Spain

**Keywords:** piperidine iminosugars, glycosidase inhibition, metathesis, sulfur ylide cyclopropanation

## Abstract

Cyclopropanated iminosugars have a locked conformation that may enhance the inhibitory activity and selectivity against different glycosidases. We show the synthesis of new cyclopropane-containing piperidines bearing five stereogenic centers from natural amino acids l-serine and l-alanine. Those prepared from the latter amino acid may mimic l-fucose, a natural-occurring monosaccharide involved in many molecular recognition events. Final compounds prepared from l-serine bear *S* configurations on the C5 position. The synthesis involved a stereoselective cyclopropanation reaction of an α,β-unsaturated piperidone, which was prepared through a ring-closing metathesis. The final compounds were tested as possible inhibitors of different glycosidases. The results, although, in general, with low inhibition activity, showed selectivity, depending on the compound and enzyme, and in some cases, an unexpected activity enhancement was observed.

## 1. Introduction

**Figure 1:**
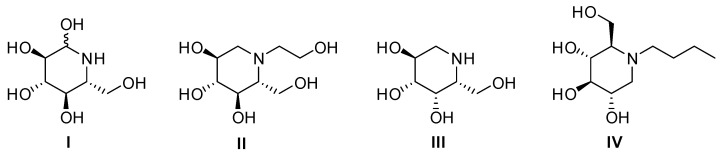
Structures of nojirimycin (**I**), miglitol (**II**), migalastat (**III**), and miglustat (**IV**).

Natural or synthetic polyhydroxylated piperidines are iminosugars able to act as biomimetics of their corresponding pyranose analogs. For instance, nojirimycin, its epimers, and their deoxyanalogs have been used as lead molecules to design glycosidase and glycosyl transferase inhibitors and modulators [1,2,3]. Some iminosugars such as miglitol (Glyset^®^) [4], migalastat (Galafold^®^) [5], and miglustat (Zavesca^®^) [6] are commercially available, and others are actually in different clinical phases (Figure 1). The interaction with glycosidases is generally attributed to a structural similarity to diverse conformational oxocarbenium transition states formed during the hydrolysis of carbohydrates [7]. There are important variations in these transition states for different glycosidases [8,9]. Thus, there is a great interest in designing conformationally restricted inhibitors in order to achieve selective inhibition and adequate metabolic stability.

**Figure 2:**
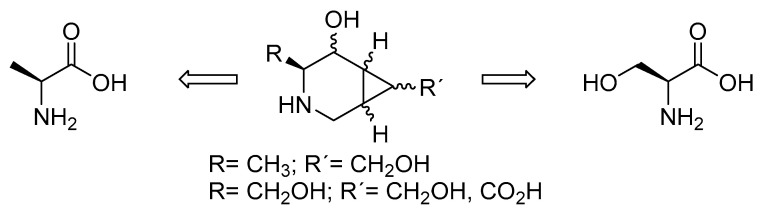
Structures of the targeted compounds.

Our group engaged in the synthesis of novel piperidine iminosugars fused to a cyclopropane ring, resulting in structures with a locked conformation [10,11]. The cyclopropane renders a twist-like conformation to the piperidine ring that is found, as preferred for interactions with certain glycosidases [12,13]. We expect these compounds to be starting points for the finding of products of pharmacological interest (Figure 2). The possibility of variations in the substitution pattern of the cyclopropane allows different configurations that could direct their selectivity to different glycosidases. Although the development of synthetic routes to iminosugars has received much attention in the synthetic community [14,15], the preferred chiral pools are carbohydrates, which are transformed using reductive aminations [16,17], or other transformation strategies [18]. In our case, we developed synthetic approaches from natural amino acids, which have less precedents [19,20]. Other asymmetric or biocatalyzed approaches have been used [21]. In our previous work, starting from natural amino acid L-serine, we synthesized iminosugars bearing the *R* configuration on the carbon adjacent to nitrogen, which was supposed to mimic C5 in natural carbohydrates and iminosugars. 

The present work is focused on new compounds prepared both from L-alanine and l-serine. In the case of L-alanine, the derived compounds could mimic 6-dehydroxylated sugars as fucose. Fucose-containing glycans, such as in blood groups and Lewis oligosaccharides and related ones, are critical for a wide range of cell events [22]. These include cell–cell adhesion, immune response, viral and bacterial infection, and tumor progression. We prepared new bicyclic iminosugars that include the cyclopropane motif fused with a piperidine that may mimic the l-fucose ring and evaluated them against fucosidase and other glycosidases. In addition, we prepared cyclopropane-containing piperidine iminosugars starting from l-serine but now with an *S* configuration at the carbon that mimics C5. A preliminary glycosidase inhibition evaluation is shown. The synthesis implies building final compounds with five stereogenic centers [23,24,25]. 

## 2. Results and Discussion

### 2.1. Chemistry

**Scheme 1:**
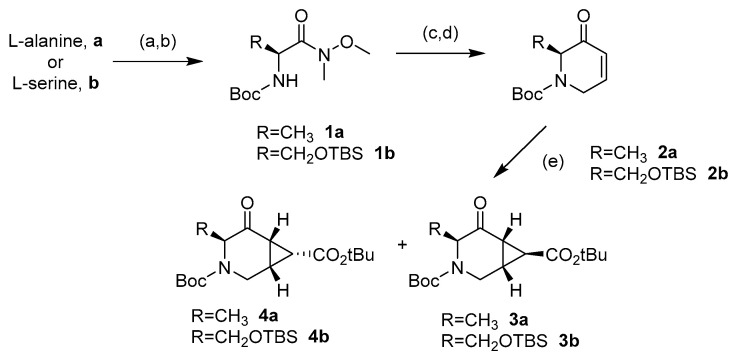
Reagents and conditions: (**a**) Boc_2_O, NaOH 1M, Dioxane, 0 °C to r.t.; then, diisopropilehtyl amine (DIPEA), *N*-(3-Dimethylaminopropyl)-*N*′-ethylcarbodiimide hydrochloride (EDCI), *N*,*O*-dimethylhydroxilamine, dichloromethane (DCM), 0 °C, 78% from l-serine (**1a**). (**b**) For the product derived from l-serine: Imidazole, 4-(dimethylamino)pyridine (DMAP), *tert*-butyldimthylsilyl chloride (TBSCl), dimethylformamide (DMF), 0 °C to r.t., 76% from l-serine (**1b**). (**c**) NaH, allyl bromide, DMF, 0 °C to 50 °C, 78% (from **1a**) and 58% (from **1b**). (**d**) Vinylmagnesium bromide, tetrahydrofurane (THF) −30 °C; then, second-generation Grubbs’ catalyst (3 mol %), DCM, 50 °C, 76% (**2a**) and 60% (**2b**). (**e**) *tert*-Butyl (tetrahydrothiophenylidene)acetate, DCM, 0 °C to r.t., 77% (**3a**), 5% (**4a**), 71% (**3b**), and 18% (**4b**).

**Figure 3:**
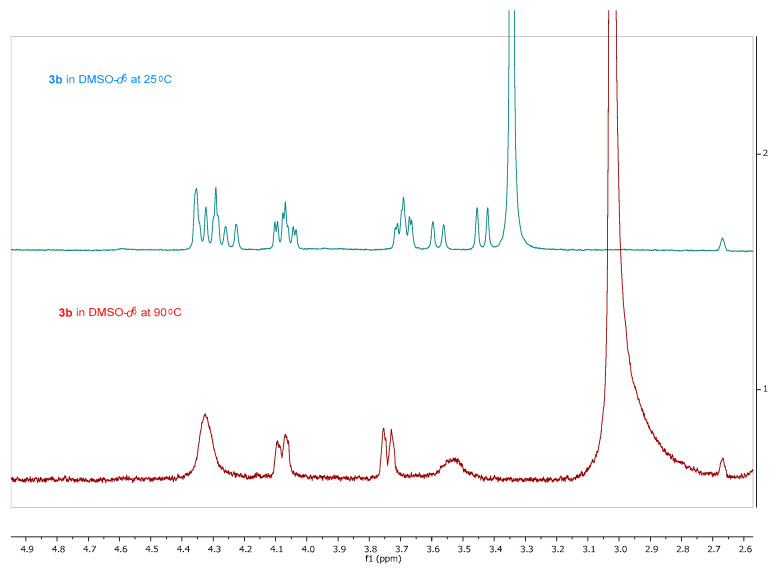
^1^H-NMR (400 MHz, CDCl_3_) of **3b** in DMSO-*d*_6_ at 25 °C (blue) and 90 °C (red).

The synthesis of the novel iminosugars started from l-serine or l-alanine as the chiral pool. Both amino acids were protected using di-*tert*-butyl dicarbonate (Boc_2_O) [26]. Without further purification, these latter intermediates were submitted to coupling with *N*,*O*-dimethylhydroxylamine and, for the l-serine derivative, protection with *tert*-butyldimethylsilyl of the hydroxy group. Thus, intermediates **1a** (78%) and **1b** (76%) were obtained in good yields. Treatment with a base and allyl bromide, followed by a reaction with vinylmagnesium bromide at −30 °C, gave the precursors of the ring-closing metathesis reaction (RCM). For the RCM, a second-generation Grubbs’ catalyst (Grubbs’ Catalyst^®^ M204, 3 mol %) was used, affording the α,β-unsaturated ketones **2a** and **2b** in 76% and 60% yield, respectively (Scheme 1) [27]. These compounds reacted with *tert*-butyl 2-(tetrahydro-1λ^4^-thiophen-1-ylidene)acetate to give a mixture (as seen in ^1^H-NMR) of cyclopropane exo:endo isomers **3a** (77%) and **4a** (5%) from **2a** in a 15:1 ratio, while **3b** (71%) and **4b** (18%) from **2b** in a 4:1 exo:endo ratio [28]. These isomers were isolated and separately characterized. ^1^H-NMR at 90 °C in DMSO-*d*_6_ determined that compounds **3b** and **4b** were obtained as a mixture of conformers due to the slow rotation of the Boc group in a 55:45 ratio in both cases (Figure 3).

The cyclopropanation reaction has two steps, ylide addition to the double bond and ring closure. It is known that the sulfur ylide attack is nonselective, and the exo:endo selectivity is determined in the second step [29,30,31]. The result depends on many different issues, such as temperature, reagent concentration, and the presence of a base. For instance, in the cyclopropanation reaction of **2a**, the 15:1 exo:endo ratio was observed when carrying out the reaction at 0 °C and a low concentration of ylide (0.15 M), while a higher temperature and ylide concentration promoted higher ratios in favor of an *exo* isomer. In the case of the cyclopropanation of compound **2b** with an ylide concentration of 2M and temperature of 20 °C, the ratio of *exo/endo* isomers can increase up to 20:1, as seen by NMR. Similar behavior has already been reported [32]. 

**Scheme 2:**
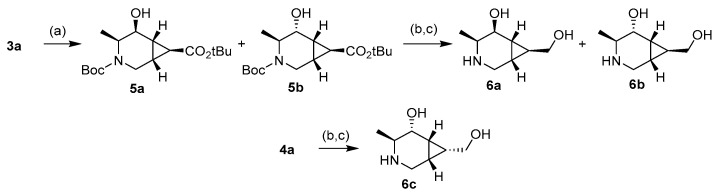
Reagents and conditions: (**a**) NaBH_4_, EtOH, 0 °C to r.t., 55% (**5a**) and 19% (**5b**). (**b**) DIBAL-H, DCM, 0 °C to r.t., 55% (from **5a**), 67% (from **5b**), and 32% (from **4a**). (**c**) Trifluoroacetic acid (TFA), MeOH, r.t., 85% (**6a**), 83% (**6b**), and 81% (**6c**).

With the intermediates **3** and **4** in hand, the final products were prepared by a reduction of the ketone and further hydrolysis or reduction of the ester group. Thus, compound **3a** was treated with NaBH_4_ to give isomers **5a** and **5b** in a 3:2 ratio (Scheme 2). These alcohols were isolated in 55% (**5a**) and 19% (**5b**) yields and separately treated with diisobutylaluminium hydride (DIBAL-H), giving the corresponding intermediates in 55% and 67% yields, respectively. A final treatment with trifluoroacetic acid (TFA) afforded compounds **6a** (85%) and **6b** (83%) after a final elution through a basic DOWEX resin. This two-step reduction allowed the separation of the isomers **5a** and **5b**. When using stronger conditions to perform the reduction in one step from **3a**, a mixture of diols was obtained but could not be separated. On the other hand, isomer **4a** reacted with DIBAL-H to give only a product in 32% yield, which, after hydrolysis with TFA and further elution through a basic DOWEX resin, afforded free amine **6c** (81%). 

**Figure 4:**
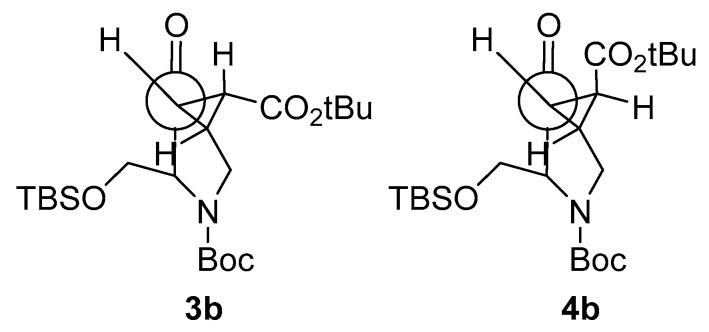
The Felkin-Anh model for compounds **3b** and **4b**.

Compound **3b** diastereoselectively reacted with NaBH_4_, resulting in **7** as the only reaction product. **4b** reacted, giving a separable mixture of compounds **8** and **9**. The selectivity of this reduction is governed by the configuration of the cyclopropane ring but not by the location of the bulky *tert*-butyldimthylsilyl (OTBS) group; as in the case of **4b**, the hydride reacts by the face of the OTBS group. The Felkin-Anh model corroborates these results: on the *exo* isomer **3b**, the *tert*-butyl ester group gets far from the carbonyl, but the OTBS is the one making the steric hindrance to determine the stereochemistry of the reaction. On the other hand, the *endo* isomer **4b** has both bulky groups near the carbonyl, staying close to the ester. Moreover, CH_2_OTBS can rotate to get farther from carbonyl, while the *tert*-butyl ester cannot (Figure 4).

**Scheme 3:**
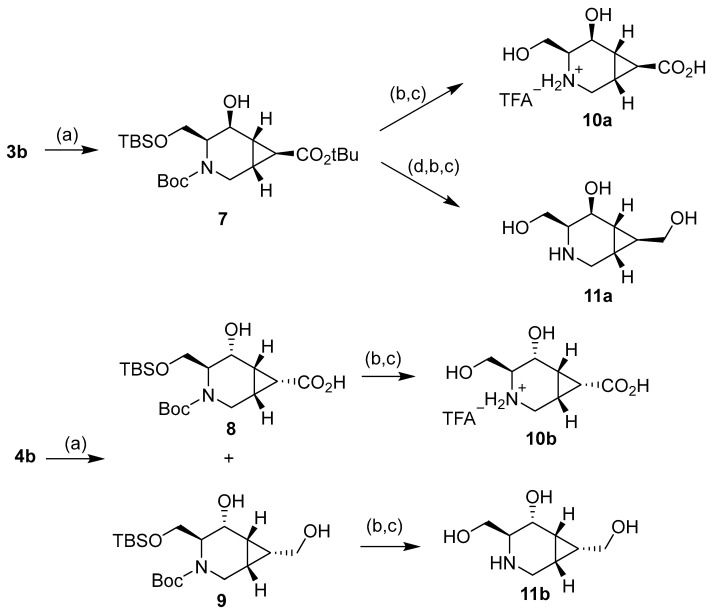
Reagents and conditions: (**a**) NaBH_4_, EtOH, 0 °C to r.t., 74% (**7**), 30% (**8**), and 50% (9). (**b**) TBAF.3H_2_O, THF, r.t., 85% (from **7** to **10a**), 68% (from **7** to **11a**), 88% (from **8**), and 86% (from **9**). (**c**) TFA, MeOH, r.t., 87% (**10a**), 91% (**11a**), 92% (**10b**), and 93% (**11b**). (**d**) DIBAL-H, DCM, 0 °C to r.t., 70%.

Deprotection of the hydroxyl group of compound **7** using tetrabutylammonium fluoride trihydrate (TBAF.3H_2_O) and further treatment with trifluoroacetic acid (TFA) resulted in the final product **10a** as a trifluoroacetate salt (Scheme 3) [33]. On the other hand, the reduction of the *tert*-butyl ester in **7** with DIBAL-H gave the *N*-Boc and OTBS protected intermediate, which was treated as previous to give the corresponding trifluoroacetate salt. After treatment of this salt with a basic DOWEX resin, the final compound **11a** was obtained. Compound **8** was deprotected to give compound **10b** as a trifluoroacetate salt. Finally, compound **9** was treated similarly to afford final compound **11b**. 

### 2.2. Modeling and NOE Experiments

**Figure 5:**
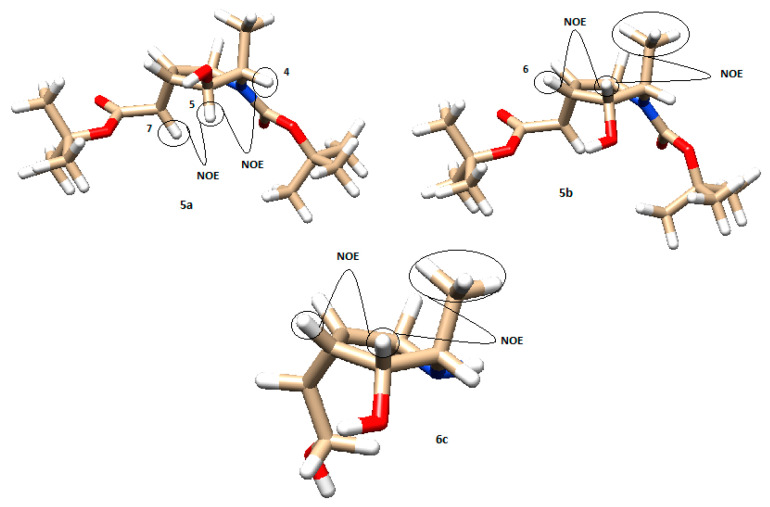
Model of compounds **5a, 5b**, and **6c** with their main NOE interactions (brown: carbon; white: hydrogen; red: oxygen; blue: nitrogen).

The stereochemistry of all the synthesized products was assigned by means of NOESY experiments and coupling constant calculations. All compounds were first modeled on Chimera 1.13.1, using ANTECHAMBER for the computing charges [34]. With these models, we could calculate the relevant dihedral angles and predict the expected NOE effects, which were checked with those experimentally obtained. Figure 5 shows the models and main NOE effects of compounds **5a** and **b** and compound **6c** derived from l-alanine. H5 in compound **5a** shows a NOE interaction with H4 and H7. On the other hand, the other reduction isomer, **5b**, gave a NOE signal between H5 and H6 and an intense effect between H5 and the methyl group. The final product **6c** obtained from the *endo* isomer, after the cyclopropanation reaction, gave analog signals as **5b**.

**Figure 6:**
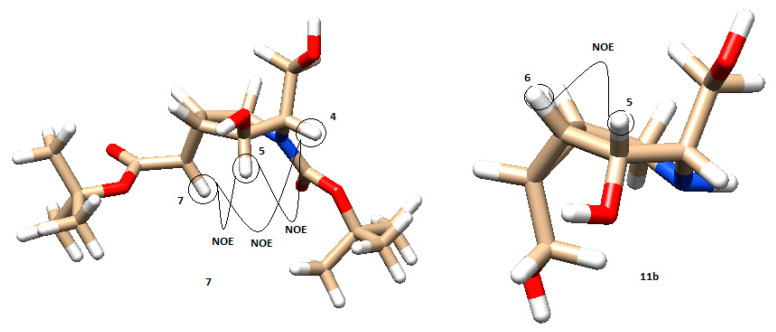
Model of compounds **7** and **11b**, respectively, with their main NOE interactions (brown: carbon; white: hydrogen; red: oxygen; blue: nitrogen).

The models and NOE interactions for compounds **7** and **11b** are shown in Figure 6. Compound **7** was assigned with the NOE effects observed between H7 and H5, H5 and H4, and between H7 and H4. The constant couplings measured in ^1^H-NMR also agreed with the modeled angles. On the other hand, compound **11b** only showed one NOE signal between H5 and H6 (Figure 6). The measured coupling constant agreed with the calculated ones from models using the Carplus equation (see Supporting Information Table S1).

### 2.3. Enzymatic Assays

Glycosidase activities were assessed in 80-μL reaction volumes in Eppendorf vials. Buffer composition and enzyme concentrations were adjusted depending on the enzyme: 20-mM Na_2_HPO_4_ at pH 7.3 for β-glucosidase from Almonds (3 μg/mL) and β-galactosidase from *Escherichia coli* (1 μg/mL), 20-mM Na_2_HPO_4_ at pH 6.8 for α-glucosidase from *Bacillus stearothermophilus* (1 μg/mL) and α-galactosidase from Green coffee (20 μM), 20-mM NaH_2_PO_4_ at pH 5.5 for α- and β-mannosidase from Jack beans and *Helix pomatia*, respectively (7 μM and 2 μM, respectively), 0.1-M NaOAc at pH 4.0 with 1 mg/mL of BSA (bovine serum albumin) for α-l-fucosidase from *Homo sapiens* (2 μM), and 50-mM NaOAc at pH 5.0 for neuraminidase from *Vibrio cholerae* (6 μM). The inhibitors were tested at 1-, 5-, and 25-mM final concentrations in the assays. Each mixture of enzyme and inhibitor was homogenized and preincubated for 10 min at 37 °C or 40 °C (α-l-fucosidase). Each reaction was initiated and brought to a final volume of 80 μL by the addition of an aliquot of the corresponding *p*-nitrophenyl glycoside substrate to obtain the following final concentrations in the reaction mixtures: *p*-nitrophenyl α- and β-d-glucopyranoside (1 mM), *p*-nitrophenyl α- and β-d-galactopyranoside (0.5 mM), *p*-nitrophenyl α- and β-d-mannopyranoside (1 mM), *p*-nitrophenyl α-l-fucopyranoside (1 mM), or *p*-nitrophenyl neuraminic acid (1 mM). After 10 min of incubation time at the same temperature, each reaction was quenched with 400 μL of 1.0-M Na_2_CO_3_, and the absorbance at 405 nm was measured. Assays were repeated twice, and the data was averaged.

The enzymatic activity was calculated by the ratio in the absorbance measured at 405 nm of the phenoxide released in the enzymatic reaction. The final compounds were screened at 1, 5, and 25 mM. The assays at 1 mM of synthesized compounds did not give clear results. Thus, the activity detected at 5 mM is shown in Table 1. 

None of the synthesized compounds showed activity against α-glucosidase, α-mannosidase, or neuraminidase at the concentrations used. In the case of products **11a** and **6a**, no activity was observed against any of the enzymes. Interestingly, products **6c** and **10b** inhibited only one enzyme, β-glucosidase, decreasing their activity to 43% and 39% at 5 mM, respectively. The results at 25 mM were 20% and 13% of the residual activity, respectively. Product **11b** inhibited the activity of β-galactosidase to 25% at 5 mM but without an inhibition increase at 25 mM. 

On the other hand, some assays showed an enhancement in the enzyme activity. Thus, compound **6b** increased the activity of β-mannosidase and α-l-fucosidase up to 148% and 142% at 5 mM, respectively. This increase raised up to 240% at 25 mM. Compound **10a** activated α-galactosidase and β-mannosidase up to around 155% at 5 mM. 

Regarding the inhibitory results, we can conclude that these compounds are very weak inhibitors only against certain enzymes far from the inhibition values of well-known iminosugars such as deoxinojirimycin [35] or castanospermine analogs [36]. However, the activation observed in certain cases deserves some comments, as there are few precedents of this behavior [37,38], including our previous results with similar compounds [10]. This activation does not have a clear explanation. The possibility that the compounds could work as efficient transglycosidation acceptors and, thus, accelerate the nitrophenol release was checked following the enzymatic reaction in a NMR tube and recorded spectra each 5 min. However, no potential transglycosilation product was detected (see the Supporting Information). Other cases of glycosidase activity enhancements were described; thus, some glycosidases were found to activate when using multivalent iminosugars [37]. Other reports explained the activation mechanism by the introduction of a small molecule in the active site, locking the reactive form of the glycosidase [38], or through an allosteric-type interaction that changed the conformation of the enzyme into the active one. These observations need further research to explain this behavior.

## 3. Materials and Methods

### 3.1. General Information

All chemicals were obtained from Aldrich/Merck (St. Louis, MO, USA), VWR (Radnor, PA, USA), Fluorochem (Derbyshire, UK), and ABCR (Karlsruhe, Germany). TLC analyses were performed on Merck silica gel 60 F254 plates using phosphomolybdic acid or anisaldehyde and heat for detection. Silica gel NORMASIL 60 40–63 μm was used for flash chromatography. NMR spectra were recorded on a Bruker spectrometer (400 MHz or 300 MHz for ^1^H and 100 MHz or 75 MHz for ^13^C), (Billerica, MA, USA). Chemical shifts are reported in δ ppm referenced to CDCl_3_ (δ = 7.26 for ^1^H and 77.00 for ^13^C), CD_3_OD (δ = 3.31 for ^1^H and 49.00 for ^13^C), or D_2_O (δ = 4.79 for ^1^H). Bidimensional spectra (HMQC, HMBC, COSY, and NOESY) were recorded in order to carry out the assignment. Infrared spectra were done in a Perkin-Elmer spectrum 100 (Agilent, Santa Clara, CA, USA). Specific optical rotation was measured in a Polarimeter Anton Paar MCP 100 (Anton Paar, Graz, Austria). Melting points of solid compounds were determined using a Stuart Scientific Melting Point Apparatus SMP3 (Stuart, Staffordshire, UK). Microanalyses were done on a LECO CHNS-932 (LECO, St. Joseph, MI, USA). Absorbance of *p*-nitrophenoxyde released in the enzymatic reactions was measured at 405 nm in a Perkin-Elmer Lamba25 (PerkinElmer, Waltham, MA, USA).

### 3.2. Synthesis

#### Synthesis of **1**–**11**

*Tert-butyl (S)-(1-(methoxy(methyl)amino)-1-oxopropan-2-yl)carbamate* (**1a**): To a solution of l-alanine (7.00 g, 78.57 mmol) in 175 mL of NaOH 1M at 0 °C was added a solution of di-*tert*-butyl dicarbonate (20.58 g, 94.29 mmol) in 77 mL of dioxane. The reaction was stirred 4 h at room temperature. The reaction was quenched with KHSO_4_ 1M until pH 1 to 2. The aqueous layer was extracted with AcOEt (3 × 150 mL). The combined organic layers were dried over MgSO_4_, and the solvent was evaporated in vacuo. The crude was dissolved in 300 mL of DCM and cooled to 0 °C. DIPEA (13.7 mL, 78.57 mmol), EDCI (15.06 g, 78.57 mmol), and *N*,*O*-dimethylhydroxilamine hydrochloride (7.67 g, 78.57 mmol) were added. The reaction was stirred at 0 °C for 1.5 h. One hundred milliliters of a solution of HCl 1M was added to the reaction. The aqueous phase was extracted with DCM (2 × 150 mL). The combined organic layers were washed with NaHCO_3_ (150 mL) and brine (150 mL) and dried over MgSO_4_. The solvent was evaporated in vacuo. A colorless oil was obtained (14.23 g, 78% after two steps). ^1^H-NMR (400 MHz, CDCl_3_) δ 5.27 (d, *J* = 8.7 Hz, 1H, NH), 4.57 (brs, 1H, CHN), 3.66 (s, 3H, OCH_3_), 3.10 (s, 3H, NCH_3_), 1.33 (s, 9H, 3×CH_3_), 1.20 (d, *J* = 6.9 Hz, 3H, CH_3_) ppm. ^13^C-NMR (100 MHz, CDCl_3_) δ 173.6, 155.1, 79.3, 67.0, 61.5, 46.4, 28.3 (3C), 18.5 ppm. IR (NaCl): 3065, 2986, 2935, 1761, 1684 cm^−1^. ${\left[\alpha \right]}_{D}^{25}$ (c 0.23 in CHCl_3_): −4.12. Anal. Calc. for C_10_H_20_N_2_O_4_: C, 51.7; H, 8.7; N, 12.1%. Found: C, 51.5; H, 9.0; N, 12.2%.

*Tert-butyl (S)-allyl(1-(methoxy(methyl)amino)-1-oxopropan-2-yl)carbamate*: To a suspension of **1a** (14.23 g, 61.30 mmol) in 200 mL of DMF at 0 °C was added slowly NaH 60% *w*/*w* (4.90 g, 122.6 mmol). After 10 min, allyl bromide was added (16 mL, 183.9 mmol). The reaction was stirred at 50 °C for 2.5 h. The reaction was quenched with 200 mL of a saturated solution of NH_4_Cl. The aqueous phase was extracted with AcOEt (2 × 350 mL). The combined organic layers were washed with a saturated solution of NH_4_Cl (150 mL), a saturated solution of NaHCO_3_ (150 mL), and brine (150 mL) and dried over MgSO_4_. The solvent was evaporated in vacuo. The crude was purified in silica gel in Hex/AcOEt (9:1). A yellow oil was obtained (12.95 g, 78%). ^1^H-NMR (400 MHz, CDCl_3_) δ 5.67–5.57 (m, 1H, HC=), 5.06–4.98 (m, 1H, CHN), 4.89–4.82 (m, 2H, =CH_2_), 3.75–3.59 (m, 2H, CH_2_N), 3.53 (s, 3H, OCH_3_), 2.94 (s, 3H, NCH_3_), 1.23 (s, 9H, 3×CH_3_), 1.09 (d, *J* = 7.2 Hz, 3H, CH_3_) ppm. Two conformers were observed in ^13^C-NMR (100 MHz, CDCl_3_) δ 172.8, 155.1 (major), 154.4 (minor), 135.8 (major), 135.2 (minor), 115.3 (minor), 114.8 (major), 79.7 (minor), 79.4 (major), 61.2 (major), 61.0 (minor), 51.5 (minor), 49.6 (major), 46.5 (minor), 45.9 (major), 32.1 (minor), 31.9 (major), 28.0 (3C), 15.2 (minor), 15.0 (major) ppm. IR (NaCl): 3065, 2986, 2935, 1761, 1684 cm^−1^. ${\left[\alpha \right]}_{D}^{25}$ (c 0.22 in DCM): −38.64. Anal. Calc. for C_13_H_24_N_2_O_4_: C, 57.3; H, 8.9; N, 10.3%. Found: C, 57.1; H, 8.7; N, 10.5%.

*Tert-butyl (S)-2-methyl-3-oxo-3,6-dihydropyridine-1(2H)-carboxylate* (**2a**): A solution of tert-butyl (S)-allyl(1-(methoxy(methyl)amino)-1-oxopropan-2-yl)carbamate (8.66 g, 31.82 mmol) in 50 mL of THF was cooled to −30 °C. A solution of vinylmagnesium bromide 0.7 M in THF (100 mL) was added slowly, keeping the temperature below −25 °C. When the addition was finished, the reaction was stirred at the same temperature for 30 min more. The reaction was poured on a mixture of 60 mL of HCl 10% and 120 mL of MeOH cooled in a bath at −15 °C. This mixture was stirred for another 15 min. The aqueous phase was extracted with AcOEt (2 × 100 mL). The combined organic layers were washed with a saturated solution of NH_4_Cl (100 mL), a saturated solution of NaHCO_3_ (100 mL), and brine (60 mL); dried over MgSO_4_; and evaporated in vacuo. The crude was dissolved in 150 mL of DCM and heated to reflux. When reflux began, Grubbs’ catalyst 2^nd^ generation (812 mg, 0.96 mmol) was added. The reaction was stirred for 1.5 h. The reaction was filtered through a pad of celite. The solvent was evaporated in vacuo. The crude was purified in silica gel in Hex/AcOEt (4:1). A yellow oil was obtained as 2 conformers in a ratio 60:40 (5.11 g, 76% after two steps). ^1^H-NMR (400 MHz, CDCl_3_) δ 6.95 (brs, 1H, CH_2_CH=major + minor), 6.05 (dt, *J* = 10.3, 2.3 Hz, 1H, COCH=, major + minor), 4.72–4.54 (m, 2H, CHN+CH_2_N, major + minor), 3.84 (brs, 1H, CH_2_N major), 3.79 (brs, 1H, CH_2_N minor), 1.44 (s, 9H, 3×CH_3_, major + minor), 1.22 (d, *J* = 7.2 Hz, 3H, CH_3_, major + minor) ppm. ^13^C-NMR (100 MHz, CDCl_3_) δ 196.8, 154.1, 146.3, 125.9, 80.9, 56.5, 39.8, 28.4 (3C), 15.9 ppm. IR (NaCl): 3058, 2979, 2931, 1748, 1681 cm^−1^. ${\left[\alpha \right]}_{D}^{25}$ (c 0.08 in DCM): +73.37. Anal. Calc. for C_11_H_17_NO_3_: C, 62.5; H, 8.1; N, 6.6%. Found: C, 62.7; H, 8.4; N, 6.4%.

*Di-tert-butyl (1R,4S,6S)-4-methyl-5-oxo-3-azabicyclo[4.1.0]heptane-3,7-dicarboxylate* (**3a** and **4a**): To a solution of **2a** (3.48 g, 16.46 mmol) in 17 mL of DCM at 0 °C was added a solution of *tert-butyl* 2-(tetrahydro-1λ^4^-thiophen-1-ylidene)acetate (9.98 g, 49.38 mmol) in 270 mL of DCM. The reaction was stirred 20 min at 0 °C and 20 more minutes at room temperature. Deionized water was added (150 mL), and layers were separated. The aqueous layer was extracted with DCM (2 × 100 mL). The combined organic layers were washed with brine (80 mL), dried over MgSO_4_, and evaporated in vacuo. The crude was purified in silica gel in Hex/AcOEt (9:1). *Exo* isomer (**3a**) was obtained as a pale brown solid (4.10 g, 77%). *Endo* isomer (**4a**) was obtained as an orange wax (280 mg, 5%). Other 2 isomers were obtained as a brown oil (10 mg, 0.2%).

Spectroscopic data for *di-tert-butyl (1R,4S,6S,7S)-4-methyl-5-oxo-3-azabicyclo[4.1.0] heptane-3,7-dicarboxylate* (*Exo-n*, **3a***)*: 2 conformers in a ratio (64:36) were observed. ^1^H-NMR (400 MHz, CDCl_3_) δ 4.56 (q, *J* = 7.2 Hz, 1H, minor, CHN), 4.42 (d, *J* = 14.1 Hz, 1H, major, CH_2_N), 4.36 (q, *J* = 7.1 Hz, 1H, major, CHN), 4.26 (d, *J* = 14.1 Hz, 1H, minor, CH_2_N), 3.29 (d, *J* = 14.1 Hz, 1H, minor, CH_2_N), 3.20 (d, *J* = 14.2 Hz, 1H, major, CH_2_N), 2.32–2.25 (m, 4H, major + minor, CHCO + CHCO_2_), 2.20–2.12 (m, 2H, major + minor, CHCH_2_N), 1.47 (s, 9H, 3×CH_3_, minor), 1.45 (s, 9H, 3×CH_3_, major), 1.43 (s, 18H, 3×CH_3_, major + minor), 1.23 (d, *J* = 7.2 Hz, 6H, major + minor, CH_3_) ppm. ^13^C-NMR (100 MHz, CDCl_3_) δ 204.1 (major), 204.0 (minor), 169.4, 154.5, 82.1 (major), 82.0 (minor), 81.2, 56.9 (major), 55.9 (minor), 36.1 (minor), 34.7 (major), 32.2, 28.4 (3C), 28.1 (3C), 24.9 (major), 24.7 (minor), 24.6 (minor), 24.4 (major), 15.9 (major), 15.4 (minor) ppm. IR (KBr): 2975, 2863, 1739, 1731, 1714 cm^−1^. ${\left[\alpha \right]}_{D}^{25}$ (c 0.16 in DCM): +88.22. m.p.: 98.5 °C–101.2 °C. Anal. Calc. for C_17_H_27_NO_5_: C, 62.8; H, 8.4; N, 4.3%. Found: C, 63.0; H, 8.2; N, 4.6%.

Spectroscopic data for *di-tert-butyl (1R,4S,6S,7R)-4-methyl-5-oxo-3-azabicyclo[4.1.0] heptane-3,7-dicarboxylate* (*Endo-n*, **4a**): 2 conformers in a ratio (93:7) were observed. ^1^H-NMR data given of the major conformer. ^1^H-NMR (400 MHz, CDCl_3_) δ4.46–4.36 (m, 2H, CHN+CH_2_N), 3.38 (d, *J* = 13.4 Hz, 1H, CH_2_N), 2.26 (t, *J* = 9.3 Hz, 1H, CHCO_2_), 2.03 (dd, *J* = 9.6, 7.8 Hz, 1H, CHCO), 1.92-1.89 (m, 1H, CHCH_2_N), 1.40 (s, 9H, 3×CH_3_), 1.38 (s, 9H, 3×CH_3_), 1.22 (d, *J* = 7.2 Hz, 3H, CH_3_) ppm. ^13^C-NMR (100 MHz, CDCl_3_) δ 204.9 (major), 202.8 (minor), 168.6 (minor), 166.9 (major), 154.6, 82.3 (major), 82.2 (minor), 80.9, 57.2 (major), 56.5 (minor), 35.0 (minor), 33.9 (major), 28.7 (minor), 28.4 (3C), 28.4 (major), 28.1 (3C, minor), 28.0 (3C, major), 25.7 (major), 25.4 (minor), 21.6, 15.9 (major), 15.0 (minor) ppm. IR (NaCl): 2982, 2874, 1737, 1728, 1715 cm^−1^. ${\left[\alpha \right]}_{D}^{25}$ (c 0.14 in DCM): +33.04. Anal. Calc. for C_17_H_27_NO_5_: C, 62.8; H, 8.4; N, 4.3%. Found: C, 63.1; H, 8.0; N, 4.2%.

*Tert-butyl (S)-(3-hydroxy-1-(methoxy(methyl)amino)-1-oxopropan-2-yl)carbamate*: To a solution of l-serine (10 g, 95.16 mmol) in 200 mL of NaOH 1M at 0 °C was added a solution of di-*tert*-butyl dicarbonate (24.74 g, 113.36 mmol) in 90 mL of dioxane. The reaction was stirred for 4 h at room temperature. The reaction was quenched with a solution of KHSO_4_ 1M until pH 1 to 2. The aqueous phase was extracted with AcOEt (3 × 300 mL). The combined organic layers were dried over MgSO_4_. The solvent was evaporated in vacuo, obtaining a colorless oil. The crude was dissolved in 360 mL of DCM and cooled to 0 °C. DIPEA (16.6 mL, 95.16 mmol), EDCI (18.24 g, 95.16 mmol), and *N*,*O*-dimethylhydroxilamine hydrochloride (9.28 g, 95.16 mmol) were added. The reaction was stirred at 0 °C for 1.5 h. One hundred milliliters of a solution of HCl 1M was added. The aqueous phase was extracted with DCM (2 × 150 mL). The combined organic layers were washed with a saturated solution of NaHCO_3_ (100 mL) and brine (100 mL), dried over MgSO_4_, and evaporated in vacuo. A white solid was obtained (21.14 g, 90% after 2 steps). ^1^H-NMR (400 MHz, CDCl_3_) δ 5.65 (d, *J* = 8.7 Hz, 1H, NH), 4.77 (brs, 1H, CHN), 3.81–3.78 (m, 2H, CH_2_O), 3.76 (s, 3H, OCH_3_), 3.21 (s, 3H, NCH_3_), 1.42 (s, 9H, 3×CH_3_) ppm. ^13^C-NMR (100 MHz, CDCl_3_) δ 171.1, 156.0, 80.1, 67.2, 63.7, 61.7, 52.5, 28.4 (3C) ppm. IR (KBr): 3378, 2984, 2868, 1740, 1708 cm^−1^. ${\left[\alpha \right]}_{D}^{25}$ (c 0.24 in CHCl_3_): +2.59. m.p.: 110.1–115.6 °C. Anal. Calc. for C_10_H_20_N_2_O_5_: C, 48.4; H, 8.1; N, 11.3%. Found: C, 48.7; H, 7.9; N, 11.6%.

*Tert-butyl (S)-(3,8,8,9,9-pentamethyl-4-oxo-2,7-dioxa-3-aza-8-siladecan-5-yl)carbamate* (**1b**): To a solution of *tert*-butyl (*S*)-(3-hydroxy-1-(methoxy(methyl)amino)-1-oxopropan-2-yl)carbamate (21.14 g, 85.19 mmol) in 70 mL of DMF at 0 °C was added imidazole (17.40 g, 255.58 mmol), DMAP (520 mg, 4.26 mmol), and TBSCl (15.41 g, 102.23 mmol). The reaction was stirred for 40 min at room temperature. Deionized water (500 mL) and AcOEt (300 mL) were added. The aqueous layer was extracted with AcOEt (2 × 150 mL). The combined organic layers were washed with water (3 × 300 mL) and brine (150 mL), dried over MgSO_4_, and evaporated in vacuo. The crude was purified in silica gel in Hex/AcOEt (9:1). A yellow oil was obtained (26.31 g, 85%). ^1^H-NMR (400 MHz, CDCl_3_) δ5.31 (d, *J* = 9.0 Hz, 1H, NH), 4.69–4.64 (m, 1H, CHN), 3.77 (dd, *J* = 10.1, 4.7 Hz, 1H, CH_2_O), 3.71 (dd, *J* = 10.0, 5.2 Hz, 1H, CH_2_O), 3.67 (s, 3H, OCH_3_), 3.13 (s, 3H, NCH_3_), 1.35 (s, 9H, 3×CH_3_), 0.78 (s, 9H, 3×CH_3_), −0.06 (s, 6H, 2×CH_3_) ppm. ^13^C-NMR (100 MHz, CDCl_3_) δ 170.7, 155.4, 79.4, 63.5, 61.4, 52.4, 32.1, 28.3 (3C), 25.8 (3C), 18.2, −5.6 (2C) ppm. IR (NaCl): 2988, 2879, 1738, 1706 cm^−1^. ${\left[\alpha \right]}_{D}^{25}$ (c 0.24 in DCM): +11.08. Anal. Calc. for C_16_H_34_N_2_O_5_Si: C, 53.0; H, 9.5; N, 7.7%. Found: C, 53.1; H, 9.8; N, 7.6%.

*Tert-butyl (S)-allyl(3,8,8,9,9-pentamethyl-4-oxo-2,7-dioxa-3-aza-8-siladecan-5-yl)carbamate*: To a suspension of NaH 60% *w*/*w* (4.35g, 108.86 mmol) at 0 °C in 90 mL of DMF was added a solution of **1b** (19.72 g, 54.53 mmol) in 100 mL of DMF. Then, allyl bromide (16 mL, 183.9 mmol) was added. The reaction was stirred for 30 min at room temperature and 2 h at 50 °C. A solution of saturated NH_4_Cl was added, until all salts were dissolved. The aqueous layer was extracted with AcOEt (3 × 150 mL). The combined organic layers were washed with a saturated solution of NH_4_Cl (150 mL) and a saturated solution of NaHCO_3_ (150 mL) and brine (100 mL), dried over MgSO_4_, and evaporated in vacuo. The crude was purified in silica gel in Hex/AcOEt (9:1). A yellow oil was obtained (12.81 g, 58%). ^1^H-NMR (300 MHz, CDCl_3_) δ 5.92–5.71 (m, 1H), 5.34–4.93 (m, 3H), 4.02–3.76 (m, 4H), 3.72 (s, 3H), 3.15 (s, 3H), 1.43 (s, 9H), 0.86 (s, 9H), 0.04 (s, 6H) ppm. ^13^C-NMR (75 MHz, CDCl_3_) δ 170.6, 155.6 (major), 155.0 (minor), 136.0 (major), 135.3 (minor), 115.4 (minor), 115.1 (major), 80.1 (minor), 79.8 (major), 61.6, 61.0 (minor), 60.7 (major), 56.9 (minor), 55.5 (major), 46.5, 32.2, 28.4 (3C), 25.8 (3C), 18.2 (minor), 18.1 (major), −5.5 (2C) ppm. IR (NaCl): 3024, 2992, 2889, 1731, 1710 cm^−1^. ${\left[\alpha \right]}_{D}^{25}$ (c 0.26 in DCM): −35.63. Anal. Calc. for C_19_H_38_N_2_O_5_Si: C, 56.7; H, 9.5; N, 7.0%. Found: C, 56.3; H, 9.2; N, 7.3%.

*Tert-butyl-(S)-2-(((tert-butyldimethylsilyl)oxy)methyl)-3-oxo-3,6-dihydropyridine-1(2H)-carboxylate* (**2b**): A solution of tert-butyl(*S*)-allyl(3,8,8,9,9-pentamethyl-4-oxo-2,7-dioxa-3-aza-8-siladecan-5-yl)carbamate (2.18 g, 5.42 mmol) in THF (10 mL) was cooled to −30 °C. A solution of vinylmagnesium bromide 1M (12 mL) was added slowly, keeping the temperature below −25 °C. When the addition finished, the reaction was stirred for a further 30 min. The reaction was poured into a mixture of 10mL of HCl 10% and MeOH (20mL) cooled in a bath at −15 °C. This mixture was stirred for another 30 min at −15 °C. The aqueous layer was extracted with AcOEt (3 × 40 mL). The combined organic layers were washed with a saturated solution of NH_4_Cl (40 mL), a saturated solution of NaHCO_3_ (40 mL), and brine (40 mL). The organic phase was dried over MgSO_4_ and evaporated in vacuo. The crude mixture was dissolved in 9 mL of DCM and heated to reflux. Grubbs’ 2nd generation catalyst (179 mg, 0.21 mmol) was then added. The reaction was stirred for 1.5 h. The reaction was filtered through a pad of celite. The solvent was evaporated in vacuo, and the crude was purified in silica gel in Hex:AcOEt (9:1). An orange solid was obtained as 2 conformers in the ratio 63:37 (1.11 g, 60% after 2 steps). ^1^H-NMR (400 MHz, CDCl_3_) δ 7.01 (ddd, *J* = 10.4, 5.0, 2.0 Hz, 1H, =CHCH_2_, major), 6.90 (ddd, *J* = 10.4, 5.0, 2.0 Hz, 1H, =CHCH_2_, minor), 6.17 (d, *J* = 10.4 Hz, 2H, =CHCO, major + minor), 4.69 –4.60 (m, 2H, CH_2_N, major and CHN, minor), 4.55–4.47 (m, 2H, CH_2_N, minor and CHN, major), 4.00–3.92 (m, 5H, CH_2_N, major + minor and CH_2_O, major + minor), 3.82 (dd, *J* = 10.2, 3.1 Hz, 1H, CH_2_O, major), 1.49 (s, 9H, 3×CH_3_, minor), 1.47 (s, 9H, 3×CH_3_, major), 0.81 (s, 18H, 3×CH_3_, major + minor), −0.02 (s, 6H, CH_3_, major + minor), −0.04 (s, 6H, CH_3_, major + minor) ppm. ^13^C-NMR (100 MHz, CDCl_3_) δ 195.5, 154.4 (major), 154.3 (minor), 147.4 (major), 146.2 (minor), 127.8 (minor), 127.6 (major), 81.0 (major), 80.9 (minor), 65.9 (major), 65.7 (minor), 62.8 (major), 61.7 (minor), 44.2 (minor), 43.1 (major), 28.5 (3C), 25.9 (3C), 18.2, −5.6 (minor, 2C), −5.7 (major, 2C) ppm. IR (KBr): 3044, 2987, 2895, 1741, 1696 cm^−1^. ${\left[\alpha \right]}_{D}^{25}$ (c 0.14 in DCM): +84.93. MP: 50.8 °C –55.6 °C. Anal. Calc. for C_17_H_31_NO_4_Si: C, 59.8; H, 9.2; N, 4.1%. Found: C, 60.0; H, 9.4; N, 3.9%.

*Di-tert-butyl (1S,4S,6R)-4-(((tert-butyldimethylsilyl)oxy)methyl)-5-oxo-3-azabicyclo[4.1.0] heptane-3,7-dicarboxylate* (**3b** and **4b**): To a solution of 2b (1.11 g, 3.25 mmol) in 3 mL of DCM at 0 °C was slowly added a solution of tert-butyl 2-(tetrahydro-1λ^4^-thiophen-1-ylidene)acetate (1.97 g, 9.75 mmol) in 55 mL of DCM. The reaction was stirred for 20 min at 0 °C and 20 more minutes at room temperature. Deionized water was added (50 mL), and the layers were separated. The aqueous phase was extracted with DCM (2 × 50 mL). The combined organic layers were washed with brine (50 mL), dried over MgSO_4_, and the solvent was evaporated in vacuo. The crude was separated in silica gel in Hex:AcOEt (9:1). *Exo* isomer was obtained as 2 conformers in a ratio 58:42 as an orange solid (1.05 g, 71%). *Endo* isomer was obtained as 2 conformers in a ratio 52:48 as a brown wax (260 mg, 18%).

Spectroscopic data for *di-tert-butyl (1R,4S,6S,7S)-4-(([tert-butyldimethylsilyl]oxy)methyl)-5-oxo-3-azabicyclo[4.1.0]heptane-3,7-dicarboxylate* (**3b**): ^1^H-NMR (400 MHz, CDCl_3_) δ4.47 (dd, *J* = 13.5, 1.9 Hz, 1H, CH_2_N, major), 4.38 (t, *J* = 2.9 Hz, 1H, CHN, minor), 4.29 (dd, *J* = 13.6, 2.0 Hz, 1H, CH_2_N, minor), 4.21 (t, *J* = 3.0 Hz, 1H, CHN, major), 4.15 (dt, *J* = 10.0, 3.1 Hz, 2H, CH_2_O, major + minor), 3.79 (dd, *J* = 10.2, 2.7 Hz, 1H, CH_2_O, minor), 3.74 (d, *J* = 12.4 Hz, 1H, CH_2_N, minor), 3.71 (dd, *J* = 10.1, 2.7 Hz, 1H, CH_2_O, major), 3.63 (dd, *J* = 13.6, 1.8 Hz, 1H, CH_2_N, major), 2.34–2.31 (m, 2H, CHCO, major + minor), 2.19 (t, *J* = 4.4 Hz, 2H, CHCO_2_, major + minor), 2.18–2.09 (m, 2H, CHCH_2_N, major + minor), 1.42 (s, 18H, 3×CH_3_, major + minor), 1.415 (s, 9H, 3×CH_3_, minor), 1.411 (s, 9H, 3×CH_3_, major), 0.82 (s, 18H, 3×CH_3_, major + minor), −0.02 (s, 12H, 2×CH_3_, major + minor) ppm. ^13^C-NMR (100 MHz, CDCl_3_) δ 202.5 (major), 202.3 (minor), 169.5, 155.0 (minor), 154.7 (major), 82.0 (major), 81.9 (minor), 81.1, 66.0 (major), 65.6 (minor), 62.4 (major), 61.5 (minor), 39.4 (minor), 37.9 (major), 32.90 (minor), 32.87 (major), 28.5 (3C), 28.10 (3C, minor), 28.08 (3C, major), 25.9 (3C, minor), 25.8 (3C, major), 24.8 (major), 24.7 (minor), 24.6 (minor), 24.4 (major), 18.16, −5.5 (2C, minor), −5.7 (2C, major) ppm. IR (KBr): 2991, 2887, 1724, 1711, 1691 cm^−1^. ${\left[\alpha \right]}_{D}^{25}$ (c 0.15 in DCM): +6.60. m.p.: 74.2 °C–79.0 °C. Anal. Calc. for C_23_H_41_NO_6_Si: C, 60.6; H, 9.1; N, 3.1%. Found: C, 60.3; H, 9.4; N, 3.3%.

Spectroscopic data for *di-tert-butyl (1R,4S,6S,7R)-4-(([tert-butyldimethylsilyl]oxy)methyl)-5-oxo-3-azabicyclo[4.1.0]heptane-3,7-dicarboxylate* (**4b**): ^1^H-NMR (400 MHz, CDCl_3_) δ 4.54 (d, *J* = 13.4 Hz, 1H, CH_2_N, major), 4.43 (d, *J* = 13.2 Hz, 1H, CH_2_N, minor), 4.24 (t, *J* = 2.7 Hz, 1H, CHN, minor), 4.21–4.12 (m, 3H, CHN, major and CH_2_O, major + minor), 3.92–3.85 (m, 2H, CH_2_N, minor and CH_2_O, minor), 3.82 (dd, *J* = 13.5, 3.2 Hz, 1H, CH_2_N, major), 3.77 (dd, *J* = 10.4, 2.9 Hz, 1H, CH_2_O, major), 2.29–2.20 (m, 2H, CHCO, major + minor), 2.09 (t, *J* = 8.6 Hz, 2H, CHCO_2_, major + minor), 1.95–1.81 (m, 2H, CHCH_2_N, major + minor), 1.45 (s, 9H, 3×CH_3_, minor), 1.40 (s, 9H, 3×CH_3_, major), 1.39 (s, 9H, 3×CH_3_, major), 1.38 (s, 9H, 3×CH_3_, minor), 0.84 (s, 18H, 3×CH_3_, major + minor), 0.00 (s, 6H, CH_3_, major + minor), −0.01 (s, 6H, CH_3_, major + minor) ppm. ^13^C-NMR (100 MHz, CDCl_3_) δ 204.0 (major), 203.4 (minor), 167.4 (major), 167.0 (minor), 154.7 (minor), 154.6 (major), 82.5 (minor), 82.1 (major), 80.9 (minor), 80.7 (major), 67.5 (major), 66.3 (minor), 62.8 (major), 62.0 (minor), 38.5 (minor), 37.5 (major), 28.9, 28.6 (3C,), 28.2 (3C, major), 28.0 (3C, minor), 27.8, 25.91 (3C, minor), 25.89 (3C major), 22.0 (major), 21.9 (minor), 18.16 (minor), 18.13 (major), −5.6 (major + minor), −5.7 (major + minor) ppm. IR (NaCl): 2995, 2894, 1721, 1716, 1699 cm^−1^. ${\left[\alpha \right]}_{D}^{25}$ (c 0.09 in DCM): +34.09. Anal. Calc. for C_23_H_41_NO_6_Si: C, 60.6; H, 9.1; N, 3.1%. Found: C, 60.1; H, 8.9; N, 3.4%.

*Di-tert-butyl (1R,4S,6S,7S)-5-hydroxy-4-methyl-3-azabicyclo[4.1.0]heptane-3,7-dicarboxylate* (**5a** and **5b**): To a solution of **3a** (4.10 g, 12.61 mmol) in absolute ethanol (90 mL) at 0 °C was added NaBH_4_ (954 mg, 25.22 mmol). The reaction was stirred for 1 h at room temperature. Then, a saturated solution of NH_4_Cl was added (until salts were dissolved). The aqueous phase was extracted with AcOEt (3 × 50 mL). The combined organic layers were washed with brine (60 mL). A solid was obtained as a mixture of two isomers in a 3:2 ratio, that were separated by silica gel column chromatography using Hex:AcOEt (5:1) to Hex/AcOEt (2:1) as eluents (3.10 g, 55% (**5a**) and 19% (**5b**)).

Spectroscopic data from *di-tert-butyl (1R,4S,5S,6S,7S)-5-hydroxy-4-methyl-3-azabicyclo [4.1.0]heptane-3,7-dicarboxylate* (**5a**): White solid (2.27 g, 55%).^1^H-NMR (400 MHz, CDCl_3_) δ 4.16 (brs, 1H, CHN), 4.01 (d, *J* = 14.1 Hz, 1H, CH_2_N), 3.84 (td, *J* = 5.9, 5.4, 2.0 Hz, 1H, C**H**OH), 3.28 (dd, *J* = 14.1, 4.1 Hz, 1H, CH_2_N), 1.63–1.58 (m, 1H, CHCH_2_N), 1.55 (ddd, *J* = 9.4, 4.5, 2.0 Hz, 1H, CHCHOH), 1.434 (s, 9H, 3×CH_3_), 1.431 (s, 9H, 3×CH_3_), 1.22 (t, *J* = 4.6 Hz, 1H, CHCO_2_), 1.15 (d, *J* = 6.9 Hz, 3H, CH_3_) ppm. ^13^C-NMR (100 MHz, CDCl_3_) δ 172.6, 155.1, 80.9, 80.3, 67.5, 48.4, 36.1, 28.6 (3C), 28.3 (3C), 25.2, 25.0, 20.2, 11.4 ppm. IR (KBr): 3389, 2986, 2884, 1727, 1720 cm^−1^. ${\left[\alpha \right]}_{D}^{25}$ (c 0.13 in DCM): +7.43. m.p.: 123.8 °C–127.1 °C. Anal. Calc. for C_17_H_29_NO_5_: C, 62.4; H, 8.9; N, 4.3%. Found: C, 62.6; H, 9.2; N, 4.1%.

Spectroscopic data from *di-tert-butyl (1R,4S,5R,6S,7S)-5-hydroxy-4-methyl-3-azabicyclo [4.1.0]heptane-3,7-dicarboxylate* (**5b**): White solid (770 mg, 19%).^1^H-NMR (400 MHz, CDCl_3_) δ 4.15–4.08 (m, 2H, CHN+CH_2_N), 3.99–3.97 (m, 1H, CHOH), 3.20 (d, *J* = 13.8 Hz, 1H, CH_2_N), 1.95–1.90 (m, 1H, CHCHOH), 1.67–1.63 (m, 2H, 2×CH cyclopropane), 1.46 (s, 9H, 3×CH_3_), 1.44 (s, 9H, 3×CH_3_), 1.13 (d, *J* = 7.2 Hz, 3H, CH_3_) ppm. ^13^C-NMR (100 MHz, CDCl_3_) δ 172.9, 155.9, 80.8, 80.3, 66.3, 51.3, 35.4, 28.5 (3C), 28.3 (3C), 23.6, 20.6, 19.7, 16.1 ppm. IR (KBr): 3395, 2974, 2891, 1729, 1718 cm^−1^. ${\left[\alpha \right]}_{D}^{25}$ (c 0.12 in DCM): −2.61. m.p.: 96.0 °C–98.6 °C. Anal. Calc. for C_17_H_29_NO_5_: C, 62.4; H, 8.9; N, 4.3%. Found: C, 62.2; H, 9.1; N, 4.0%.

*Tert-butyl (1S,4S,6S,7S)-5-hydroxy-7-(hydroxymethyl)-4-methyl-3-azabicyclo[4.1.0]heptane-3-carboxylate*: To a solution of **5a** (2.172 g, 6.62 mmol) in 55 mL of DCM cooled to 0 °C was added DIBAL-H 1.2M in toluene (22 mL). The reaction was stirred at room temperature for 1 h. The solvent was evaporated in vacuo. The crude was purified in silica gel in Hex:AcOEt (1:3). A yellow wax was obtained as two conformers in a ratio 1:1 (931 mg, 55%). ^1^H-NMR (400 MHz, CDCl_3_) δ4.05 (brs, 1H, CHN), 3.81 (dd, *J* = 13.8, 1.7 Hz, 1H, CH_2_N), 3.76–3.69 (m, 2H, CH_2_O+CHOH), 3.33 (dd, *J* = 13.8, 4.7 Hz, 1H, CH_2_N), 3.21 (t, *J* = 9.7 Hz, 1H, CH_2_O), 1.42 (s, 9H, 3×CH_3_), 1.14 (d, *J* = 6.7 Hz, 3H, CH_3_), 0.97-0.88 (m, 2H, 2×CH cyclopropane), 0.81–0.75 (m, 1H, CHCH_2_O) ppm. ^13^C-NMR (100 MHz, CDCl_3_) δ 155.4, 80.1, 68.6 (β), 68.5 (α), 65.5 (β), 65.4 (α), 48.7, 37.0, 28.6 (3C), 25.2, 20.23 (β), 20.19 (α), 14.7, 11.8 ppm. IR (NaCl): 3402, 2974, 2884, 1729 cm^−1^. ${\left[\alpha \right]}_{D}^{25}$ (c 0.17 in DCM): +14.68. Anal. Calc. for C_13_H_23_NO_4_: C, 60.7; H, 9.0; N, 5.4%. Found: C, 60.3; H, 9.2; N, 5.7%.

*(1S,4S,5S,6S,7S)-7-(hydroxymethyl)-4-methyl-3-azabicyclo[4.1.0]heptan-5-ol* (**6a**): To a solution of tert-butyl (1S,4S,6S,7S)-5-hydroxy-7-(hydroxymethyl)-4-methyl-3-azabicyclo[4.1.0]heptane-3-carboxylate (931 mg, 3.62 mmol) in 0.5 mL of DCM and 1 mL of MeOH was added 30 mL of TFA. The reaction was stirred 30 min at room temperature. The solvent was evaporated in vacuo. The crude was eluted through a basic DOWEX resin. A brown wax was obtained (484 mg, 85%). ^1^H-NMR (300 MHz, MeOD) δ 4.09 (t, *J* = 1.7 Hz, 1H), 3.64 (dd, *J* = 13.6, 8.5 Hz, 1H), 3.48 (dd, *J* = 11.4, 6.0 Hz, 1H), 3.40 (dd, *J* = 11.4, 6.5 Hz, 1H), 3.13 (dd, *J* = 13.6, 2.5 Hz, 1H), 2.94 (qd, *J* = 6.6, 1.7 Hz, 1H), 1.34 (ddd, *J* = 9.1, 5.2, 1.7 Hz, 1H), 1.28 (d, *J* = 6.6 Hz, 3H), 1.25–1.18 (m, 1H), 1.18–1.07 (m, 1H) ppm. ^13^C-NMR (75 MHz, MeOD) δ 65.0, 64.8, 52.2, 44.0, 26.7, 23.9, 15.3, 11.4 ppm. IR (NaCl): 3419, 2986, 2899 cm^−1^. ${\left[\alpha \right]}_{D}^{25}$ (c 0.04 in MeOH): +10.75. Anal. Calc. for C_8_H_15_NO_2_: C, 61.1%; H, 9.1; N, 8.9%. Found: C, 61.2; H, 9.4; N, 8.7%.

*Tert-butyl (1S,4S,5R,6S,7S)-5-hydroxy-7-(hydroxymethyl)-4-methyl-3-azabicyclo[4.1.0] heptane-3-carboxylate*: To a solution of **5b** (770 mg, 2.34 mmol) in 55 mL of DCM cooled to 0 °C was added DIBAL-H 1.2M in toluene (22 mL). The reaction was stirred at room temperature for 1 h. The solvent was evaporated in vacuo. The crude was purified in silica gel in Hex:AcOEt (1:3). A yellow wax was obtained (400 mg, 67%). ^1^H-NMR (400 MHz, CDCl_3_) δ 4.19–3.98 (m, 3H, OH+CH_2_N+CHN), 3.93 (d, *J* = 7.2 Hz, 1H, CHOH), 3.87 (dd, *J* = 11.2, 4.8 Hz, 1H, CH_2_O), 3.13 (d, *J* = 13.0 Hz, 1H; CH_2_N), 3.00 (dd, *J* = 11.2, 9.1 Hz, 1H, CH_2_O), 2.66 (brs, 1H, OH), 1.43 (s, 9H, 3×CH_3_), 1.28–1.23 (m, 1H, CHCHOH), 1.17–1.12 (m, 1H, CHCH_2_N), 1.09 (d, *J* = 7.1 Hz, 3H, CH_3_), 0.97–0.92 (m, 1H, CHCH_2_OH) ppm. ^13^C-NMR (100 MHz, CDCl_3_) δ 156.1, 80.1, 66.9, 65.6, 51.2, 35.7, 28.5 (3C), 19.7, 18.6, 16.5, 15.3 ppm. IR (NaCl): 3409, 2991, 2892, 1726 cm^−1^. ${\left[\alpha \right]}_{D}^{25}$ (c 0.14 in DCM): −6.84. Anal. Calc. for C_13_H_23_NO_4_: C, 60.7; H, 9.0; N, 5.4%. Found: C, 60.3; H, 9.2; N, 5.2%.

*(1S,4S,5R,6S,7S)-7-(hydroxymethyl)-4-methyl-3-azabicyclo[4.1.0]heptan-5-ol* (**6b**): To a solution of tert-butyl (1S,4S,5R,6S,7S)-5-hydroxy-7-(hydroxymethyl)-4-methyl-3-azabicyclo[4.1.0] heptane-3-carboxylate (400 mg, 1.55 mmol) in 1 mL of DCM and 2 mL of MeOH was added 12 mL of TFA. The reaction was stirred for 30 min at room temperature. The solvent was evaporated in vacuo. The crude was eluted through a basic DOWEX resin. A brown wax was obtained (202 mg, 83%). ^1^H-NMR (400 MHz, MeOD) δ 3.98 (dd, *J* = 9.5, 5.4 Hz, 1H, CHOH), 3.62–3.54 (m, 2H, CH_2_O+CH_2_N), 3.35-3.27 (m, 1H, CH_2_O), 3.03–2.99 (m, 1H, CH_2_N), 2.63 (dq, *J* = 9.5, 6.4 Hz, 1H, CHN), 1.42–1.38 (m, 2H, 2×CH cyclopropane), 1.34–1.30 (m, 4H, CH_3_+C**H**CH_2_OH) ppm. ^13^C-NMR (100 MHz, MeOD) δ 68.2, 65.0, 53.4, 43.3, 26.6, 21.9, 15.9, 15.7 ppm. IR (NaCl): 3391, 2995, 2899 cm^−1^. ${\left[\alpha \right]}_{D}^{25}$ (c 0.03 in MeOH): −51.91. Anal. Calc. for C_8_H_15_NO_2_: C, 61.1%; H, 9.6; N, 8.9%. Found: C, 60.8; H, 9.4; N, 9.1%.

*Tert-butyl (1S,4S,5R,6S,7R)-5-hydroxy-7-(hydroxymethyl)-4-methyl-3-azabicyclo[4.1.0]heptane-3-carboxylate*: To a solution of **4a** (280 mg, 0.86 mmol) in 10 mL of DCM at 0 °C was added DIBAL-H 1.2M in toluene (3.6 mL). The reaction was stirred 1 h at room temperature. Methanol was added (12 mL). The salts were filtered through a pad of celite and rinsed with methanol. The solvent was evaporated in vacuo and purified in silica gel in Hex:AcOEt (1:2). A yellow wax was obtained (71 mg, 32%). ^1^H-NMR (400 MHz, CDCl_3_) δ 4.24–4.18 (m, 2H, CHN+CHOH), 3.94 (d, *J* = 14.3 Hz, 1H, CH_2_N), 3.89 (dd, *J* = 11.6, 5.9 Hz, 1H, CH_2_O), 3.54 (t, *J* = 11.4 Hz, 1H, CH_2_O), 3.31 (brs, 1H, OH), 3.25 (dd, *J* = 14.2, 5.7 Hz, 1H, CH_2_N), 2.02 (brs, 1H, OH), 1.43 (s, 10H, 3×CH_3_+CHCHOH), 1.33–1.26 (m, 1H, C**H**CH_2_OH), 1.14–1.11 (m, 4H, CH_3_+CHCH_2_OH) ppm. ^13^C-NMR (100 MHz, CDCl_3_) δ 155.5, 80.2, 68.1, 58.8, 51.2, 34.3, 28.6 (3C), 21.0, 16.0, 14.5, 11.3 ppm. IR (NaCl): 3392, 2981, 2896, 1719 cm^−1^. ${\left[\alpha \right]}_{D}^{25}$ (c 0.01 in DCM): +60.0. Anal. Calc. for C_13_H_23_NO_4_: C, 60.7; H, 9.0; N, 5.4%. Found: C, 60.5; H, 9.3; N, 5.1%.

*(1S,4S,5R,6S,7R)-7-(hydroxymethyl)-4-methyl-3-azabicyclo[4.1.0]heptan-5-ol* (**6c**): To a solution of tert-butyl (1S,4S,5R,6S,7R)-5-hydroxy-7-(hydroxymethyl)-4-methyl-3-azabicyclo[4.1.0]heptane-3-carboxylate (112 mg, 0.44 mmol) in 0.5 mL of DCM and 0.5 mL of MeOH was added 4 mL of TFA. The reaction was stirred for 30 min at room temperature. The solvent was evaporated in vacuo. The crude was eluted through a basic DOWEX resin. A brown wax was obtained (56 mg, 81%). ^1^H-NMR (400 MHz, MeOD) δ 4.08 (dd, *J* = 9.8, 7.2 Hz, 1H, CHOH), 3.87 (dd, *J* = 12.5, 7.2 Hz, 1H, CH_2_O), 3.80 (dd, *J* = 12.5, 9.2 Hz, 1H, CH_2_O), 3.69 (dd, *J* = 14.3, 9.6 Hz, 1H, CH_2_N), 2.95 (dd, *J* = 14.3, 5.1 Hz, 1H, CH_2_N), 2.60 (dq, *J* = 9.7, 6.3 Hz, 1H, CHN), 1.75 (qd, *J* = 9.4, 5.1 Hz, 1H, CHCH_2_N), 1.60 (td, J, 9.2, 7.2 Hz, 1H, CHCHOH), 1.53–1.44 (m, 1H, CHCH_2_OH), 1.39 (d, *J* = 6.3 Hz, 3H, CH_3_) ppm. ^13^C-NMR (100 MHz, MeOD) δ 69.3, 58.4, 56.0, 40.1, 24.7, 18.7, 17.2, 14.8 ppm. IR (NaCl): 3413, 2992, 2905 cm^−1^. ${\left[\alpha \right]}_{D}^{25}$ (c 0.01 in MeOH): −72.95. Anal. Calc. for C_8_H_15_NO_2_: C, 61.1%; H, 9.6; N, 8.9%. Found: C, 61.3; H, 9.9; N, 8.7%.

*Di-tert-butyl (1R,4S,5S,6S,7S)-4-(((tert-butyldimethylsilyl)oxy)methyl)-5-hydroxy-3-azabicyclo [4.1.0]heptane-3,7-dicarboxylate* (**7**): To a solution of **3b** (970 mg, 2.13 mmol) in 11 mL of absolute ethanol at 0 °C was added NaBH_4_ (161 mg, 4.26 mmol). The reaction was stirred for 1 h at room temperature. A saturated solution of NH_4_Cl was added (30 mL). The aqueous phase was extracted with AcOEt (3 × 40 mL). The combined organic layers were washed with brine (40 mL). Only one isomer was observed, which was purified in silica gel in Hex:AcOEt (4:1). A yellow wax was obtained (716 mg, 74%). ^1^H-NMR (400 MHz, CDCl_3_) δ 4.16 (brs, 1H, CHOH), 4.06–3.90 (m, 3H, CH_2_N+CH_2_O+CHN), 3.89–3.78 (m, 1H, CH_2_O), 3.34 (brs, 1H, CH_2_N), 1.78 (dt, *J* = 9.3, 3.8 Hz, 1H, CHCHOH), 1.71–1.65 (m, 1H, CHCH_2_N), 1.44 (s, 9H, 3×CH_3_), 1.43 (s, 9H, 3×CH_3_), 1.37 (t, *J* = 4.1 Hz, 1H, CHCO_2_), 0.89 (s, 9H, 3×CH_3_), 0.083 (s, 3H, CH_3_), 0.076 (s, 3H, CH_3_) ppm. ^13^C-NMR (100 MHz, CDCl_3_) δ 172.1, 155.2, 80.8, 80.4, 67.2, 61.9, 53.4, 37.2, 28.6 (3C), 28.2 (3C), 25.9 (3C), 25.3, 23.8, 20.2, 18.1, −5.4 (2C) ppm. IR (NaCl): 3429, 2988, 2892, 1714, 1696 cm^−1^. ${\left[\alpha \right]}_{D}^{25}$ (c 0.02 in DCM): +16.49. Anal. Calc. for C_23_H_43_NO_6_Si: C, 60.4; H, 9.5; N, 3.1%. Found: C, 60.6; H, 9.4; N, 3.4%.

*Di-tert-butyl (1R,4S,5S,6S,7S)-5-hydroxy-4-(hydroxymethyl)-3-azabicyclo [4.1.0]heptane-3,7-dicarboxylate*: To a solution of **7** (300 mg, 0.66 mmol) in 3 mL of THF was added TBAF.3H_2_O (303 mg, 0.96 mmol). The reaction was stirred for 1 h at room temperature. The solvent was evaporated in vacuo and purified in silica gel in Hex/AcOEt (1:1). A white solid was obtained (192 mg, 85%). ^1^H-NMR (400 MHz, MeOD) δ 4.14 (brs, 1H, CHN), 4.10–4.01 (m, 1H, CH_2_N), 3.92 (dd, *J* = 6.1, 1.7 Hz, 1H, CHOH), 3.88-3.84 (m, 1H, CH_2_O), 3.73 (dd, *J* = 11.5, 9.7 Hz, 1H, CH_2_O), 3.42–3.31 (m, 1H, CH_2_N), 1.66–1.59 (m, 1H, CHCH_2_N), 1.56 (ddd, *J* = 9.4, 4.5, 2.1 Hz, 1H, CHCHOH), 1.46 (s, 9H, 3×CH_3_), 1.44 (s, 9H, 3×CH_3_), 1.20 (t, *J* = 4.5 Hz, 1H, CHCO_2_) ppm. ^13^C-NMR (100 MHz, MeOD) δ 172.7, 156.1, 80.6, 80.2, 65.7 (major), 65.5 (minor), 56.7, 54.9 (major), 53.7 (minor), 36.6 (minor), 35.4 (major), 27.2 (3C), 26.9 (3C), 25.4, 24.4, 20.5 (minor), 20.3 (major) ppm. IR (NaCl): 3435, 2991, 2884, 1716, 1692 cm^−1^. ${\left[\alpha \right]}_{D}^{25}$ (c 0.02 in MeOH): +6.02. m.p.: 173.8 °C –172.5 °C. Anal. Calc. for C_17_H_29_NO_6_: C, 59.5; H, 8.5; N, 4.1%. Found: C, 59.2; H, 8.8; N, 4.2%.

*(1R,4S,5S,6S,7S)-7-carboxy-5-hydroxy-4-(hydroxymethyl)-3-azabicyclo[4.1.0]heptan-3-ium trifluoroacetate* (**10a**): To a solution of di-*tert*-butyl (1*R*,4*S*,5*S*,6*S*,7*S*)-5-hydroxy-4-(hydroxymethyl)-3-azabicyclo[4.1.0]heptane-3,7-dicarboxylate (192mg, 0.56mmol) in a mixture of 3 mL of DCM and 0.7 mL of MeOH was added TFA (5.6 mL). The reaction was stirred for 20 min. The solvent was evaporated in vacuo. A brown wax was obtained (140 mg, 87%). ^1^H-NMR (400 MHz, D_2_O) δ 4.47 (brs, 1H, CHOH), 3.89–3.81 (m, 2H, CH_2_N+CH_2_O), 3.78 (dd, *J* = 12.2, 8.5 Hz, 1H, CH_2_O), 3.31 (d, *J* = 13.5 Hz, 1H, CH_2_N), 3.03 (ddd, *J* = 8.2, 4.4, 1.5 Hz, 1H, CHN), 2.05–1.99 (m, 2H, 2×CH cyclopropane), 1.88 (t, *J* = 4.6 Hz, 1H, CHCO_2_) ppm. ^13^C-NMR (100 MHz, D_2_O) δ 175.9, 163.0 (q, *J* = 35.5 Hz), 116.3 (q, *J* = 291.7 Hz), 60.6, 59.4, 56.3, 41.6, 26.1, 24.3, 15.8 ppm. IR (NaCl): 3398, 2991, 2884, 1710, cm^−1^. ${\left[\alpha \right]}_{D}^{25}$ (c 0.03 in MeOH): +4.19. Anal. Calc. for C_10_H_14_F_3_NO_5_: C, 42.1; H, 5.0; N, 4.9%. Found: C, 41.9; H, 5.4; N, 5.1%.

*Tert-butyl (1S,4S,5S,6S,7S)-4-(([tert-butyldimethylsilyl]oxy)methyl)-5-hydroxy-7-(hydroxymethyl)-3-azabicyclo[4.1.0]heptane-3-carboxylate*: To a solution of **7** (400 mg, 0.87 mmol) at 0 °C in 3 mL of DCM was added 2.2 mL of DIBAL-H 1.2M in toluene. The reaction was stirred 1 h at room temperature. Methanol was added (5 mL). The salts were filtered and rinsed with methanol (3 × 15 mL). The solvent was evaporated in vacuo. The crude was purified in silica gel in Hex:AcOEt (1:1). A yellow wax was obtained (270 mg, 70%). ^1^H-NMR (400 MHz, CDCl_3_) δ 4.11 (brs, 1H, CHOH), 3.95–3.71 (m, 4H, 2CH_2_O+CHN+CH_2_N), 3.56 (dd, *J* = 11.2, 6.2 Hz, 1H, CH_2_OH), 3.39–3.26 (m, 2H, CH_2_OH+CH_2_N), 1.41 (s, 9H, 3×CH_3_), 1.13–1.05 (m, 1H, CHCHOH), 1.06–0.99 (m, 1H, CHCH_2_N), 0.99–0.90 (m, 1H, CHCH_2_OH), 0.85 (s, 9H, 3×CH_3_), 0.05 (s, 3H, CH_3_), 0.04 (s, 3H, CH_3_) ppm. ^13^C-NMR (100 MHz, CDCl_3_) δ 155.5, 80.2, 67.8, 65.0, 61.7, 54.0, 37.8, 28.5 (3C), 25.8 (3C), 23.3, 20.5, 18.1, 15.1, −5.4 (2C) ppm. IR (NaCl): 3384, 2984, 2896, 1698 cm^−1^. ${\left[\alpha \right]}_{D}^{25}$ (c 0.10 in CHCl_3_): +23.77. Anal. Calc. for C_19_H_37_NO_5_Si: C, 58.9; H, 9.6; N, 3.6%. Found: C, 59.1; H, 9.3; N, 3.5%.

*Tert-butyl (1S,4S,5S,6S,7S)-5-hydroxy-4,7-bis(hydroxymethyl)-3-azabicyclo[4.1.0]heptane-3-carboxylate*: To a solution of tert-butyl (1*S*,4*S*,5*S*,6*S*,7*S*)-4-(([*tert*-butyldimethylsilyl]oxy)methyl)-5-hydroxy-7-(hydroxymethyl)-3-azabicyclo[4.1.0]heptane-3-carboxylate (270 mg, 0.70 mmol) in 7 mL of THF was added TBAF.3H_2_O (243 mg, 0.77 mmol). The reaction was stirred at room temperature for 1 h. The solvent was evaporated in vacuo and purified in silica gel in AcOEt 100%. A yellow wax was obtained (185 mg, 68%). ^1^H-NMR (400 MHz, MeOD) δ 4.05–3.98 (m, 2H, CHN+CH_2_N), 3.95 (dd, *J* = 5.8, 2.6 Hz, 1H, C**H**OH), 3.91–3.81 (m, 1H, NCHC**H**_2_OH), 3.74 (dd, *J* = 11.5, 8.6 Hz, 1H, NCHC**H**_2_OH), 3.47–3.26 (m, 3H, 2CH_2_OH+CH_2_N), 1.45 (s, 9H, 3×CH_3_), 1.06–0.98 (m, 1H, CHCH_2_N), 1.00–0.91 (m, 1H, CHCHOH), 0.75 (tt, *J* = 6.8, 4.6 Hz, 1H, CHCH_2_OH) ppm. ^13^C-NMR (100 MHz, MeOD) δ 157.7, 81.3, 68.0, 65.5, 58.9, 56.5 (major), 55.5 (minor), 38.9 (minor), 37.7 (major), 28.7 (3C), 25.0, 21.6, 16.7 (minor), 16.4 (major) ppm. IR (NaCl): 3413, 2992, 2887, 1702 cm^−1^. ${\left[\alpha \right]}_{D}^{25}$ (c 0.05 in MeOH): +13.02. Anal. Calc. for C_13_H_23_NO_5_: C, 57.1; H, 8.5; N, 5.1%. Found: C, 57.3; H, 8.3; N, 5.0%.

*((1S,4S,5S,6S,7S)-4,7-bis(hydroxymethyl)-3-azabicyclo[4.1.0]heptane-5-ol* (**11a**): To a solution of tert-butyl (1*S*,4*S*,5*S*,6*S*,7*S*)-5-hydroxy-4,7-bis(hydroxymethyl)-3-azabicyclo[4.1.0]heptane-3-carboxylate (185 mg, 0.68 mmol) in 3 mL of DCM and 0.7 mL of MeOH was added TFA (6.8 mL). The reaction was stirred for 20 min. The solvent was evaporated in vacuo. The crude was filtered through a basic DOWEX resin. A brown wax was obtained (107 mg, 91%). ^1^H-NMR (400 MHz, MeOD) δ 4.28 (brs, 1H, CHOH), 3.77–3.74 (m, 2H, NCHCH_2_OH), 3.70 (dd, *J* = 13.7, 8.3 Hz, 1H, CH_2_N), 3.48 (dd, *J* = 11.4, 6.1 Hz, 1H, CH_2_OH), 3.41 (dd, *J* = 11.4, 6.5 Hz, 1H, CH_2_OH), 3.12 (dd, *J* = 13.7, 2.4 Hz, 1H, CH_2_N), 2.86 (t, *J* = 5.9 Hz, 1H, CHN), 1.33-1.23 (m, 2H, 2×CH cyclopropane), 1.14 (p, *J* = 5.6 Hz, 1H, CHCH_2_OH) ppm. ^13^C-NMR (100 MHz, MeOD) δ 64.8, 62.7, 61.2, 58.5, 44.3, 26.7, 23.7, 11.9 ppm. IR (NaCl): 3429, 2988, 2892 cm^−1^. ${\left[\alpha \right]}_{D}^{25}$ (c 0.02 in MeOH): +8.51. Anal. Calc. for C_8_H_15_NO_3_: C, 55.5; H, 8.7; N, 8.1%. Found: C, 55.9; H, 8.2; N, 8.3%.

*(1R,4S,5S,6S,7R)-3-(tert-Butoxycarbonyl)-4-(([tert-butyldimethylsilyl]oxy)methyl)-5-hydroxy-3-azabicyclo[4.1.0]heptane-7-carboxylic acid* (**8**): To a solution of **4b** (260 mg, 0.57 mmol) in 3 mL of absolute ethanol was added NaBH_4_ (43 mg, 1.14 mmol). The reaction was stirred for 48 h. Methanol was added (5 mL). The salts were filtered and rinsed with methanol (3 × 10 mL). The solvent was evaporated in vacuo. The crude was purified in silica gel from Hex:AcOEt (4:1) to Hex:AcOEt (2:1). A yellow wax was obtained as 2 conformers in a ratio 56:44 (70 mg, 30%). ^1^H-NMR (400 MHz, CDCl_3_) δ 5.04 (brs, 2H, CHOH, major + minor), 4.18 (brs, 1H, CHN, minor), 4.13–4.04 (m, 1H, CHN, major), 3.78–3.48(m, 8H, CH_2_O and CH_2_N, major + minor), 2.56–2.47 (m, 2H, CHCHOH, major + minor), 2.22 (dd, *J* = 8.5, 6.2 Hz, 2H, CHCO_2_, major + minor), 1.82–1.70 (m, 2H, CHCH_2_N, major + minor), 1.44 (s, 18H, 3×CH_3_, major + minor), 0.89 (s, 18H, 3×CH_3_, major + minor), 0.07 (s, 6H, CH_3_, major + minor), 0.06 (s, 6H, CH_3_, major + minor) ppm. ^13^C-NMR (100 MHz, CDCl_3_) δ 173.7 (minor), 173.5 (major), 155.0, 80.6, 80.4, 74.1 (minor), 73.4 (major), 61.5 (major), 61.2 (minor), 52.4 (major), 51.6 (minor), 36.9 (minor), 35.9 (major), 28.4 (3C), 25.8 (3C), 23.9 (minor), 23.8 (major), 20.4 (minor), 19.8 (major), 18.1 (major), 16.3 (minor), −5.4, −5.5 ppm. IR (NaCl): 3408, 2995, 2891, 1712, 1698 cm^−1^. ${\left[\alpha \right]}_{D}^{25}$ (c 0.02 in MeOH): +25.10. Anal. Calc. for C_19_H_35_NO_6_Si: C, 56.8; H, 8.8; N, 3.5%. Found: C, 56.4; H, 8.5; N, 3.7%.

*(1R,4S,5S,6S,7R)-3-(tert-butoxycarbonyl)-5-hydroxy-4-(hydroxymethyl)-3-azabicyclo[4.1.0]heptane-7-carboxylic acid*: To a solution of **8** (70 mg, 0.17 mmol) in 1 mL of THF was added TBAF.3H_2_O (59 mg, 0.19 mmol). The reaction was stirred for 30 min. The solvent was evaporated in vacuo. The crude was purified in silica gel in Hex:AcOEt (1:4). A yellow wax was obtained (48 mg, 88%). ^1^H-NMR (400 MHz, CDCl_3_) δ 5.03 (brs, 1H, CHOH), 4.24–4.15 (m, 1H, CHN), 3.84–3.74 (m, 2H, CH_2_O+CH_2_N), 3.73–3.63 (m, 1H, CH_2_O), 3.50 (d, *J* = 14.5 Hz, 1H, CH_2_N), 2.60–2.50 (m, 1H, CHCHOH), 2.23 (dd, *J* = 8.5, 6.2 Hz, 1H, CHCO_2_), 1.78 (qd, *J* = 8.0, 2.5 Hz, 1H, CHCH_2_N), 1.42 (s, 9H, 3×CH_3_) ppm.^13^C-NMR (100 MHz, CDCl_3_) δ 173.6, 156.1, 81.1, 73.3, 61.3, 52.6, 36.6, 28.3 (3C), 23.9, 19.9, 16.2 ppm. IR (NaCl): 3419, 2992, 2901, 1701 cm^−1^. ${\left[\alpha \right]}_{D}^{25}$ (c 0.01 in MeOH): +15.41. Anal. Calc. for C_13_H_21_NO_6_: C, 54.4; H, 7.4; N, 4.9%. Found: C, 54.6; H, 7.1; N, 5.1%.

*(1R,4S,5S,6S,7R)-7-carboxy-5-hydroxy-4-(hydroxymethyl)-3-azabicyclo[4.1.0]heptan-3-ium trifluoroacetate* (**10b**): To a solution of (1*R*,4*S*,5*S*,6*S*,7*R*)-3-(*tert*-butoxycarbonyl)-5-hydroxy-4-(hydroxymethyl)-3-azabicyclo[4.1.0]heptane-7-carboxylic acid (48 mg, 0.17 mmol) in a mixture of 1 mL of DCM and 0.5 mL of MeOH was added TFA (1.7 mL). The reaction was stirred for 20 min. The solvent was evaporated in vacuo. A brown wax was obtained (44 mg, 92%). ^1^H-NMR (400 MHz, D_2_O) δ 5.19 (d, *J* = 5.5 Hz, 1H, CHOH), 4.00 (dd, *J* = 14.7, 8.7 Hz, 1H, CH_2_N), 3.93 (dd, *J* = 10.7, 5.4 Hz, 2H, CH**_2_**O), 3.71 (dd, *J* = 6.3, 4.5 Hz, 1H, CHN), 2.86–2.74 (m, 2H, CH_2_N+CHCHOH), 2.65 (dd, *J* = 8.4, 5.9 Hz, 1H, CHCO_2_), 2.30 (qd, *J* = 8.3, 6.4 Hz, 1H, C**H**CH_2_N) ppm.^13^C-NMR (100 MHz, D_2_O) δ 175.2, 163.0 (q, *J* = 35.5 Hz), 116.4 (q, *J* = 291.9 Hz), 73.6, 58.9, 54.3, 36.9, 24.7, 18.4, 14.3 ppm. IR (NaCl): 3385, 2997, 2899, 1707, cm^−1^. ${\left[\alpha \right]}_{D}^{25}$ (c 0.01 in MeOH): +10.19. Anal. Calc. for C_10_H_14_F_3_NO_5_: C, 42.1; H, 5.0; N, 4.9%. Found: C, 41.8; H, 5.2; N, 5.0%.

*Tert-butyl (1S,4S,5S,6S,7R)-4-(([tert-butyldimethylsilyl]oxy)methyl)-5-hydroxy-7-(hydroxymethyl)-3-azabicyclo[4.1.0]heptane-3-carboxylate* (**9**): To a solution of **4b** (260 mg, 0.57 mmol) in 3 mL of absolute ethanol was added NaBH_4_ (43 mg, 1.14 mmol). The reaction was stirred for 48 h. Methanol was added (5 mL). The salts were filtered and rinsed with methanol (3 × 10 mL). The solvent was evaporated in vacuo. The crude was purified in silica gel from Hex:AcOEt (4:1) to Hex:AcOEt (2:1). A yellow wax was obtained (110 mg, 50%). ^1^H-NMR (400 MHz, CDCl_3_) δ 4.35 (dd, *J* = 8.8, 1.4 Hz, 1H, C**H**OH), 4.11–4.03 (m, 1H, CHN), 3.93 (d, *J* = 14.2 Hz, 1H, CH_2_N), 3.86 (dd, *J* = 11.5, 5.7 Hz, 1H, CH_2_OH), 3.59 (dd, *J* = 10.1, 7.5 Hz, 1H, CH_2_O), 3.56–3.43 (m, 2H, CH_2_O+CH_2_OH), 3.12 (dd, *J* = 14.3, 5.3 Hz, 1H, CH_2_N), 2.87 (brs, 1H, OH), 1.67 (brs, 1H, OH), 1.43 (q, *J* = 8.9 Hz, 1H, CHCHOH), 1.38 (s, 9H, 3×CH_3_), 1.32–1.23 (m, 1H, CHCH_2_OH), 1.13–1.02 (m, 1H, CHCH_2_N), 0.82 (s, 9H, 3×CH_3_), 0.000 (s, 3H, CH_3_), −0.004 (s, 3H, CH_3_) ppm. ^13^C-NMR (100 MHz, CDCl_3_) δ 155.6, 80.3, 64.3, 61.6, 58.8, 57.4, 35.2, 28.6 (3C), 26.0 (3C), 21.6, 18.3, 15.3, 12.0, −5.30, −5.32 ppm. IR (NaCl): 3372, 2991, 2906, 1706 cm^−1^. ${\left[\alpha \right]}_{D}^{25}$ (c 0.03 in CHCl_3_): +24.04. Anal. Calc. for C_19_H_37_NO_5_Si: C, 58.9; H, 9.6; N, 3.6%. Found: C, 59.2; H, 9.2; N, 3.4%.

*Tert-butyl (1S,4S,5S,6S,7R)-5-hydroxy-4,7-bis(hydroxymethyl)-3-azabicyclo[4.1.0]heptane-3-carboxylate*: To a solution of **9** (110 mg, 0.29 mmol) in 2 mL of THF was added TBAF.3H_2_O (101 mg, 0.32 mmol). The reaction was stirred for 30 min. The solvent was evaporated in vacuo. The crude was purified in silica gel in AcOEt. A yellow wax was obtained (68 mg, 86%). ^1^H-NMR (400 MHz, MeOD) δ 4.29 (d, *J* = 8.9 Hz, 1H, CHOH), 4.14 (t, *J* = 7.6 Hz, 1H, NCHCH_2_OH), 4.03 (d, *J* = 14.1 Hz, 1H, CH_2_N), 3.78 (dd, *J* = 11.7, 7.3 Hz, 1H, NCHCH_2_OH), 3.73–3.65 (m, 1H, CHN), 3.61 (dd, *J* = 11.3, 7.1 Hz, 1H, C**H**_2_OH), 3.52 (dd, *J* = 11.3, 8.3 Hz, 1H, CH_2_OH), 3.28–3.16 (m, 1H, CH_2_N), 1.51 (q, *J* = 9.1 Hz, 1H, CHCHOH), 1.46 (s, 9H, 3×CH_3_), 1.33–1.11 (m, 2H, 2×CH cyclopropane) ppm. ^13^C-NMR (100 MHz, MeOD) δ 157.6, 81.4, 64.4, 61.0, 59.6, 59.2 (major), 57.9(minor), 36.3 (minor), 35.1 (major), 28.7 (3C), 22.3, 16.2, 12.4 ppm. IR (NaCl): 3385, 2988, 2892, 1698 cm^−1^. ${\left[\alpha \right]}_{D}^{25}$ (c 0.02 in MeOH): +10.68. Anal. Calc. for C_13_H_23_NO_5_: C, 57.1; H, 8.5; N, 5.1%. Found: C, 56.8; H, 8.3; N, 5.3%.

*((1S,4S,5S,6S,7R)-4,7-bis(hydroxymethyl)-3-azabicyclo[4.1.0]heptane-5-ol* (**11b**): To a solution of tert-butyl (1S,4S,5S,6S,7R)-5-hydroxy-4,7-bis(hydroxymethyl)-3-azabicyclo[4.1.0]heptane-3-carboxylate (68 mg, 0.25mmol) in 1.5 mL of DCM and 0.7 mL of MeOH was added TFA (2.5 mL). The reaction was stirred for 20 min. The solvent was evaporated in vacuo. The crude was filtered through a basic DOWEX resin. A brown wax was obtained (40 mg, 93%). ^1^H-NMR (400 MHz, MeOD) δ 4.34 (dd, *J* = 10.1, 7.3 Hz, 1H, CHOH), 3.94 (dd, *J* = 11.9, 3.0 Hz, 1H, NCHCH_2_OH), 3.85 (d, *J* = 8.3 Hz, 2H, CH_2_OH), 3.72 (dd, *J* = 12.0, 5.1 Hz, 1H, NCHCH_2_OH), 3.65 (dd, *J* = 14.3, 9.6 Hz, 1H, CH_2_N), 2.94 (dd, *J* = 14.2, 5.0 Hz, 1H, CH_2_N), 2.57–2.48 (m, 1H, CHN), 1.80–1.67 (m, 1H, CHCH_2_N), 1.60 (q, *J* = 8.9 Hz, 1H, CHCHOH), 1.46 (p, *J* = 8.4 Hz, 1H, CHCH_2_OH) ppm. ^13^C-NMR (100 MHz, MeOD) δ 63.7, 61.7, 60.4, 58.6, 40.1, 24.7, 18.9, 15.6 ppm. IR (NaCl): 3441, 2995, 2899 cm^−1^. ${\left[\alpha \right]}_{D}^{25}$ (c 0.01 in MeOH): +6.38. Anal. Calc. for C_8_H_15_NO_3_: C, 55.5; H, 8.7; N, 8.1%. Found: C, 55.3; H, 8.9; N, 8.3%.

## 4. Conclusions

We described the synthesis of bicyclic piperidine-based iminosugars from natural amino acids l-alanine and l-serine. The procedure involves the preparation of enantiomerically pure α,β-unsaturated ketones in four steps and high yields from the natural amino acids. These intermediates, upon a stereoselective cyclopropanation reaction and further straightforward transformations, give the final products, which contain five stereogenic centers. The synthetic methodology used allows the obtention of different configurations at some of the asymmetric carbons, which, in this project, is interesting, because selectivity towards different enzymes could be achieved. The behavior of the products against different glycosidases showed that inhibition was generally low but selective towards one or two enzymes. The activation of the target enzymes was observed in some cases.

## Figures and Tables

**Table 1:** Residual activity of enzymes at 5 mM of the active final compounds.

	α-Glc	β-Glc	α-Gal	β-Gal	α-Man	β-Man	α-l-Fuc
**6a**	*	*	*	*	*	*	*
**6b**	*	*	*	*	*	148%	142%
**6c**	*	43%	*	*	*	*	*
**10a**	*	*	154%	*	*	155%	*
**10b**	*	39%	*	*	*	*	*
**11a**	*	*	*	*	*	*	*
**11b**	*	*	*	25%	*	*	*

* Activity not detected even at 25 mM.

## Data Availability

The data presented in this study are available in this article.

## Supplementary Materials

The following are available online, 1D and 2D NMR spectra of all new compounds (Figures S1–S72). Figure S41: Transglycosidation monitorization by ^1^H-NMR). Table S1: Measured constant coupling from products derived from l-serine.

## Sample Availability

Not available.
