# Supplementary material for: Synthesis and Evaluation of Novel Iminosugars Prepared from Natural Amino Acids
Source: Molecules. 2021 Jan 13;26(2):394. doi: 10.3390/molecules26020394 (PMC7828477; doi:10.3390/molecules26020394)
Supplement: Supplementary file 1 [file molecules-26-00394-s001.pdf]

Supporting Information for Publication

Synthesis and evaluation of novel iminosugars prepared from natural amino acids

Alejandro Puet,[1] Gema Domínguez,[1] F. Javier Cañada[2] and Javier Pérez-Castells[1*]

Figure S1: ^{1}H-NMR (400 MHz, $CDCl_3$) of **1a.**

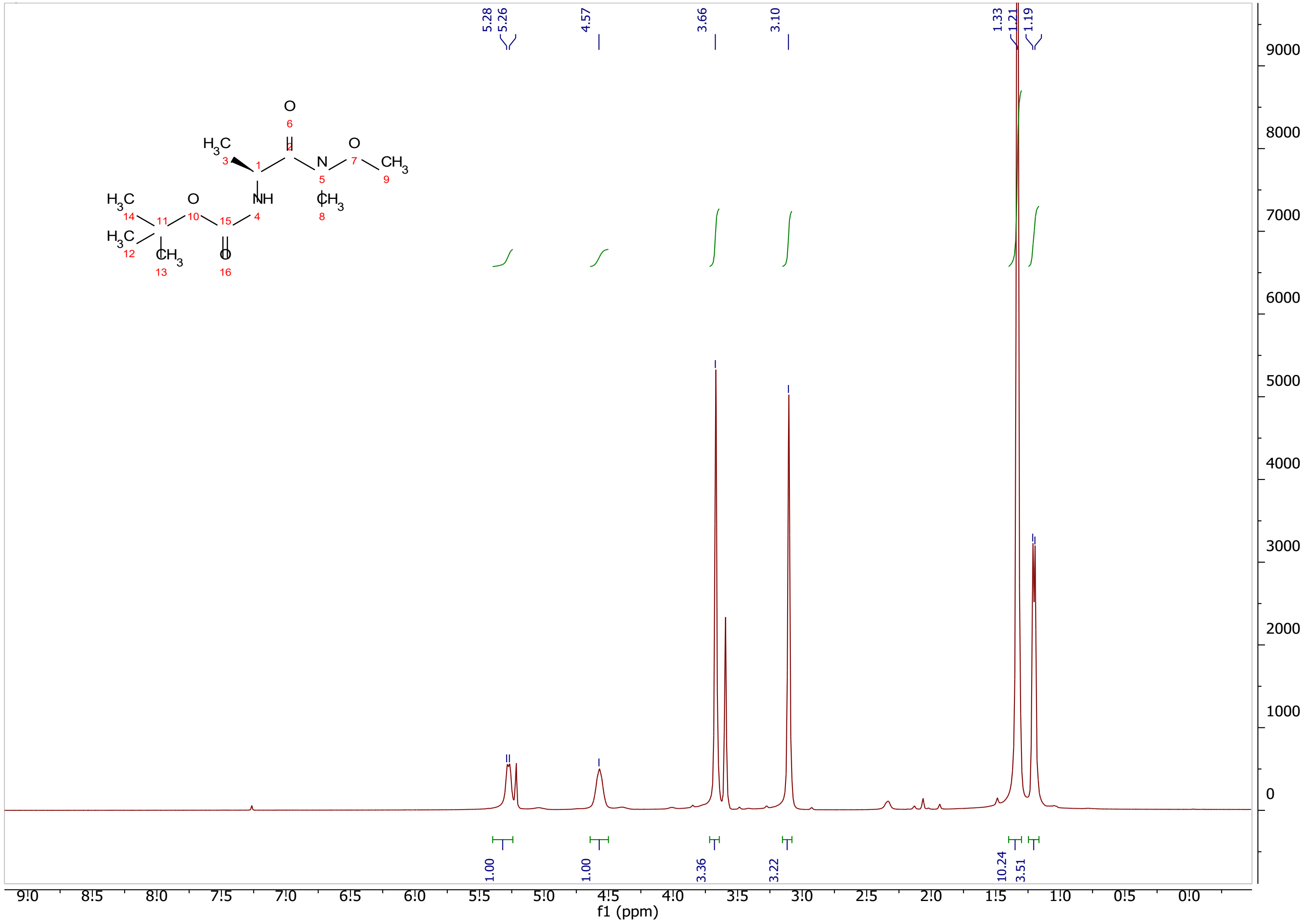

Figure S2: ^{13}C-NMR (100 MHz, $CDCl_3$) of **1a**.

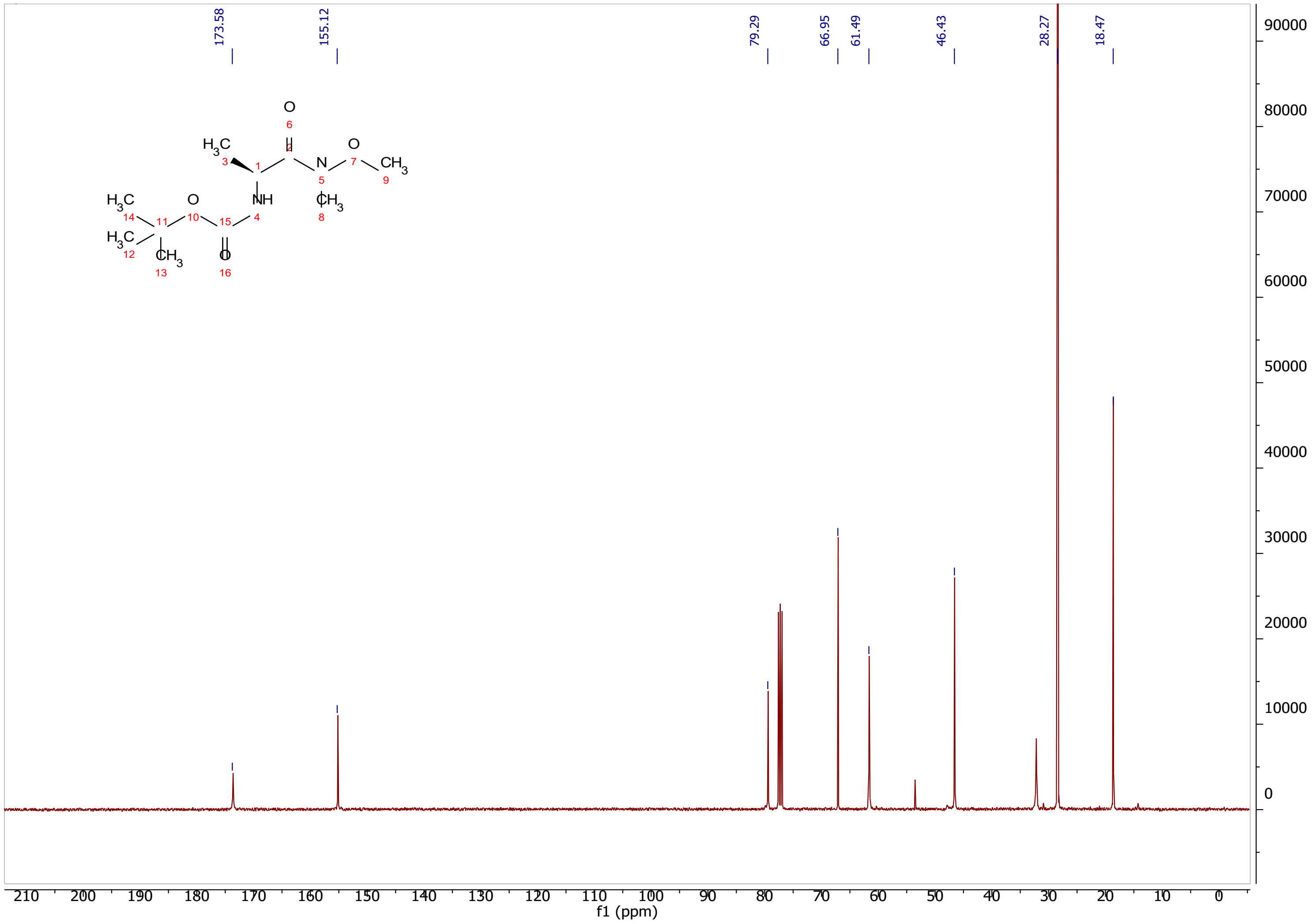

Figure S3: ^{1}H-NMR (400 MHz, $CDCl_3$) of ***tert*-butyl (*S*)-allyl(1-(methoxy(methyl)amino)-1-oxopropan-2-yl)carbamate.**

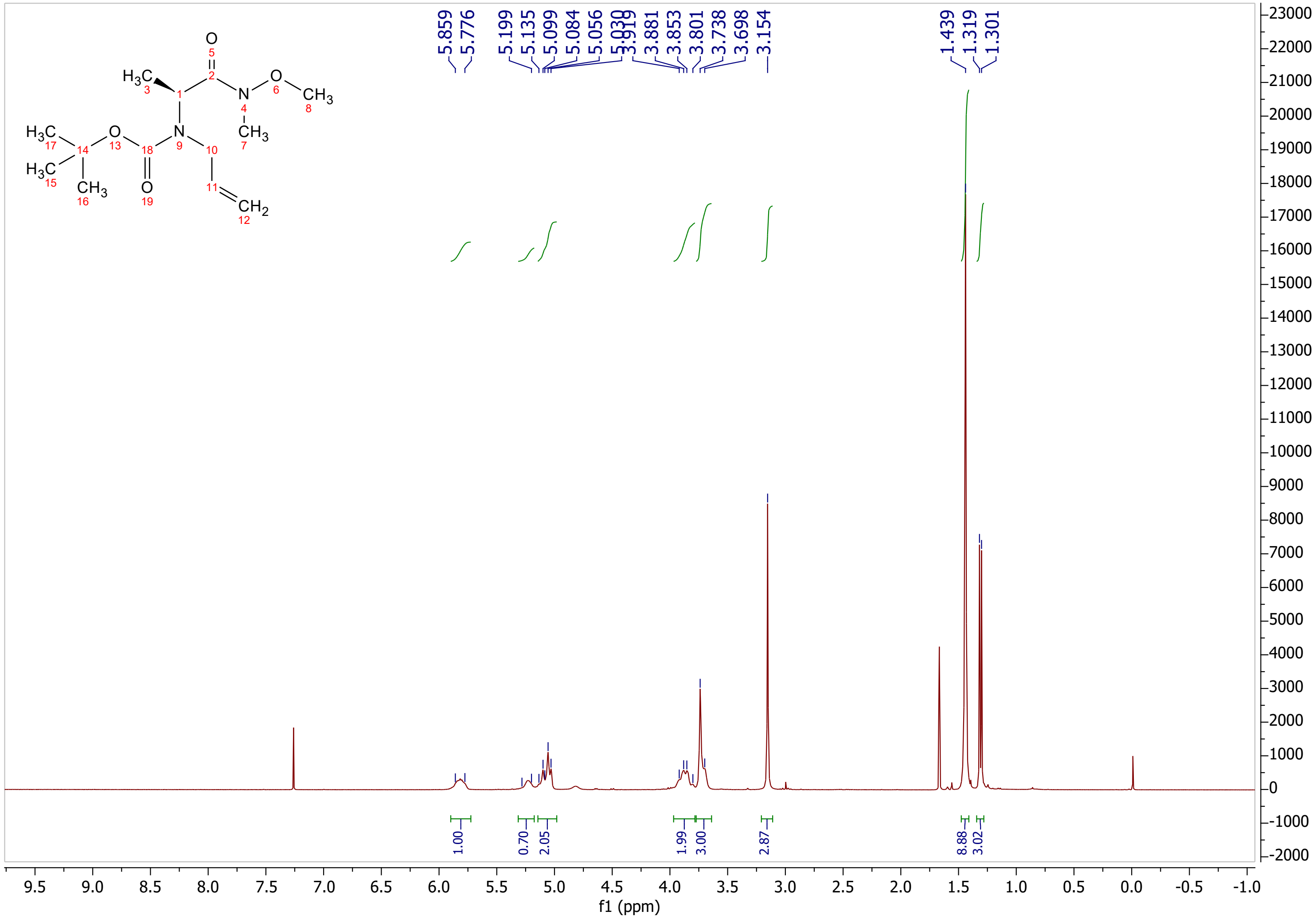

Figure S4: ^{13}C-NMR (100 MHz, $CDCl_3$) of ***tert*-butyl (*S*)-allyl(1-(methoxy(methyl)amino)-1-oxopropan-2-yl)carbamate**.

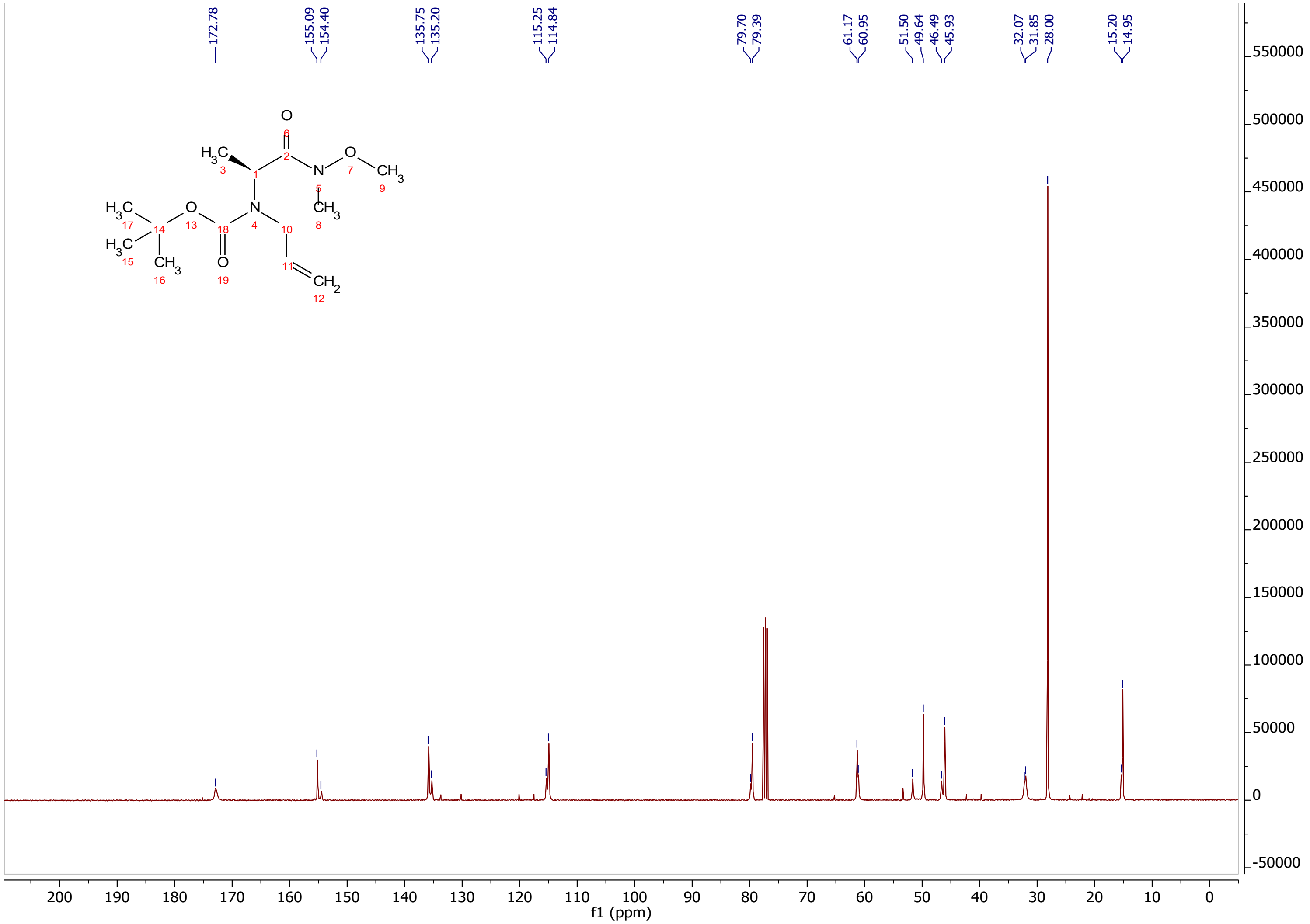

Figure S5: ^{1}H-NMR (400 MHz, $CDCl_3$) of **2a.**

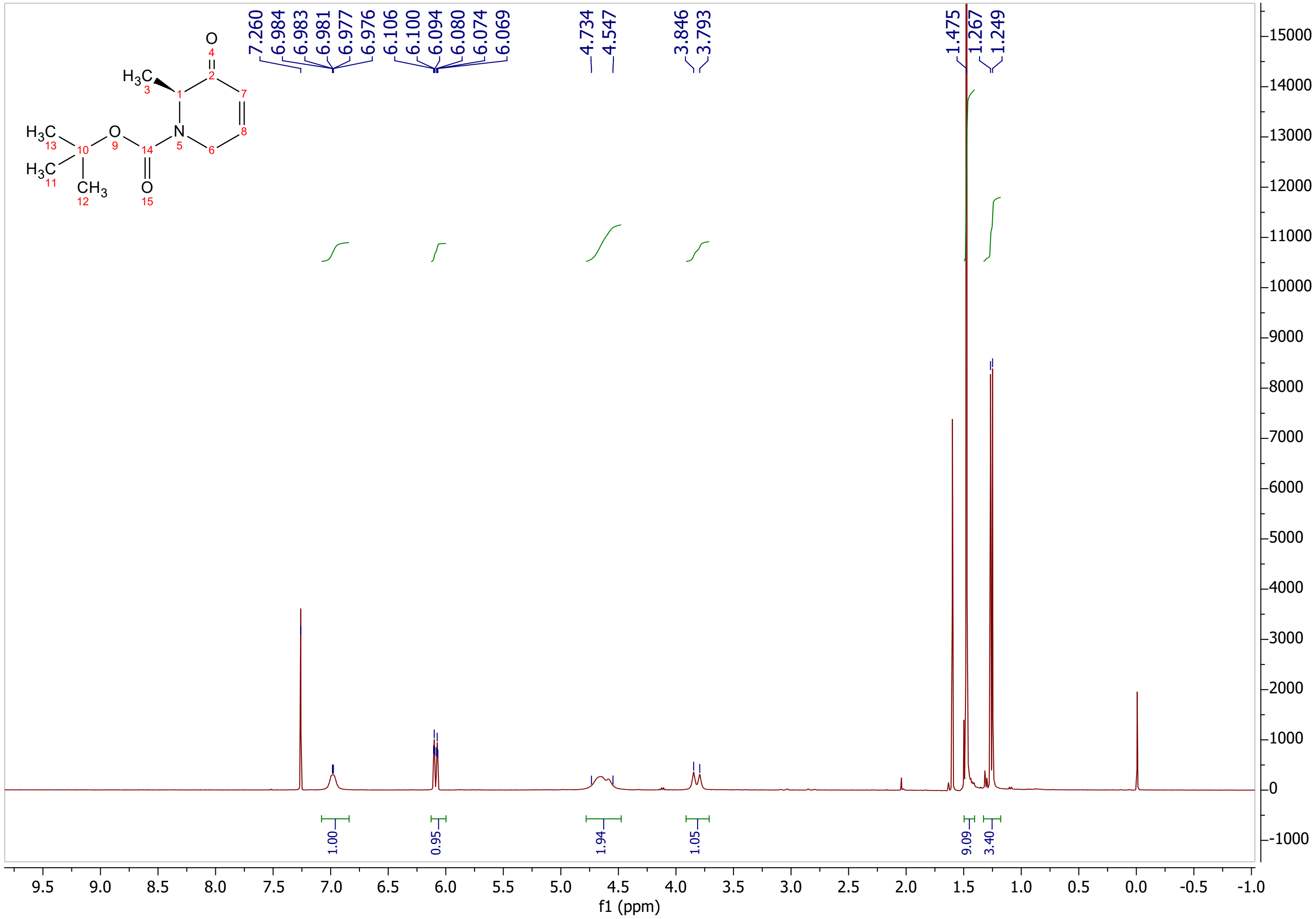

Figure S6: ^{13}C-NMR (100 MHz, $CDCl_3$) of **2a**.

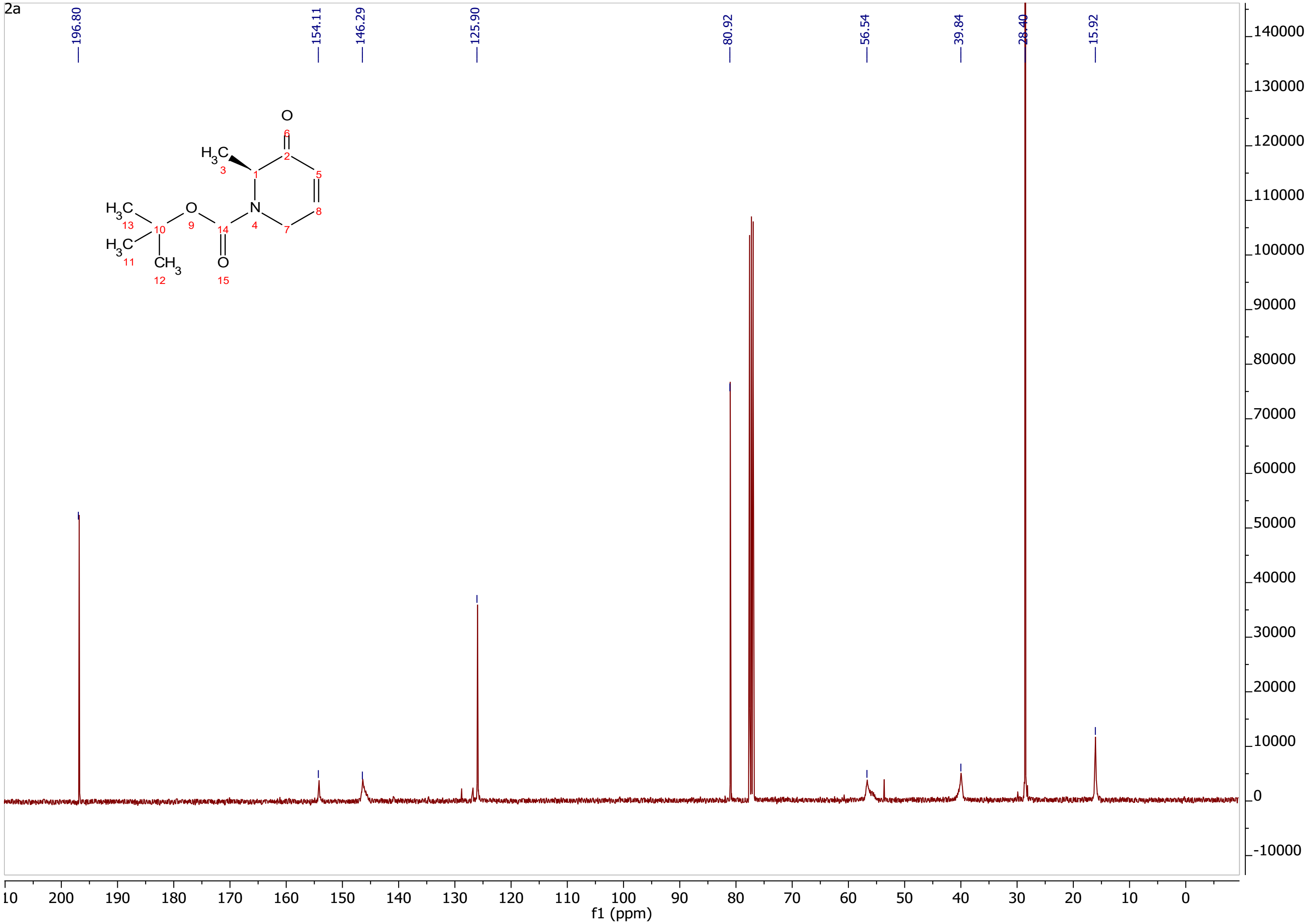

Figure S7: ^{1}H-NMR (400 MHz, CDCl3) of **3a.**

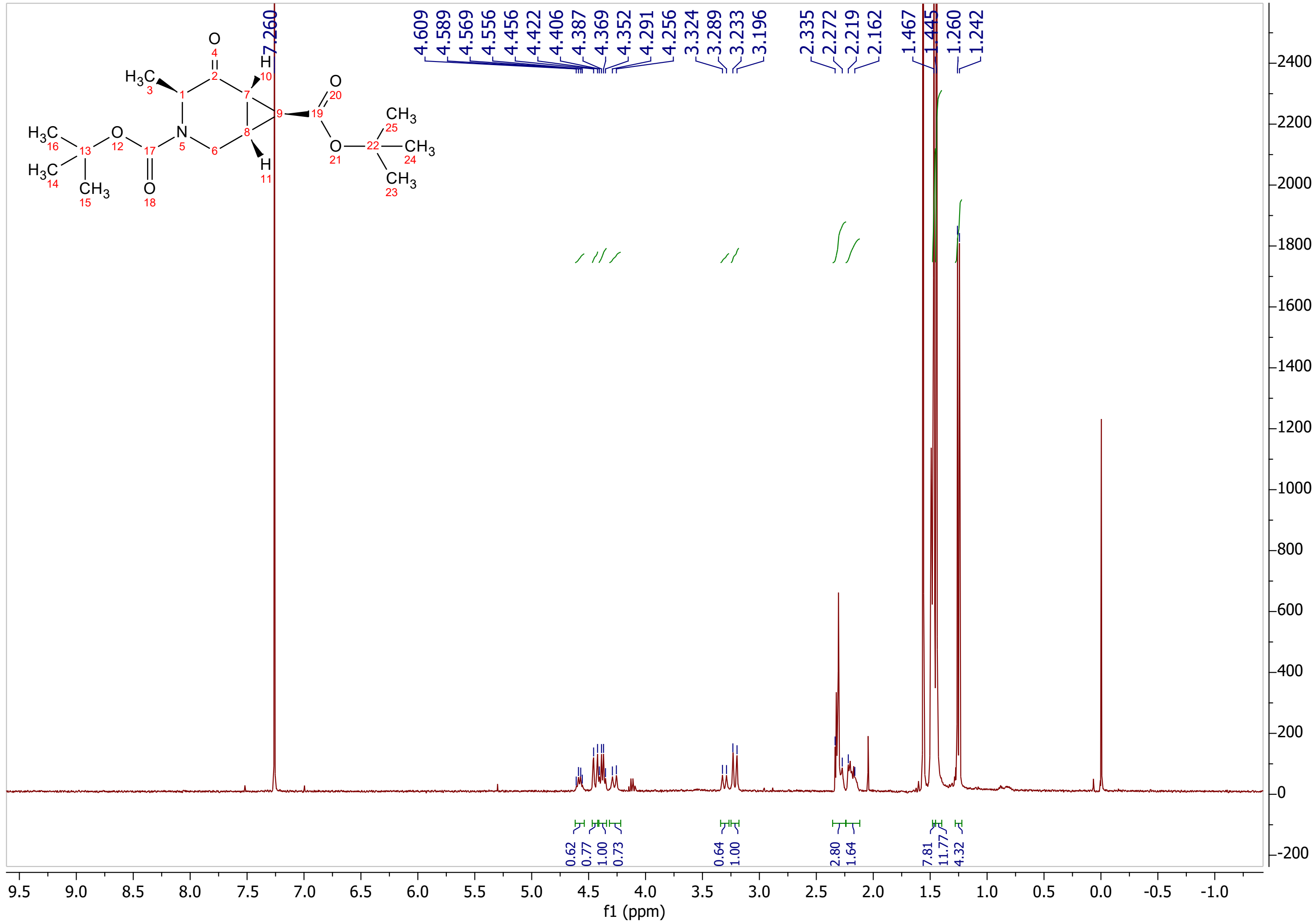

Figure S8: ^{13}C-NMR (100 MHz, $CDCl_3$) of **3a**.

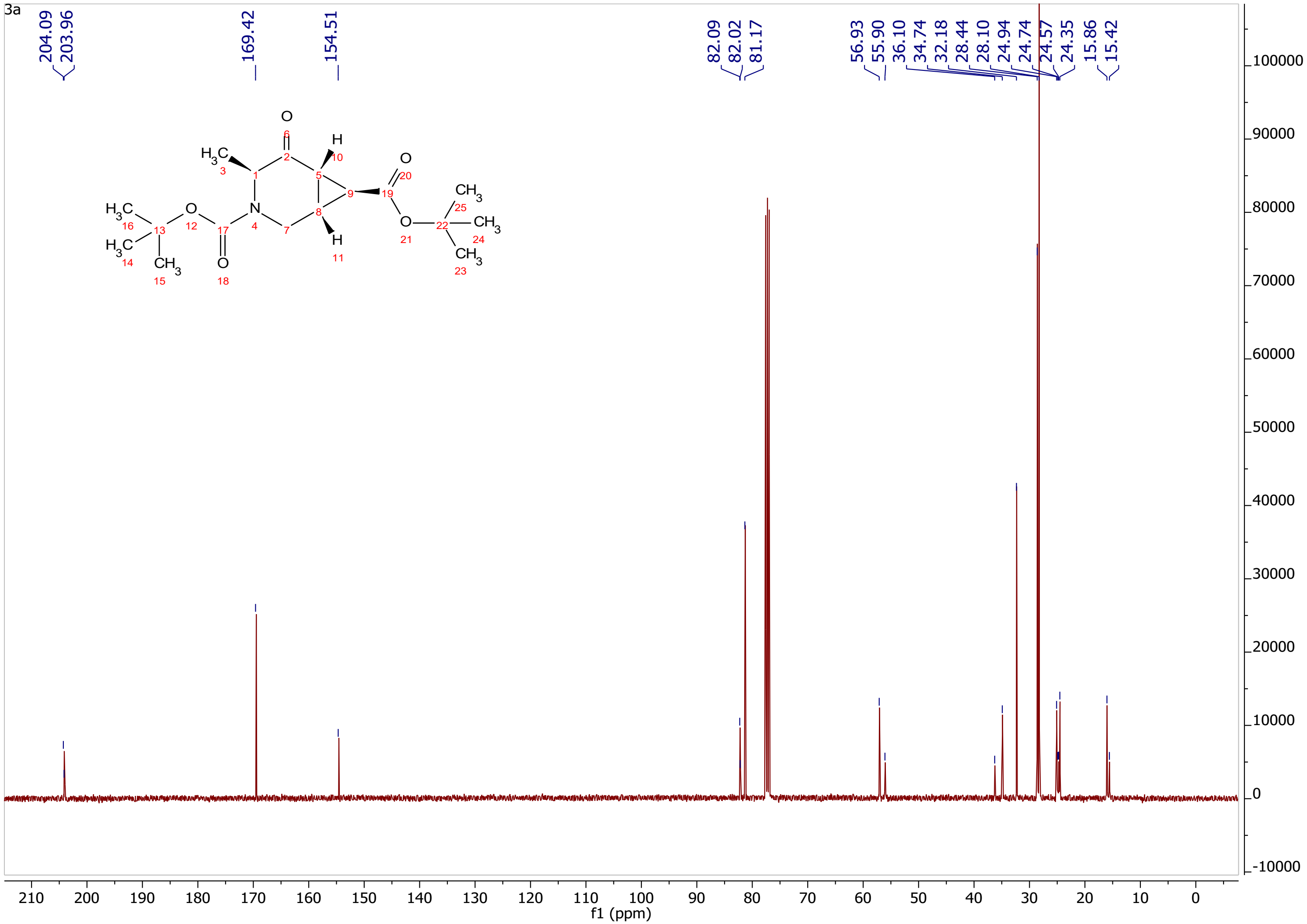

Figure S9: HMQC (400 MHz, $CDCl_3$) of **3a**.

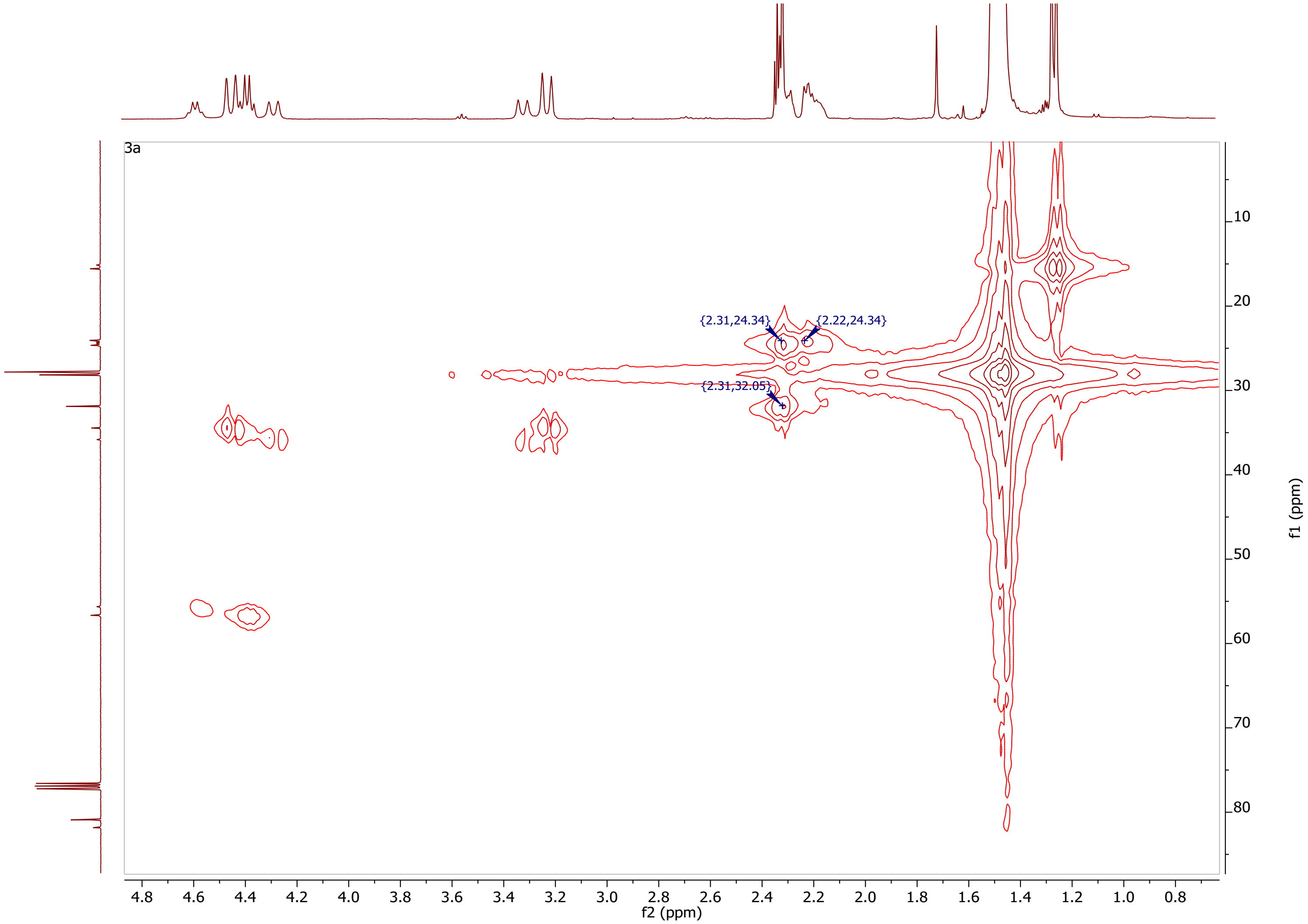

Figure S10: ^{1}H-NMR (400 MHz, $CDCl_3$) of **4a.**

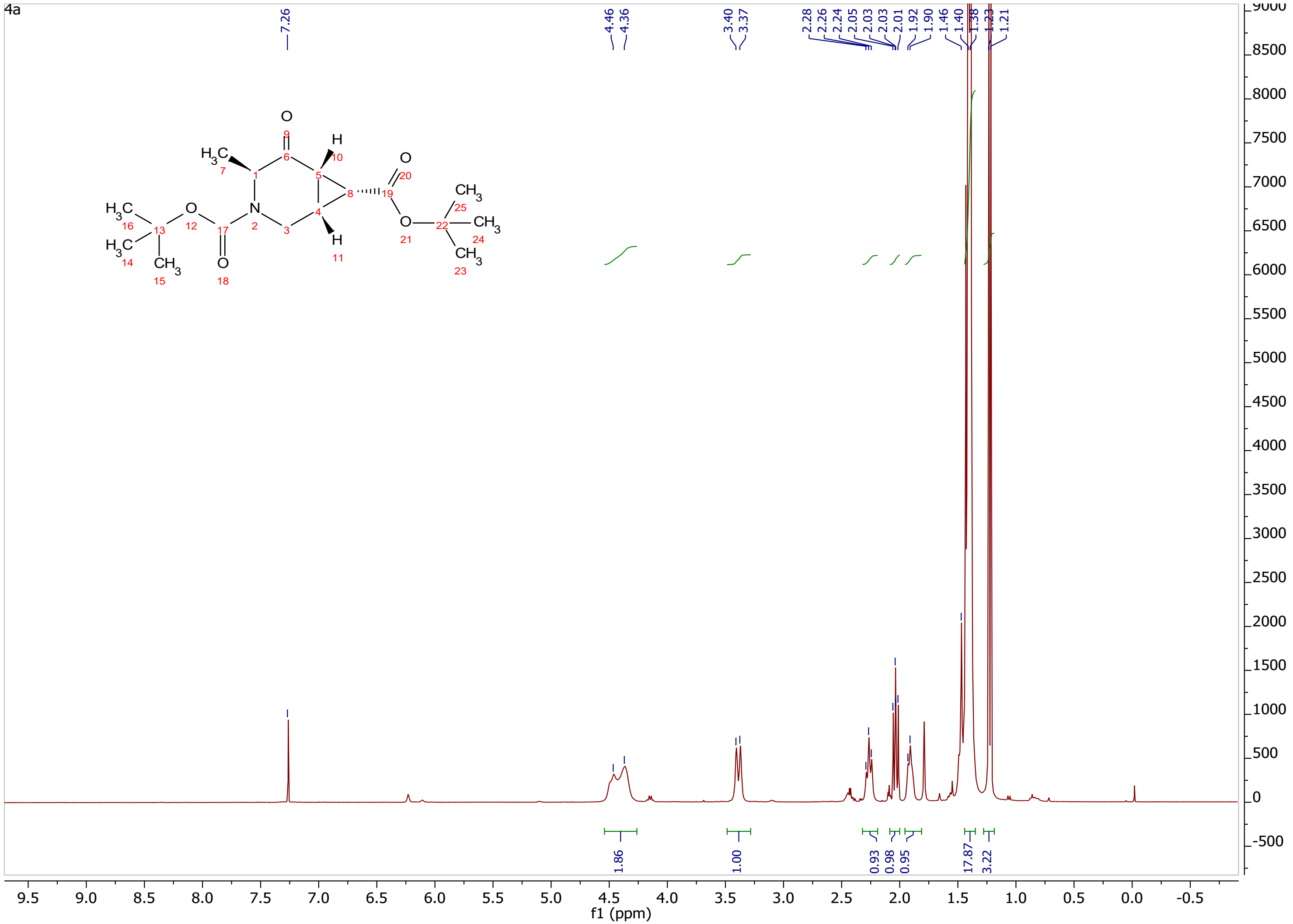

Figure S11: ^{13}C-NMR (100 MHz, $CDCl_3$) of **4a**.

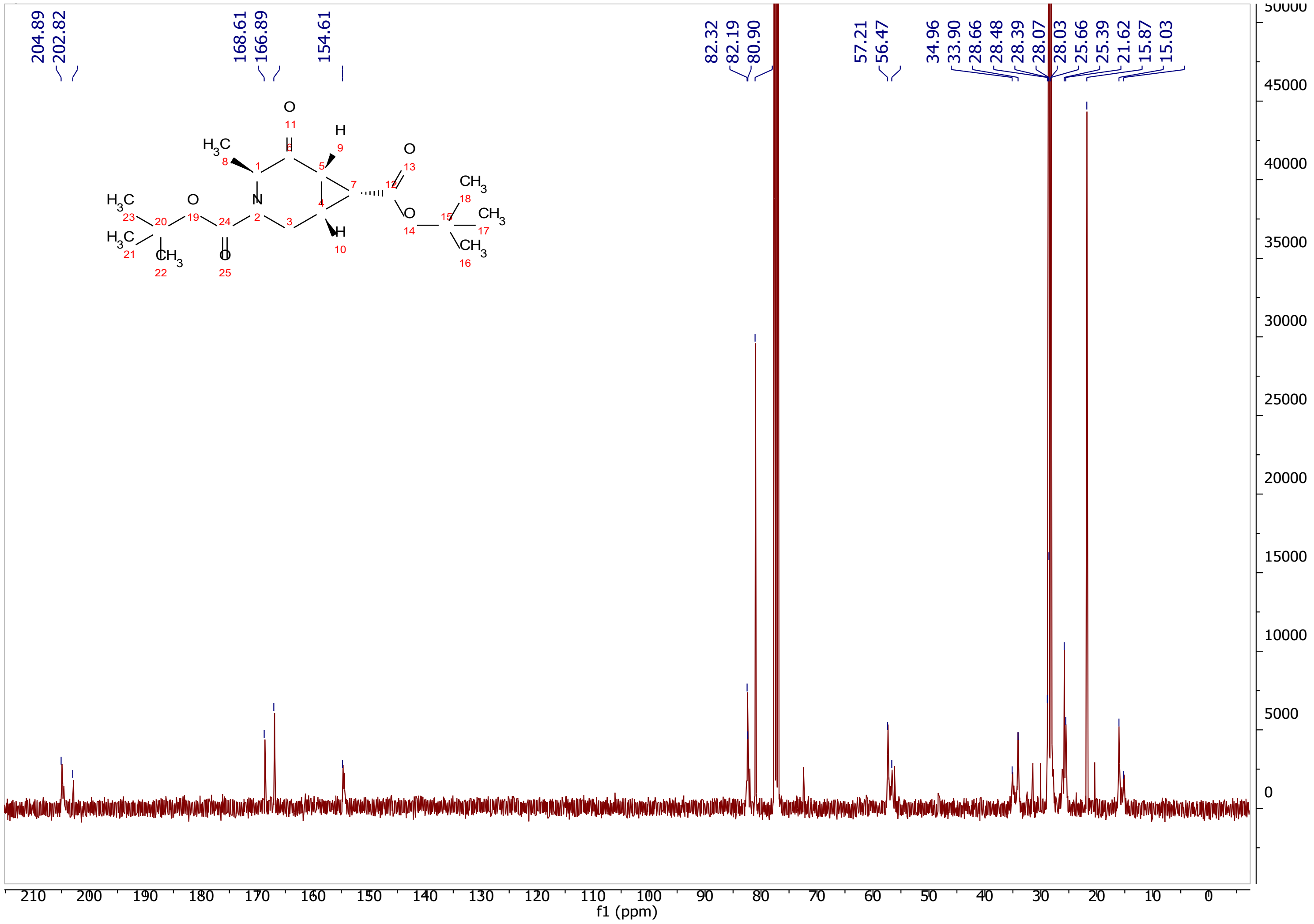

Figure S12: ^{1}H-NMR (400 MHz, $CDCl_3$) of ***tert*-butyl (*S*)-(3-hydroxy-1-(methoxy(methyl)amino)-1-oxopropan-2-yl)carbamate.**

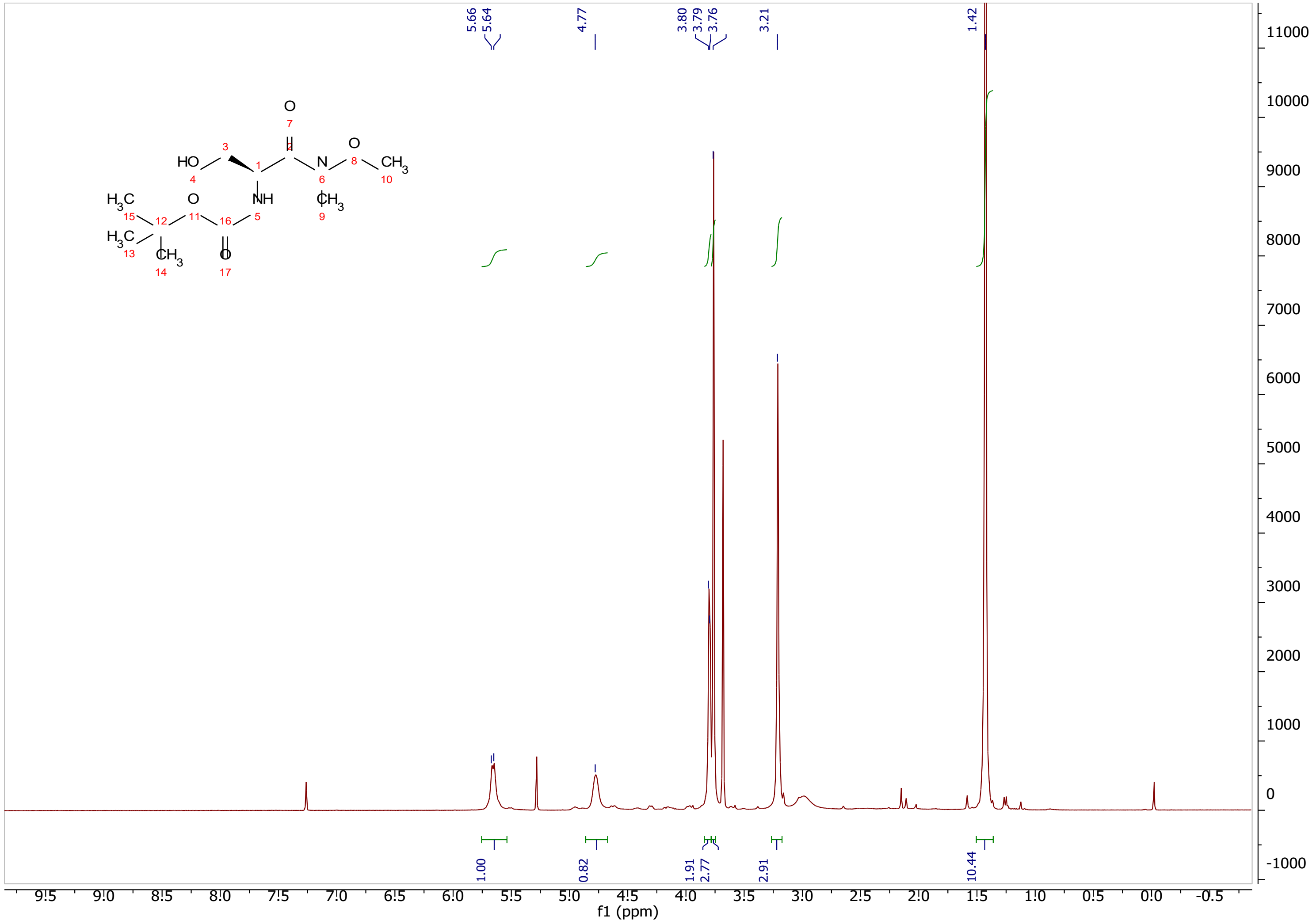

Figure S13: ^{13}C-NMR (100 MHz, $CDCl_3$) of ***tert*-butyl (*S*)-(3-hydroxy-1-(methoxy(methyl)amino)-1-oxopropan-2-yl)carbamate**.

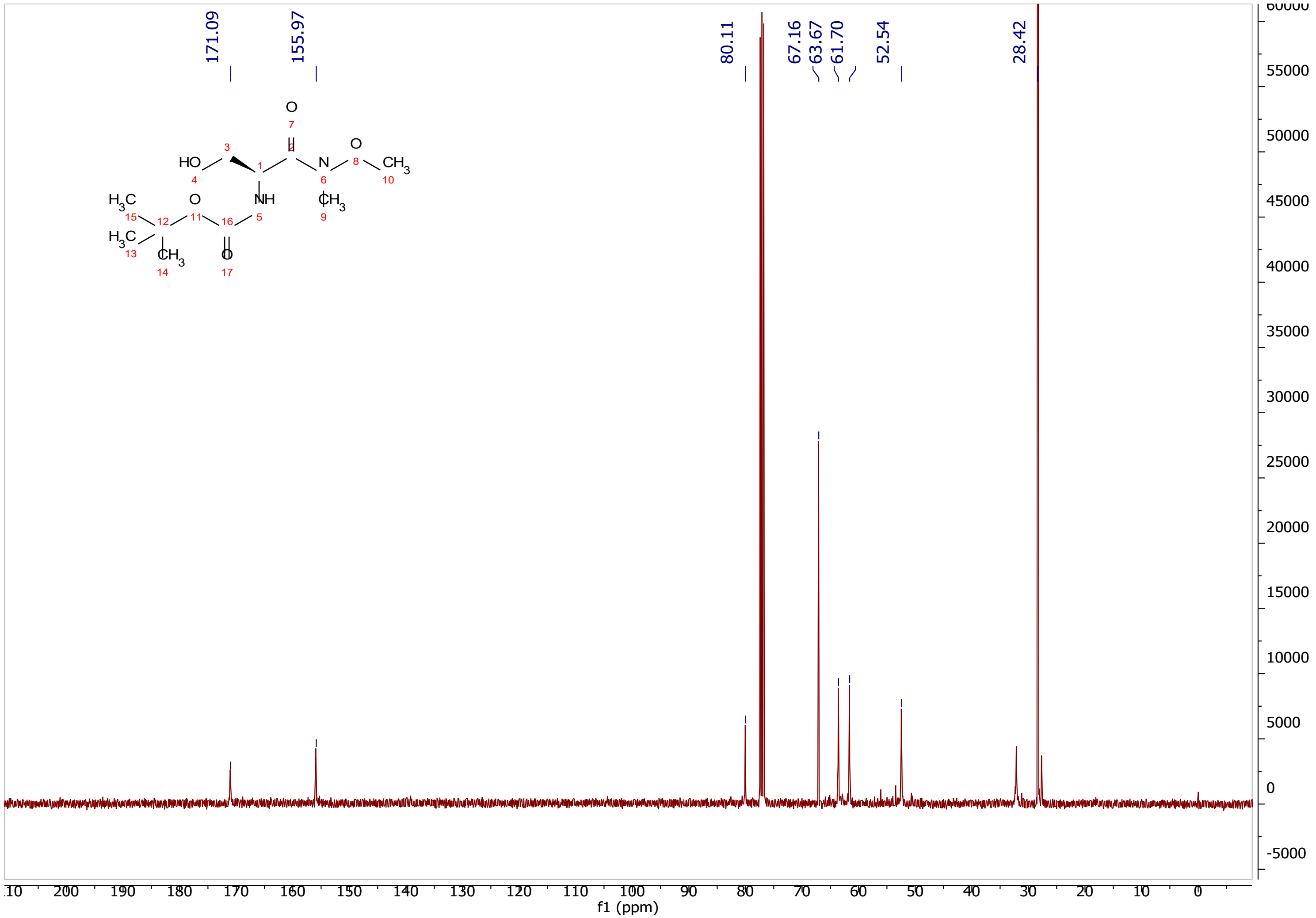

Figure S14: ^{1}H-NMR (400 MHz, $CDCl_3$) of **1b.**

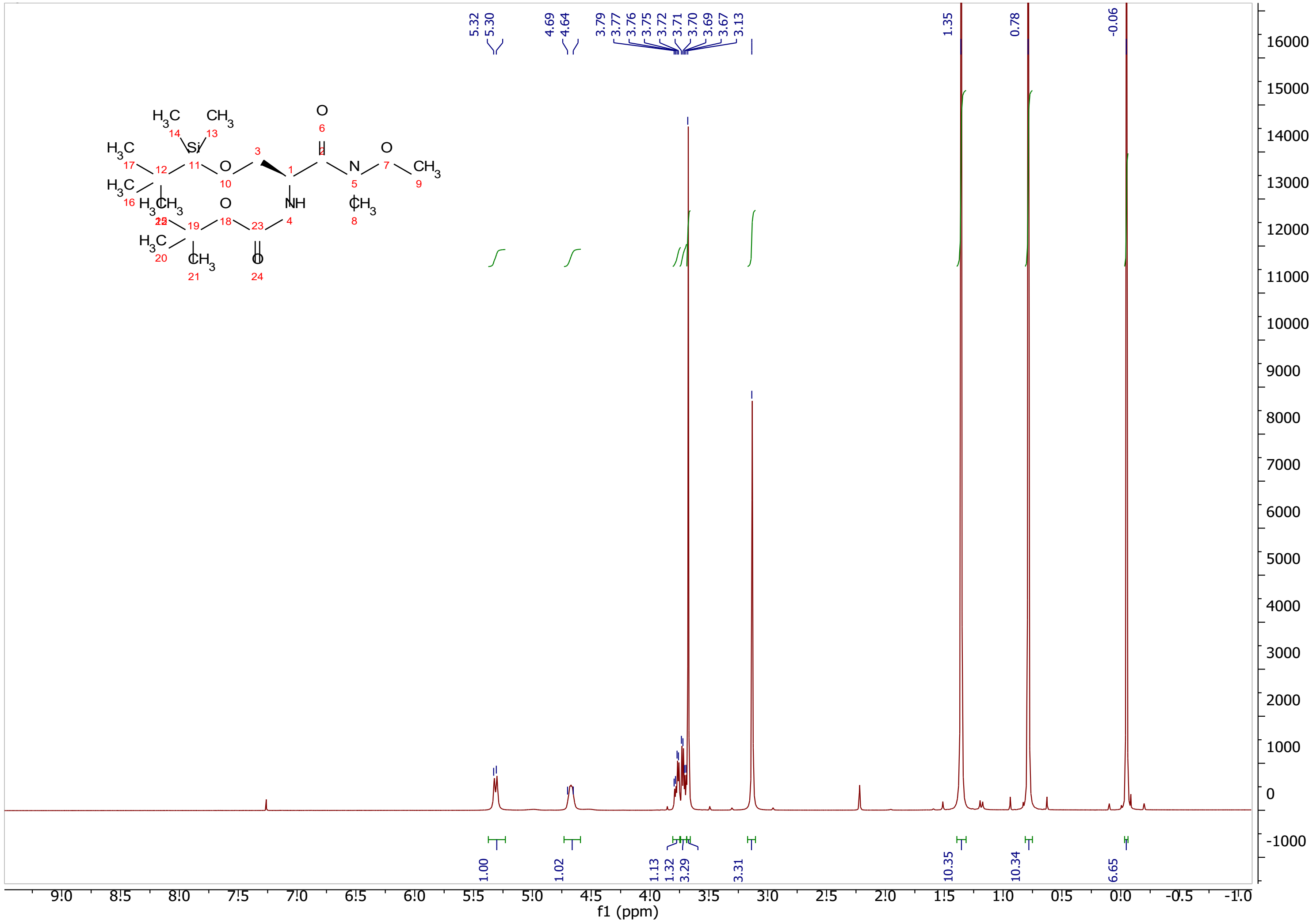

Figure S15: ^{13}C-NMR (100 MHz, $CDCl_3$) of **1b**.

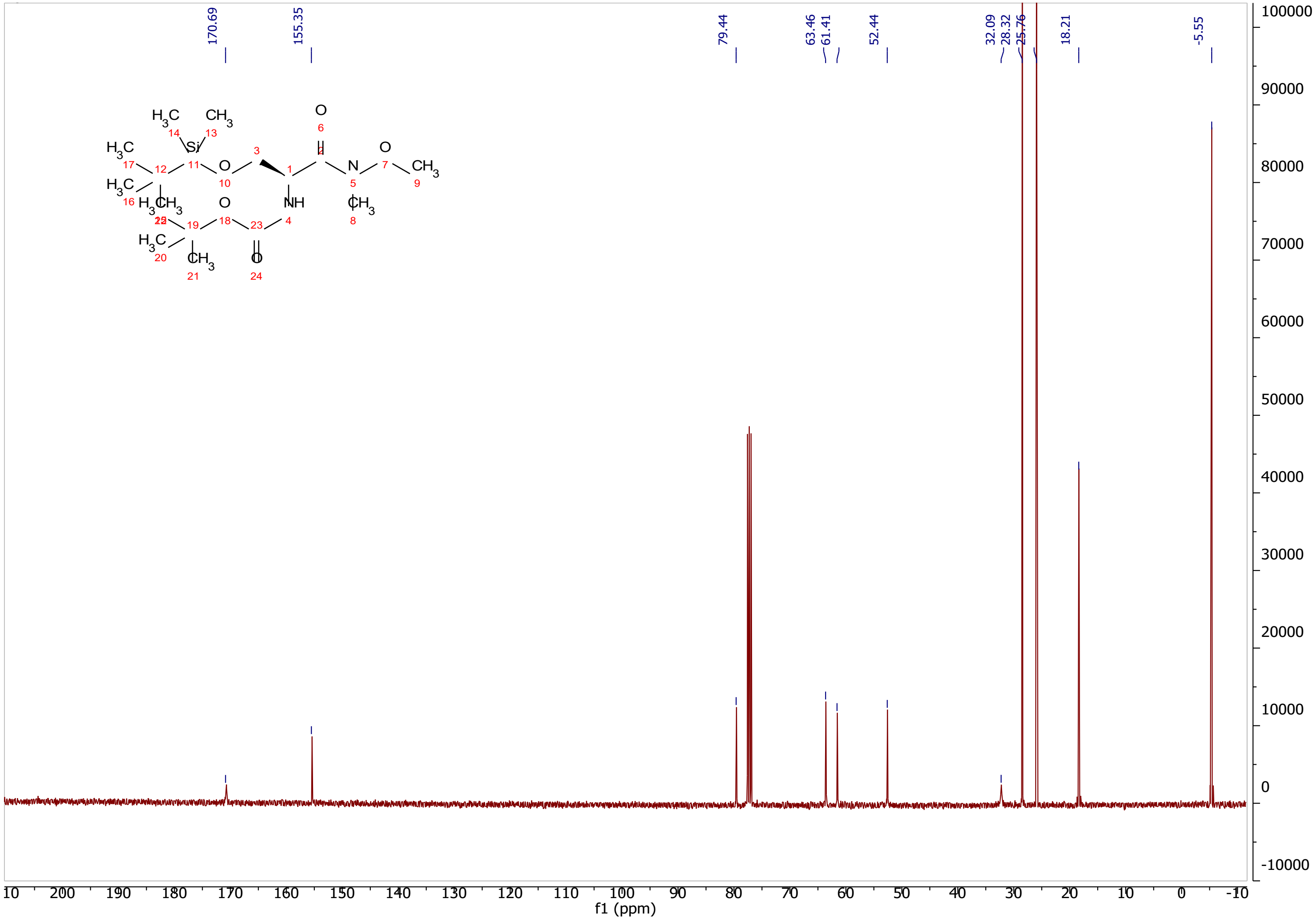

Figure S16: ^{1}H-NMR (300 MHz, $CDCl_3$) of ***tert*-butyl (*S*)-allyl(3,8,8,9,9-pentamethyl-4-oxo-2,7-dioxa-3-aza-8-siladecan-5-yl)carbamate.**

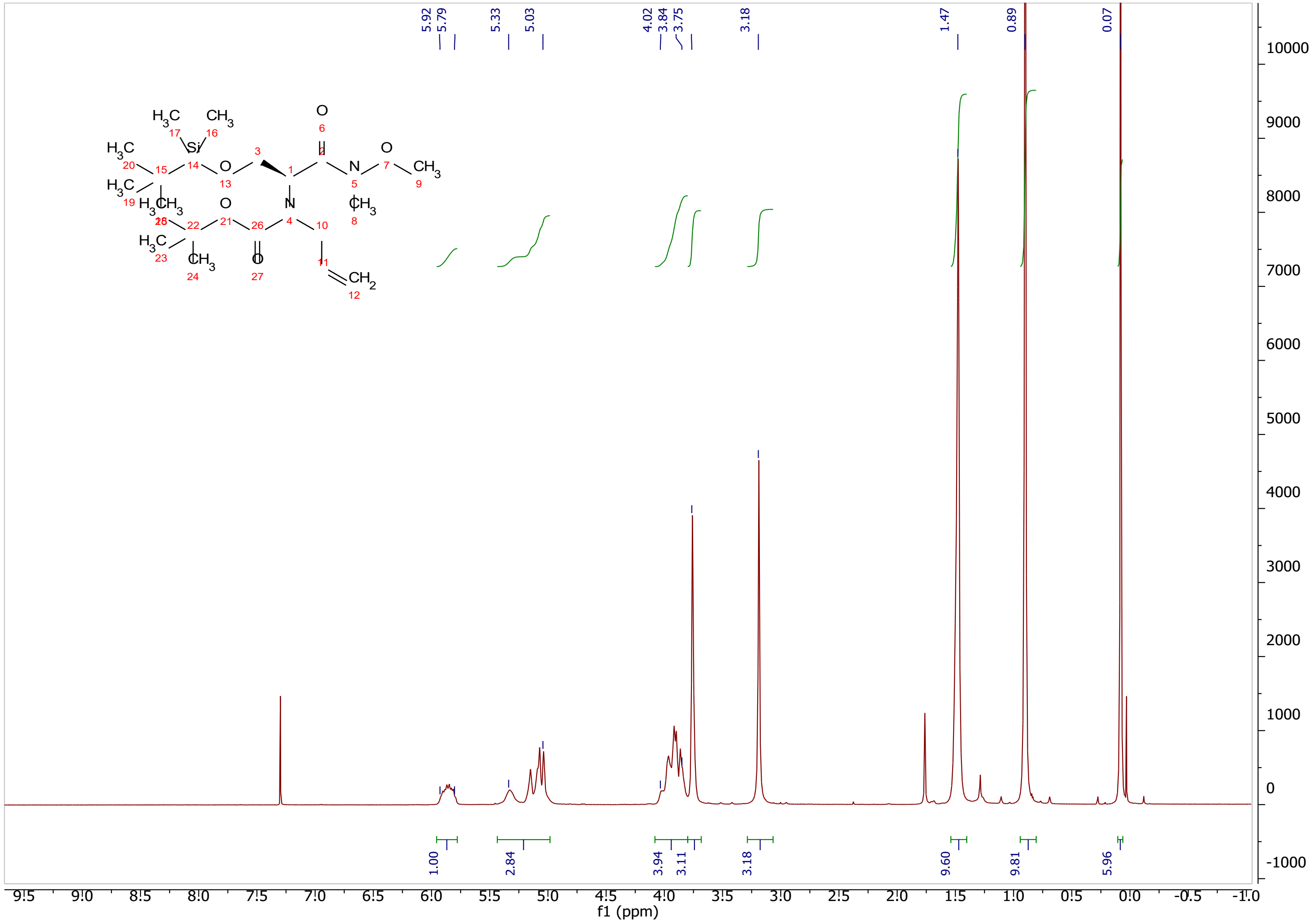

Figure S17: ^{13}C-NMR (75 MHz, $CDCl_3$) of ***tert*-butyl (*S*)-allyl(3,8,8,9,9-pentamethyl-4-oxo-2,7-dioxa-3-aza-8-siladecan-5-yl)carbamate**.

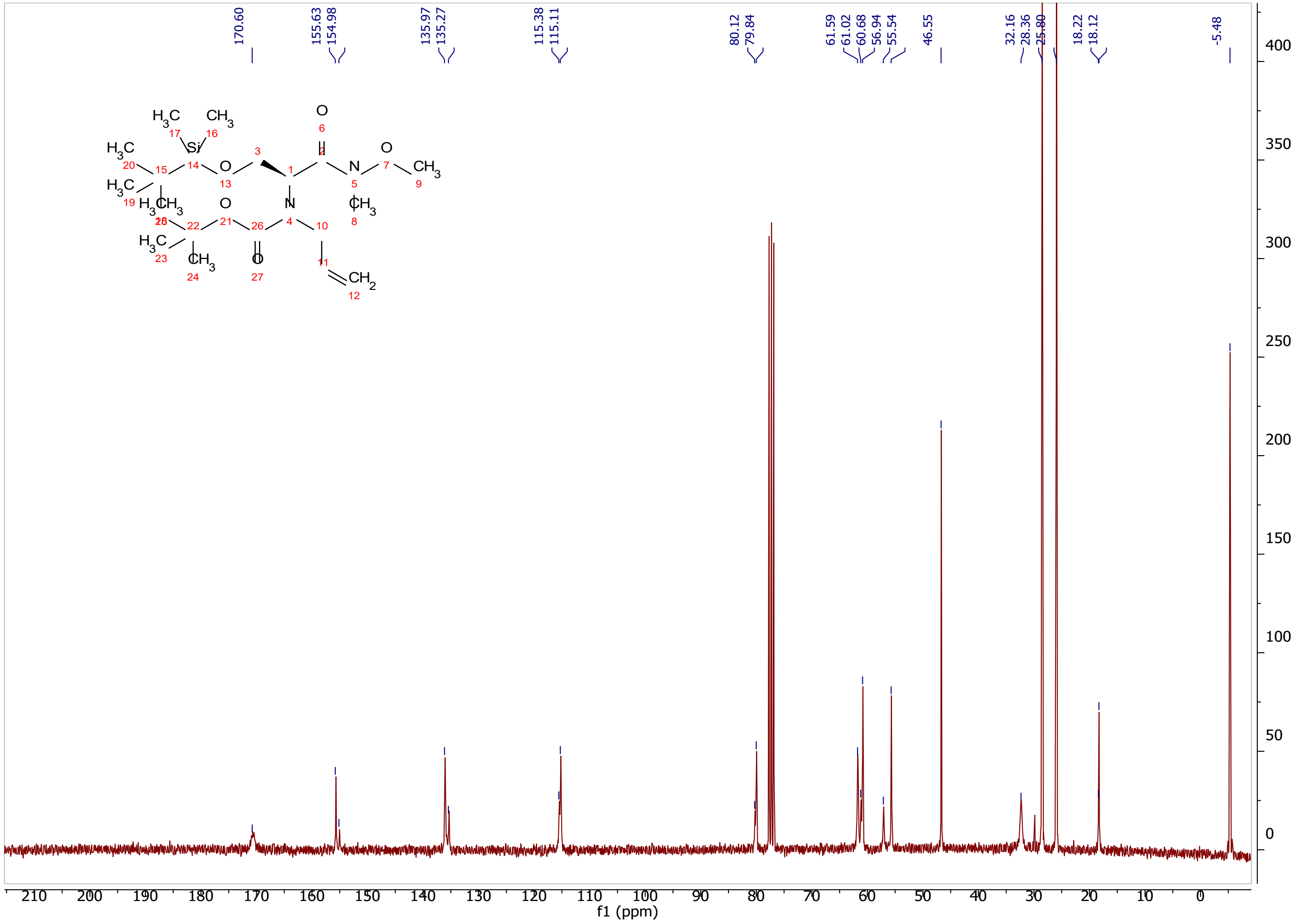

Figure S18: ^{1}H-NMR (400 MHz, $CDCl_3$) of **2b.**

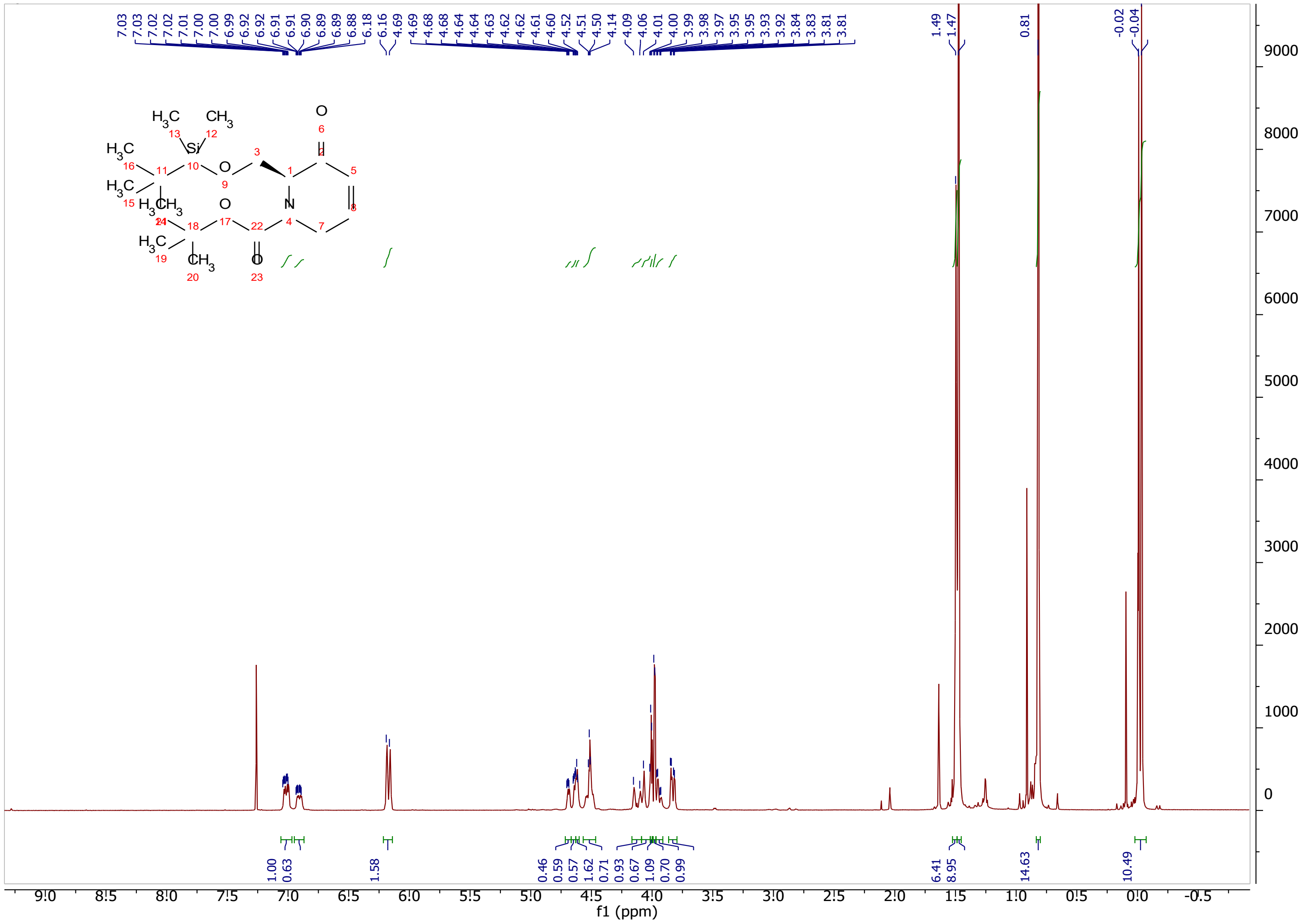

Figure S19: ^{13}C-NMR (100 MHz, $CDCl_3$) of **2b**.

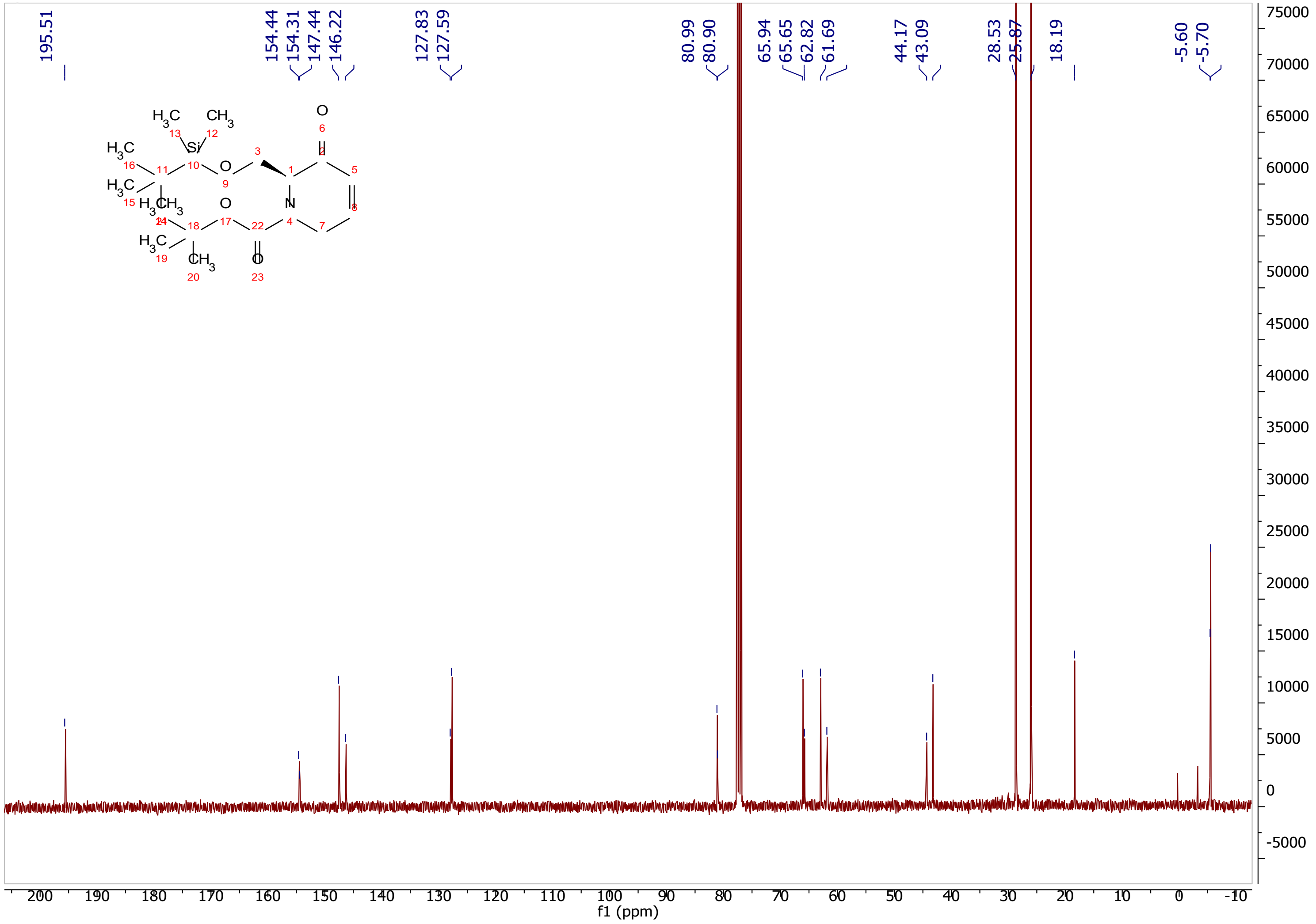

Figure S20: ^{1}H-NMR (400 MHz, $CDCl_3$) of **3b.**

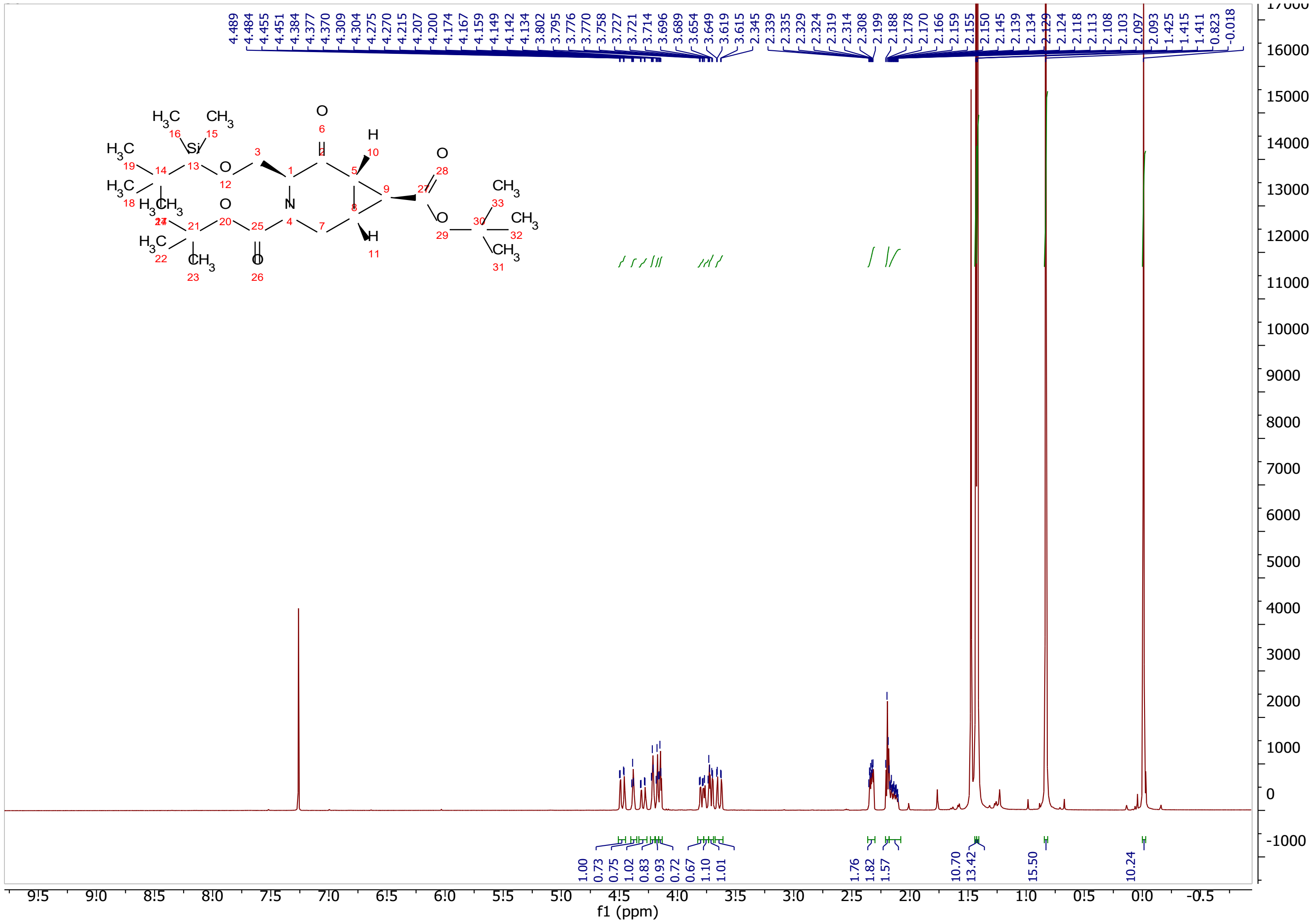

Figure S21: ^{13}C-NMR (100 MHz, $CDCl_3$) of **3b**.

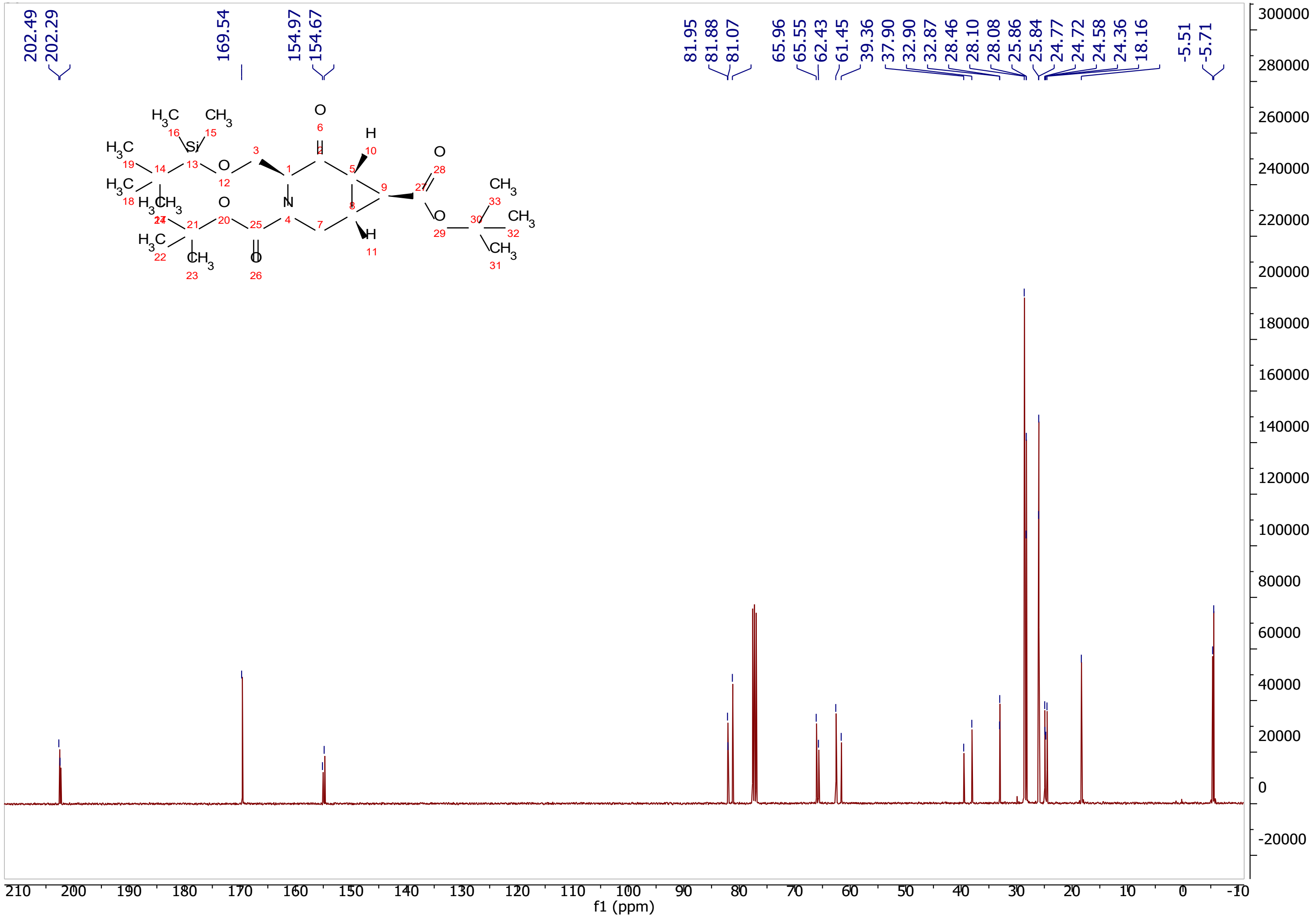

Figure S22: ^{1}H (400 MHz $CDCl_3$) bidimensional NOESY of **3b**.

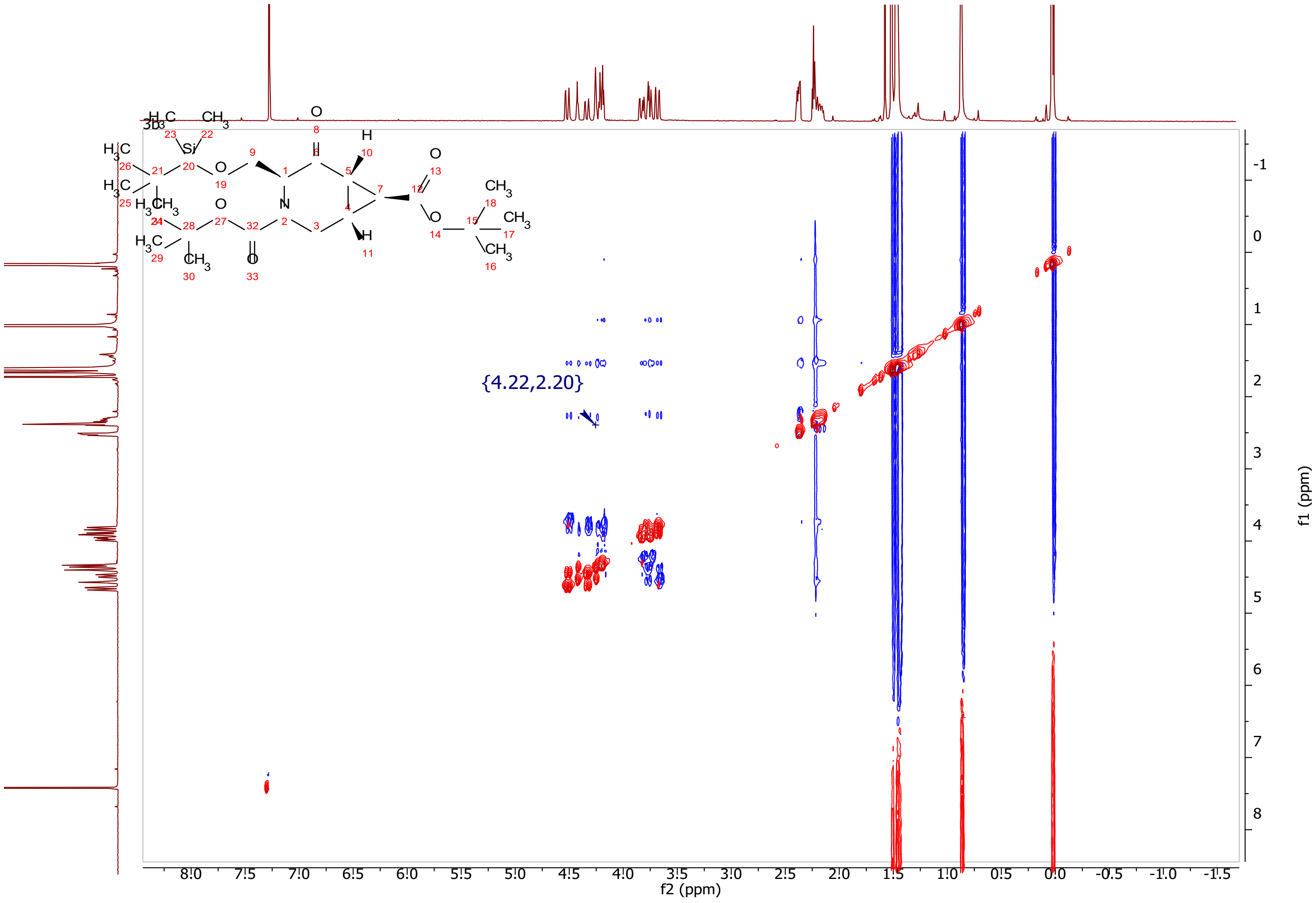

Figure S23: ^{1}H-NMR (400 MHz, $CDCl_3$) of **3b** in DMSO-d_6.

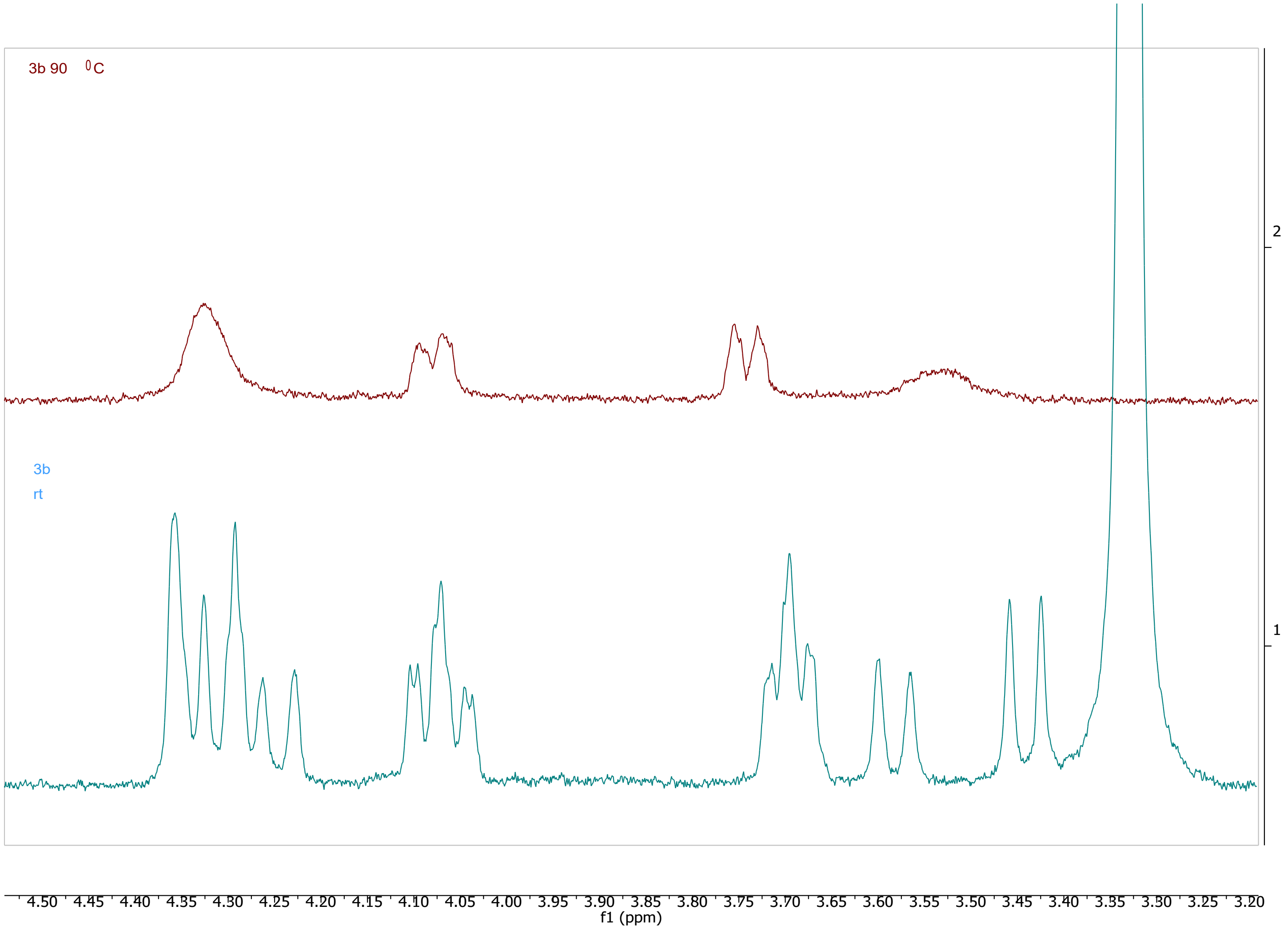

Figure S24: ^{1}H-NMR (400 MHz, $CDCl_3$) of **4b.**

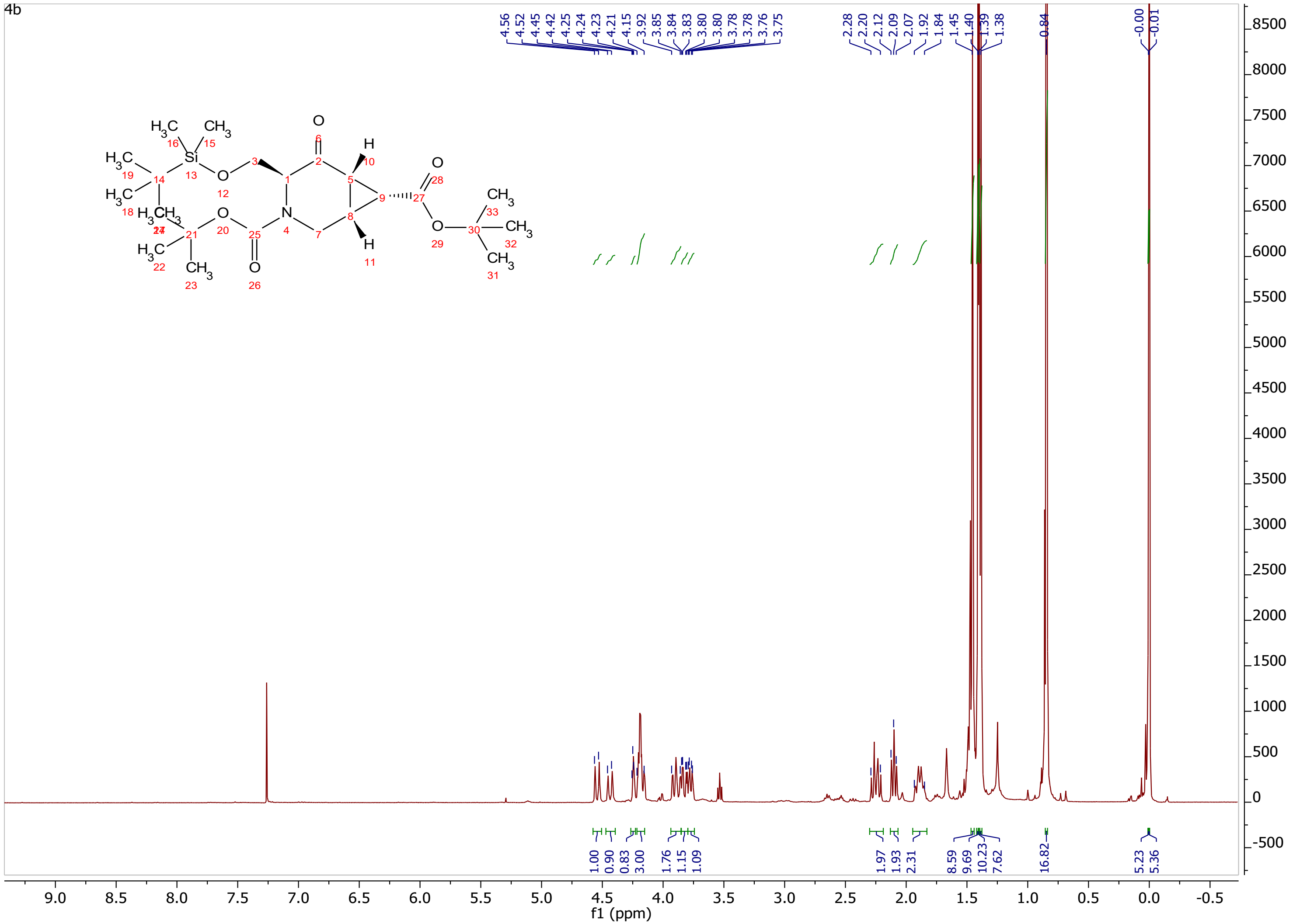

Figure S25: ^{13}C-NMR (100 MHz, $CDCl_3$) of **4b**.

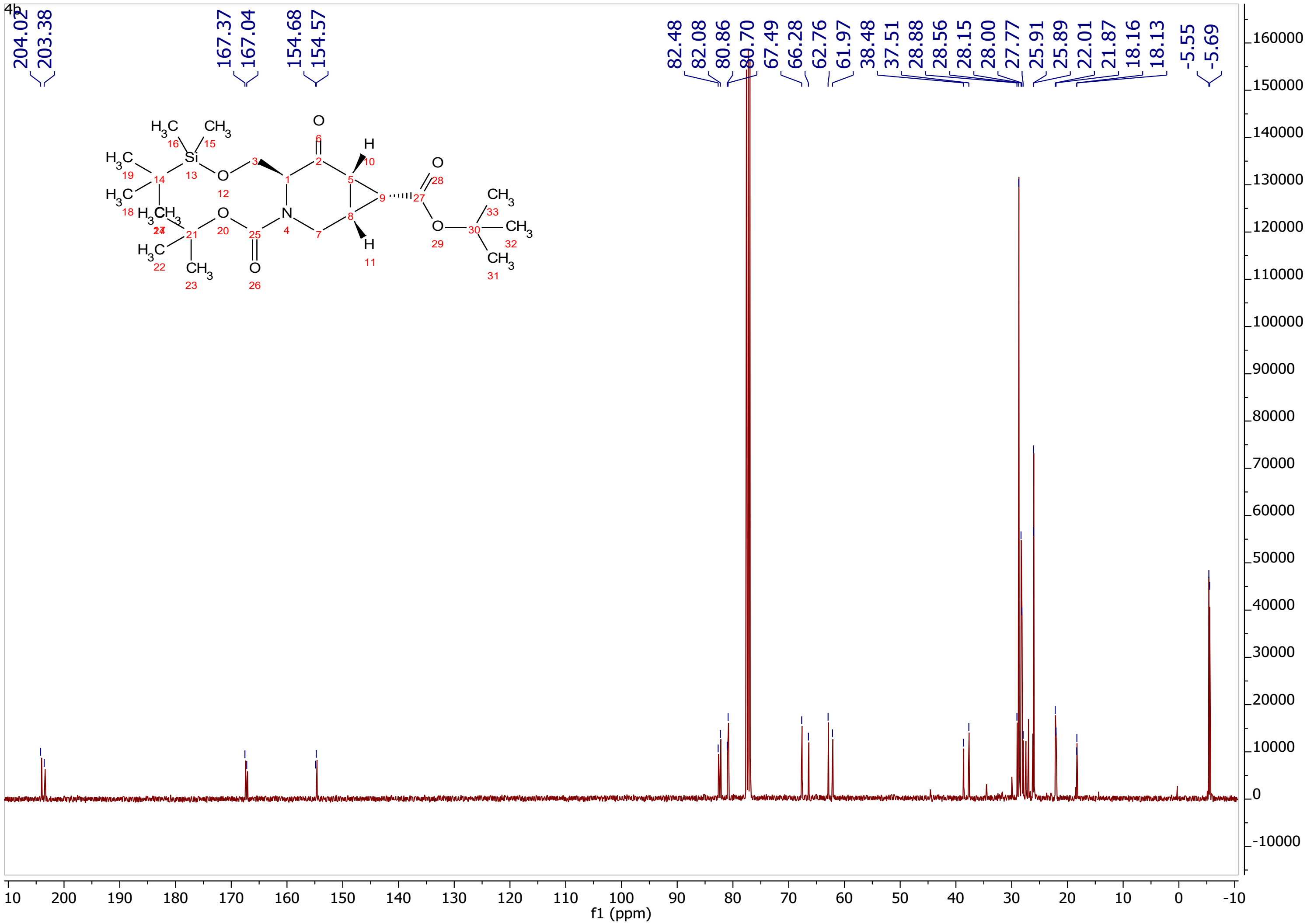

Figure S26: ^1H (400 MHz $CDCl_3$) bidimensional NOESY of **4b**.

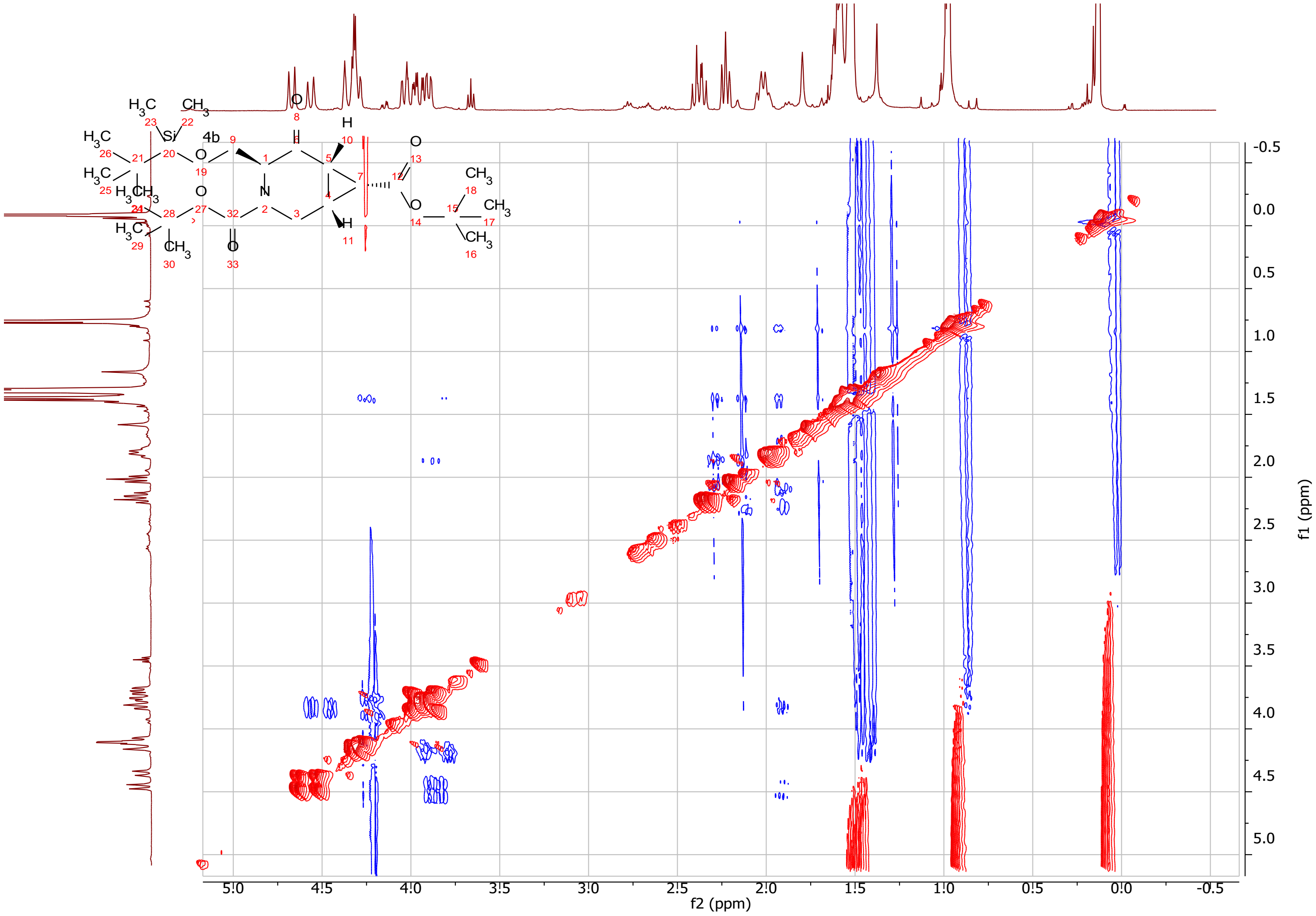

Figure S27: ^{1}H-NMR (400 MHz, $CDCl_3$) of **5a.**

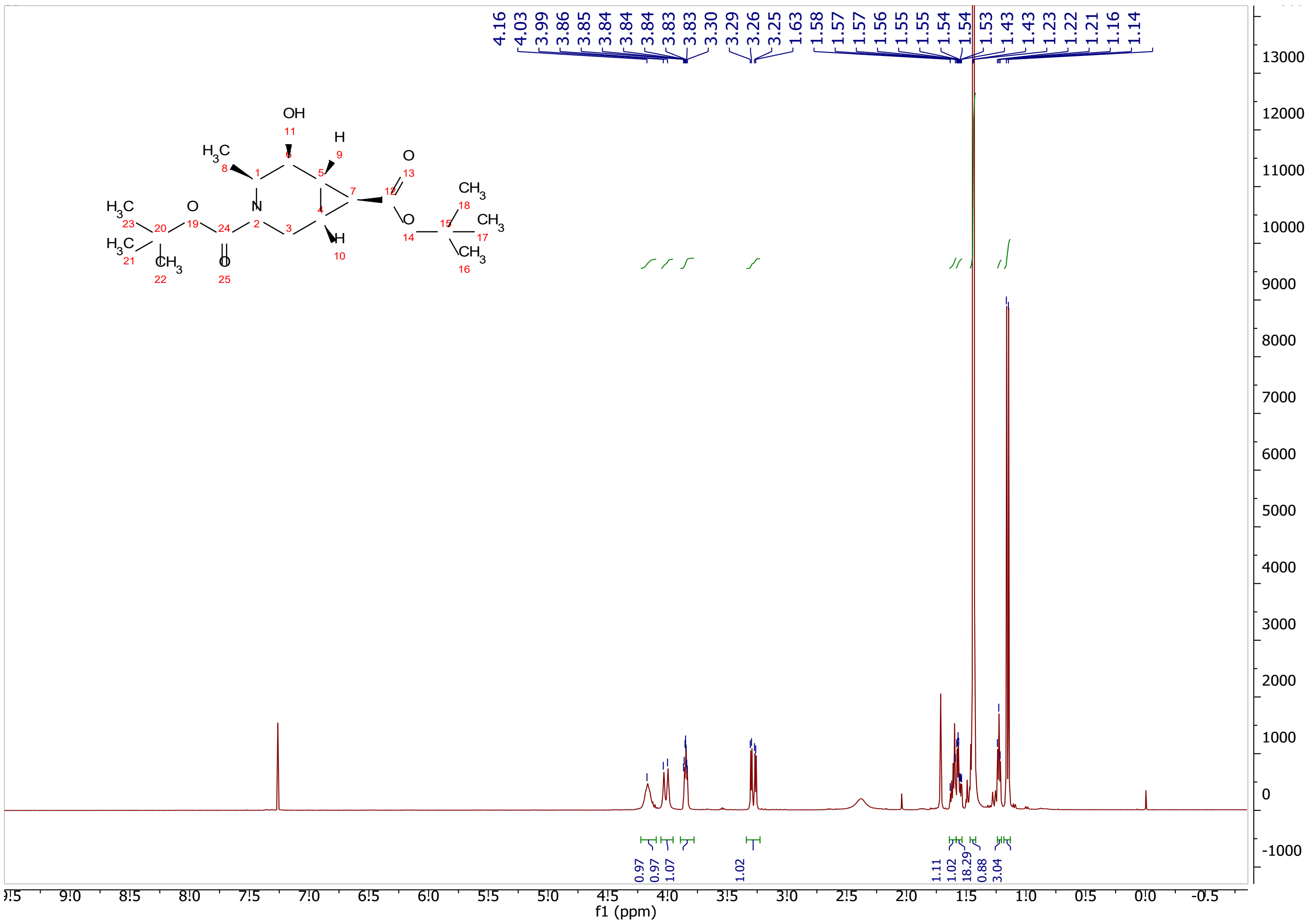

Figure S28: ^{13}C-NMR (100 MHz, $CDCl_3$) of **5a**.

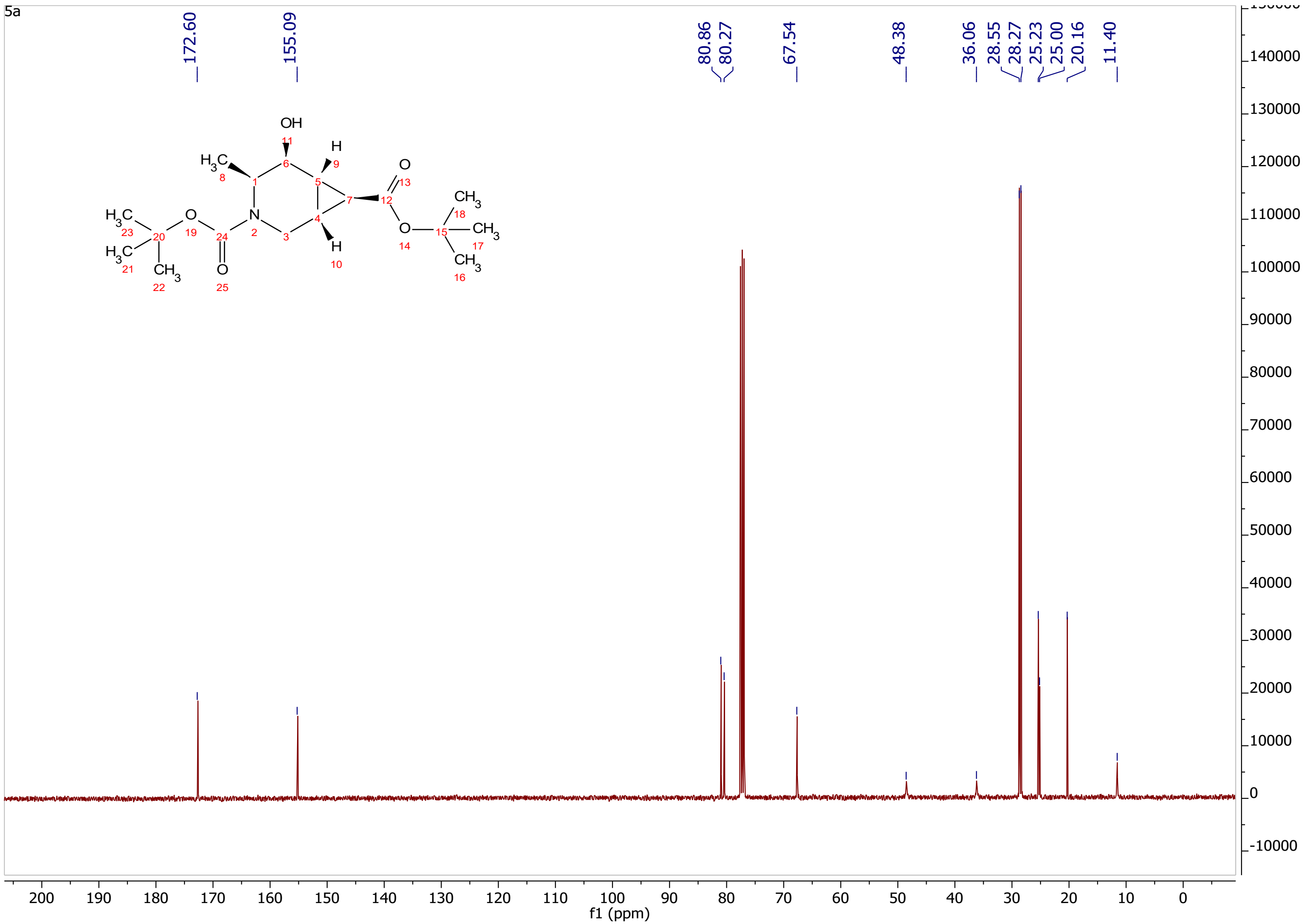

Figure S29: ¹H (400 MHz $CDCl_3$) bidimensional NOESY of **5a**.

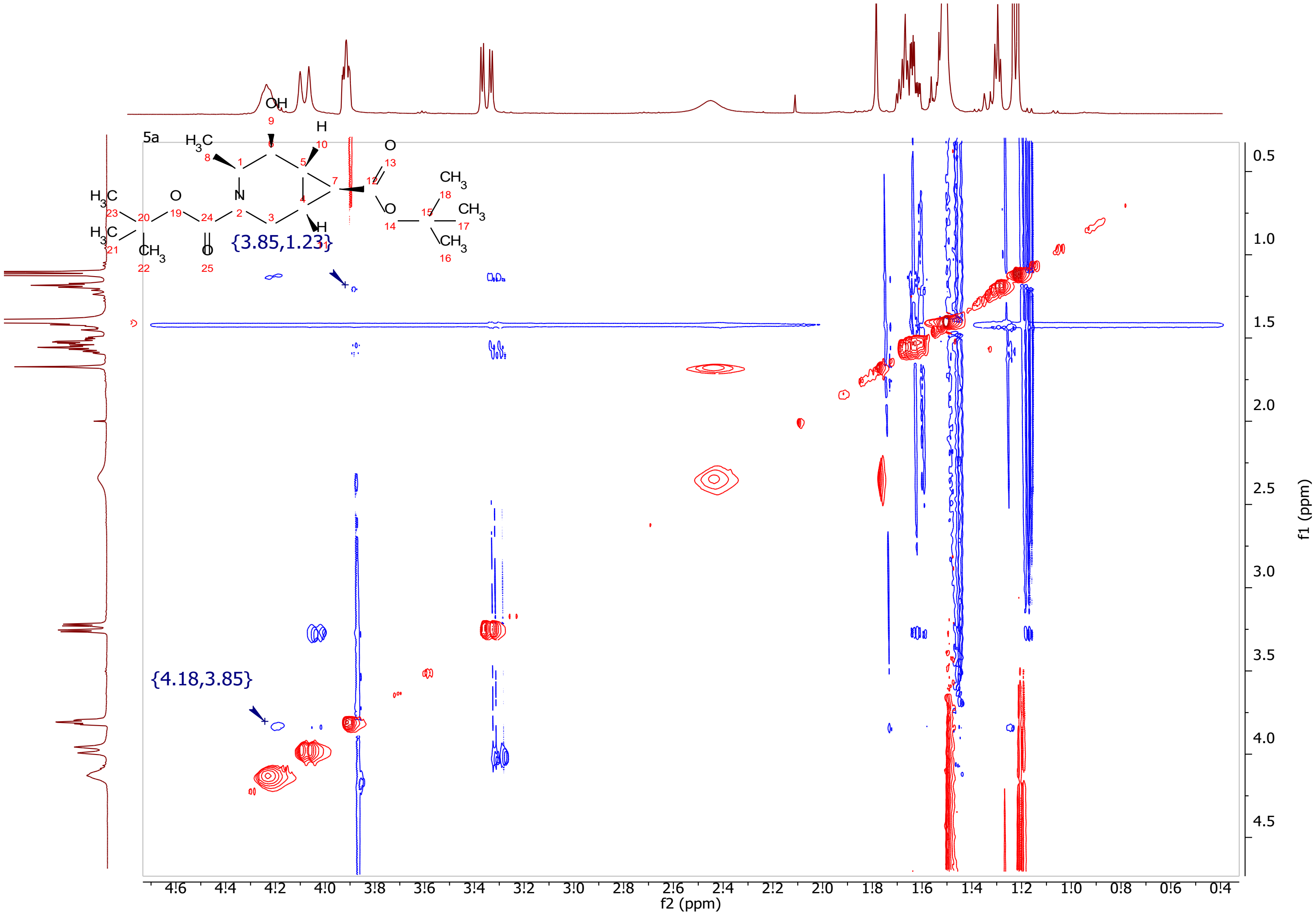

Figure S30: ^{1}H-NMR (400 MHz, $CDCl_3$) of **5b.**

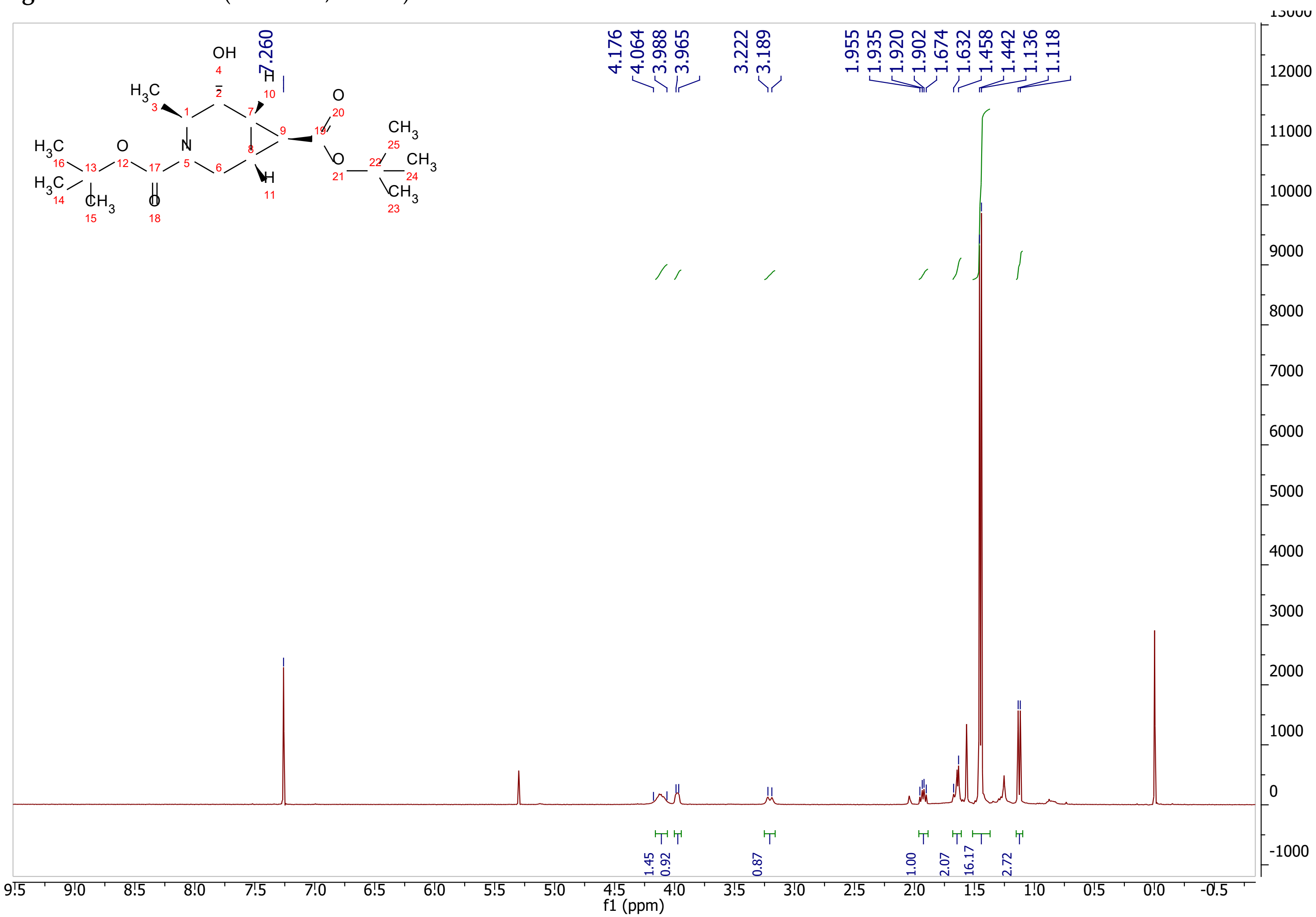

Figure S31: ^{13}C-NMR (100 MHz, $CDCl_3$) of **5b**.

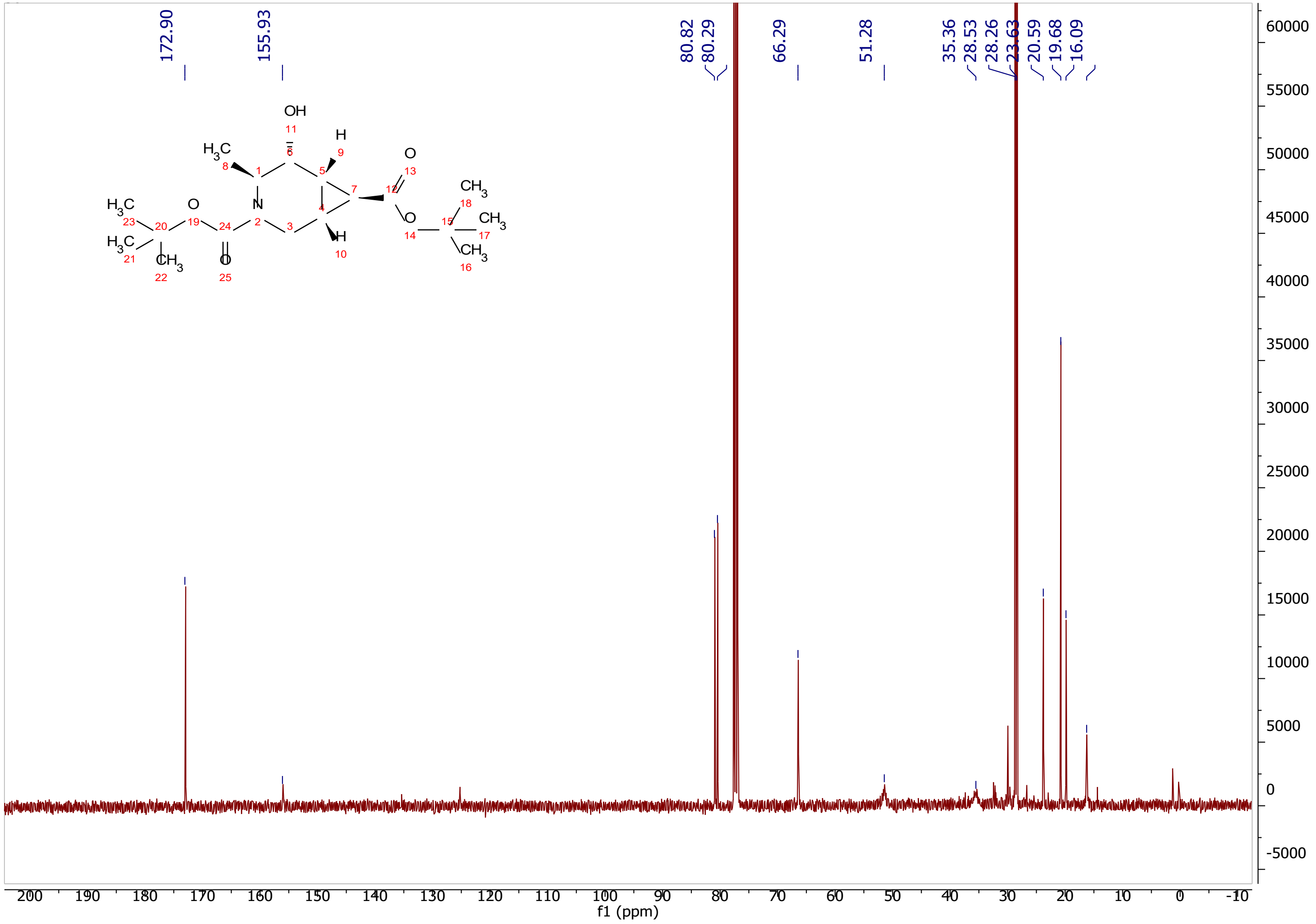

Figure S32: ^{1}H (400 MHz $CDCl_3$) bidimensional NOESY of **5b**.

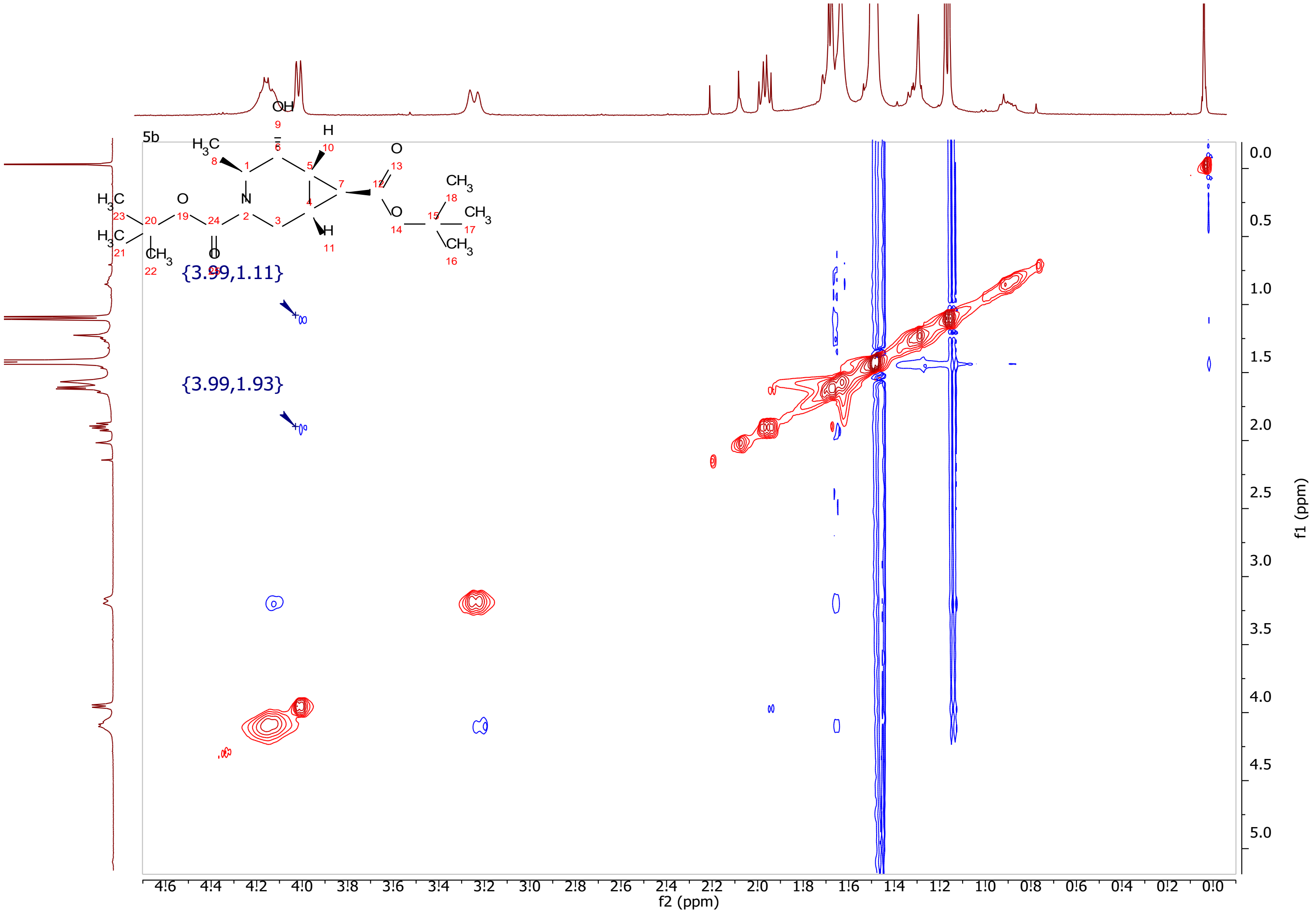

Figure S33: ^{1}H-NMR (400 MHz, $CDCl_3$) of ***tert*-butyl (1*S*,4*S*,6*S*,7*S*)-5-hydroxy-7-(hydroxymethyl)-4-methyl-3-azabicyclo[4.1.0]heptane-3- carboxylate**.

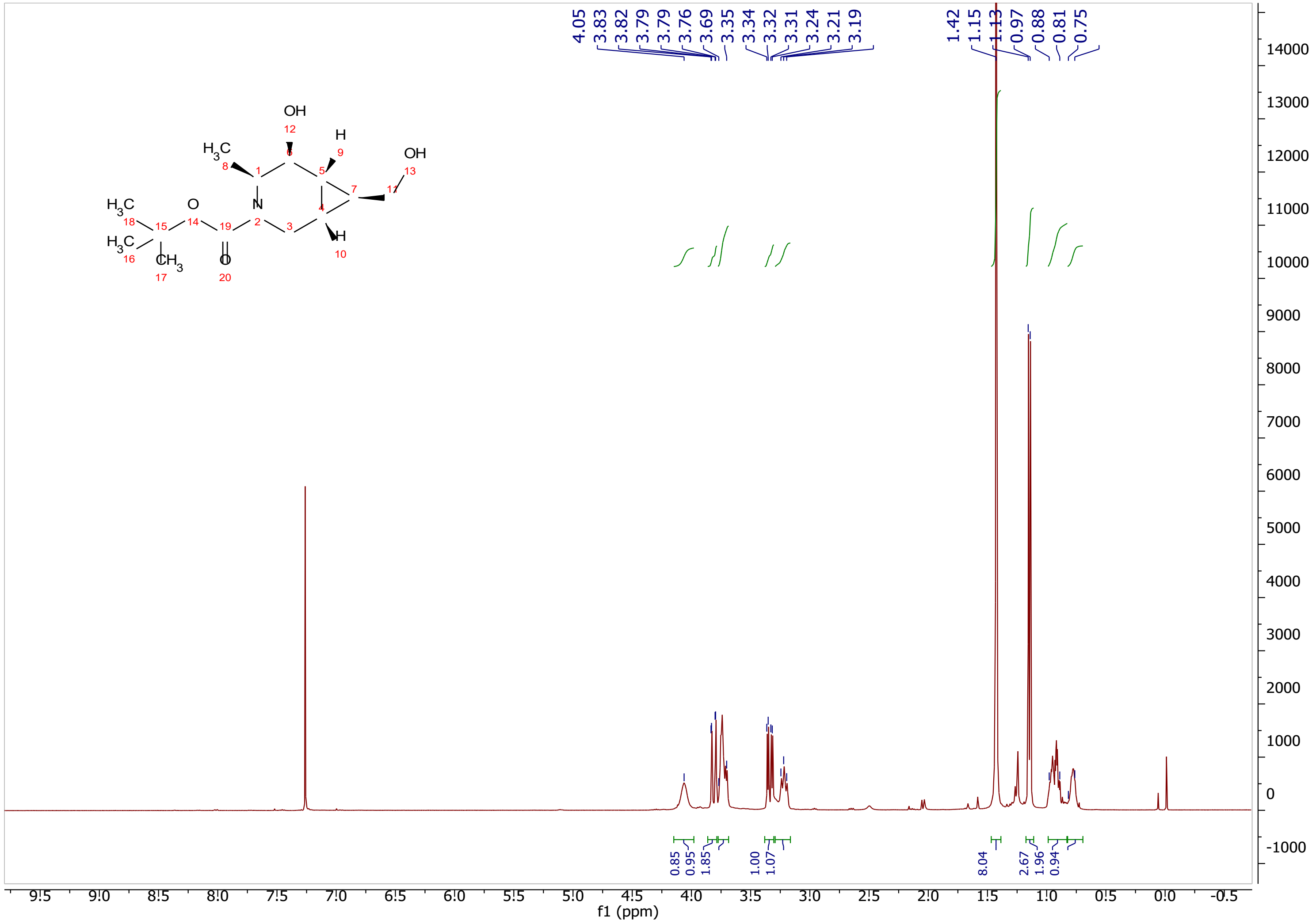

Figure S34: ^{13}C-NMR (100 MHz, $CDCl_3$) of ***tert*-butyl (1*S*,4*S*,6*S*,7*S*)-5-hydroxy-7-(hydroxymethyl)-4-methyl-3-azabicyclo[4.1.0]heptane-3- carboxylate**.

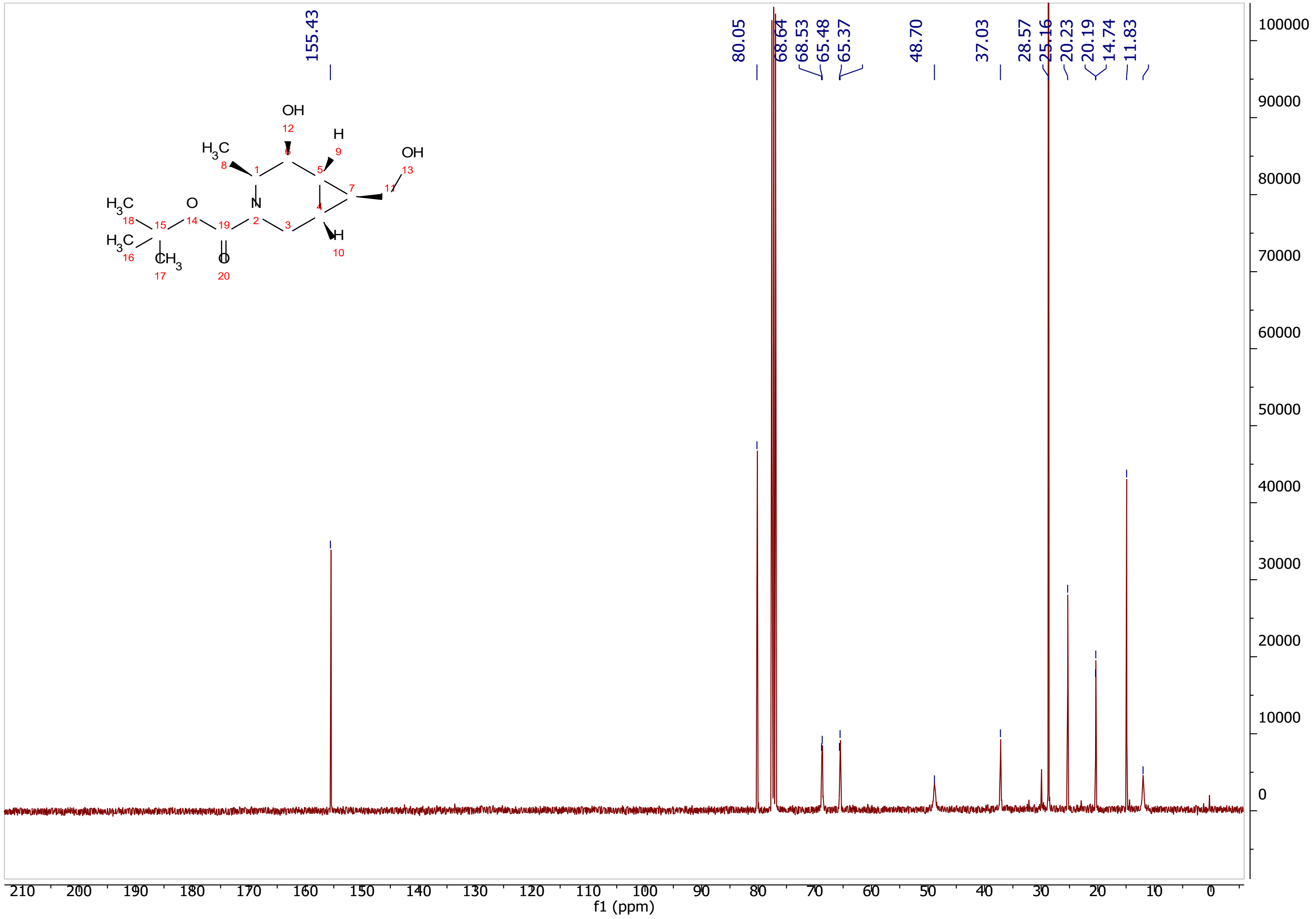

Figure S35: ^{1}H-NMR (300 MHz, MeOD) of **6a**.

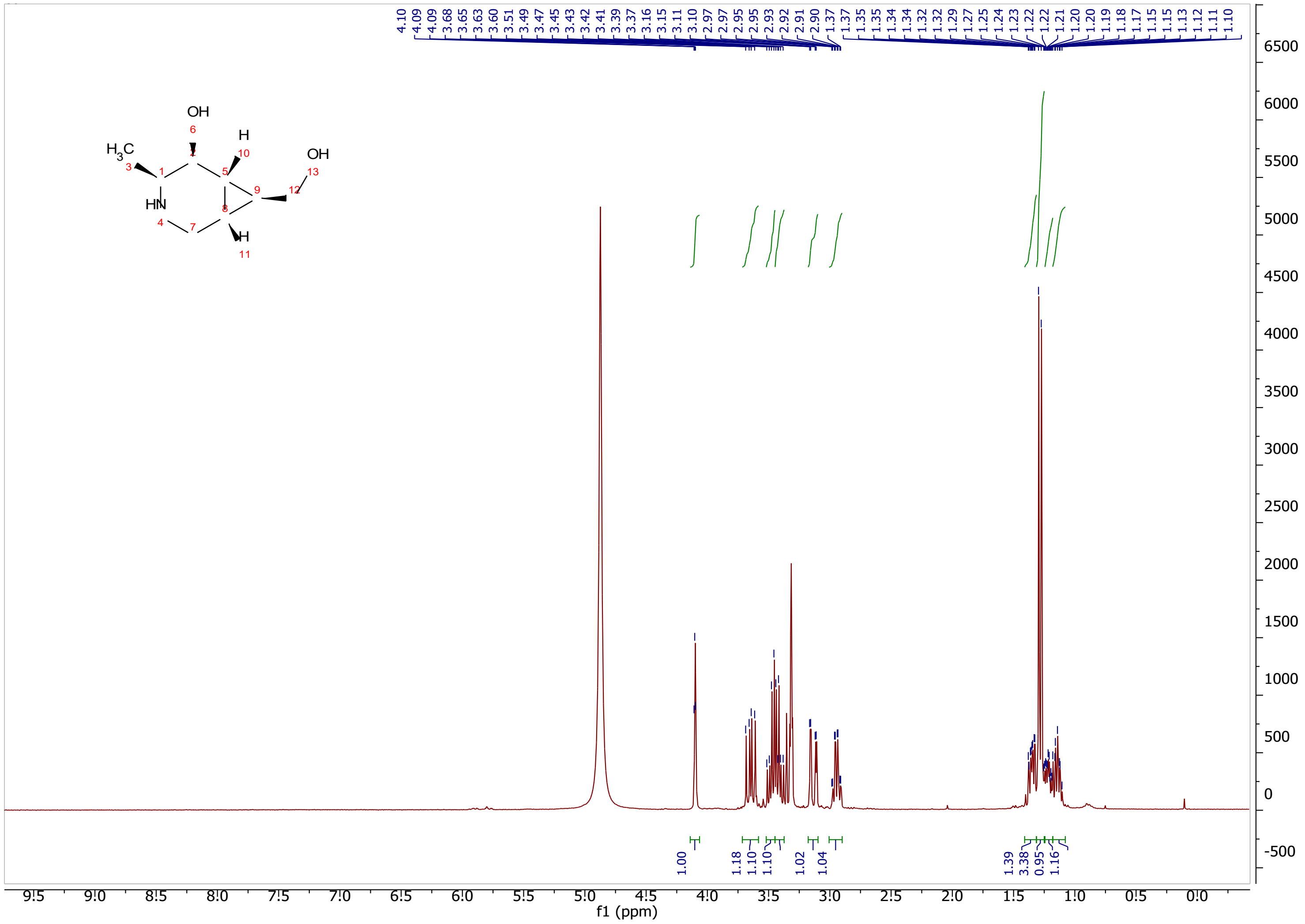

Figure S36: ^{13}C-NMR (75 MHz, MeOD) of **6a**.

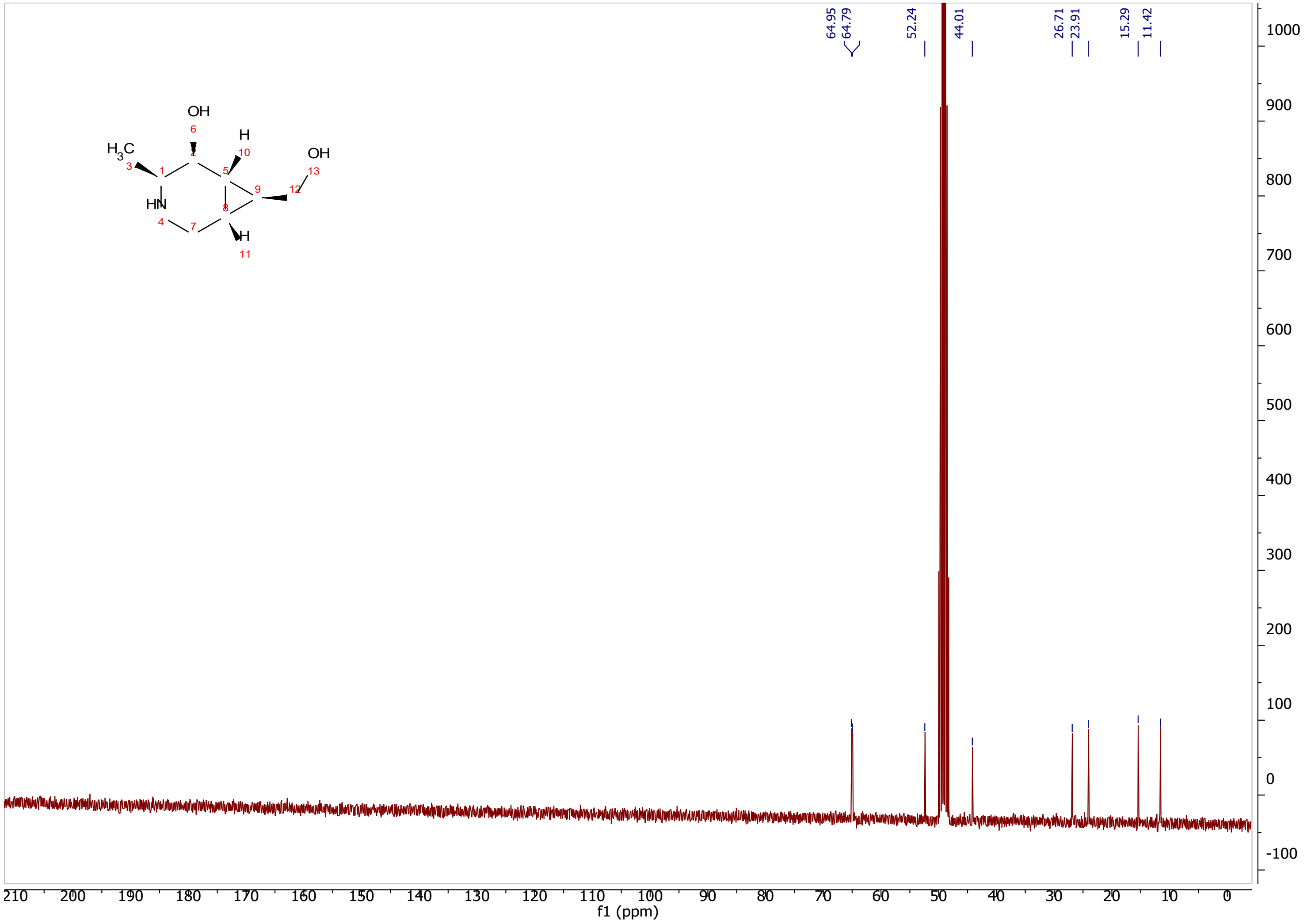

Figure S37: ^{1}H-NMR (400 MHz, $CDCl_3$) of ***tert*-butyl (1*S*,4*S*,5*R*,6*S*,7*S*)-5-hydroxy-7-(hydroxymethyl)-4-methyl-3-azabicyclo[4.1.0] heptane-3-carboxylate**.

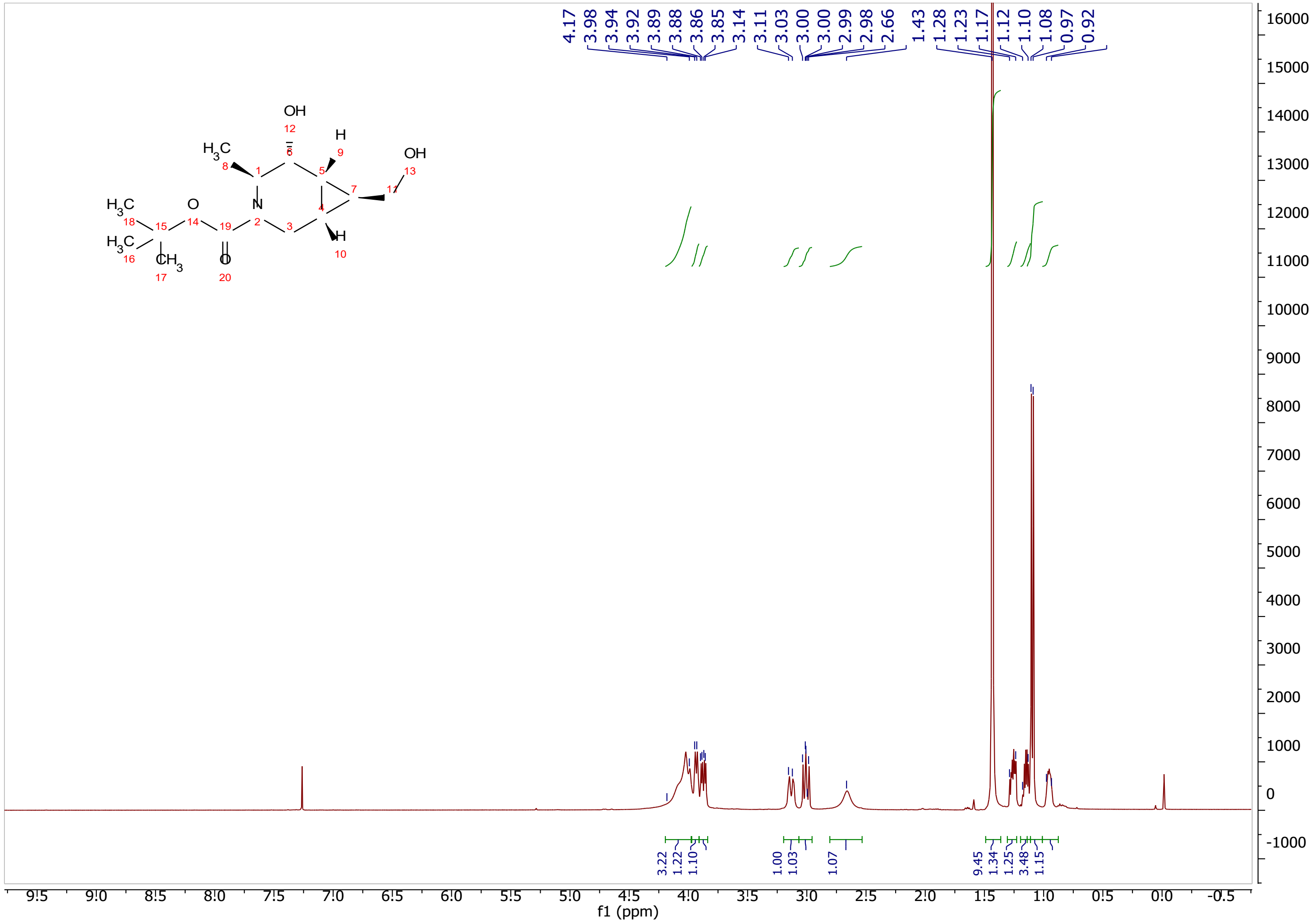

Figure S38: ^{13}C-NMR (100 MHz, $CDCl_3$) of ***tert*-butyl (1*S*,4*S*,5*R*,6*S*,7*S*)-5-hydroxy-7-(hydroxymethyl)-4-methyl-3-azabicyclo[4.1.0] heptane-3-carboxylate**.

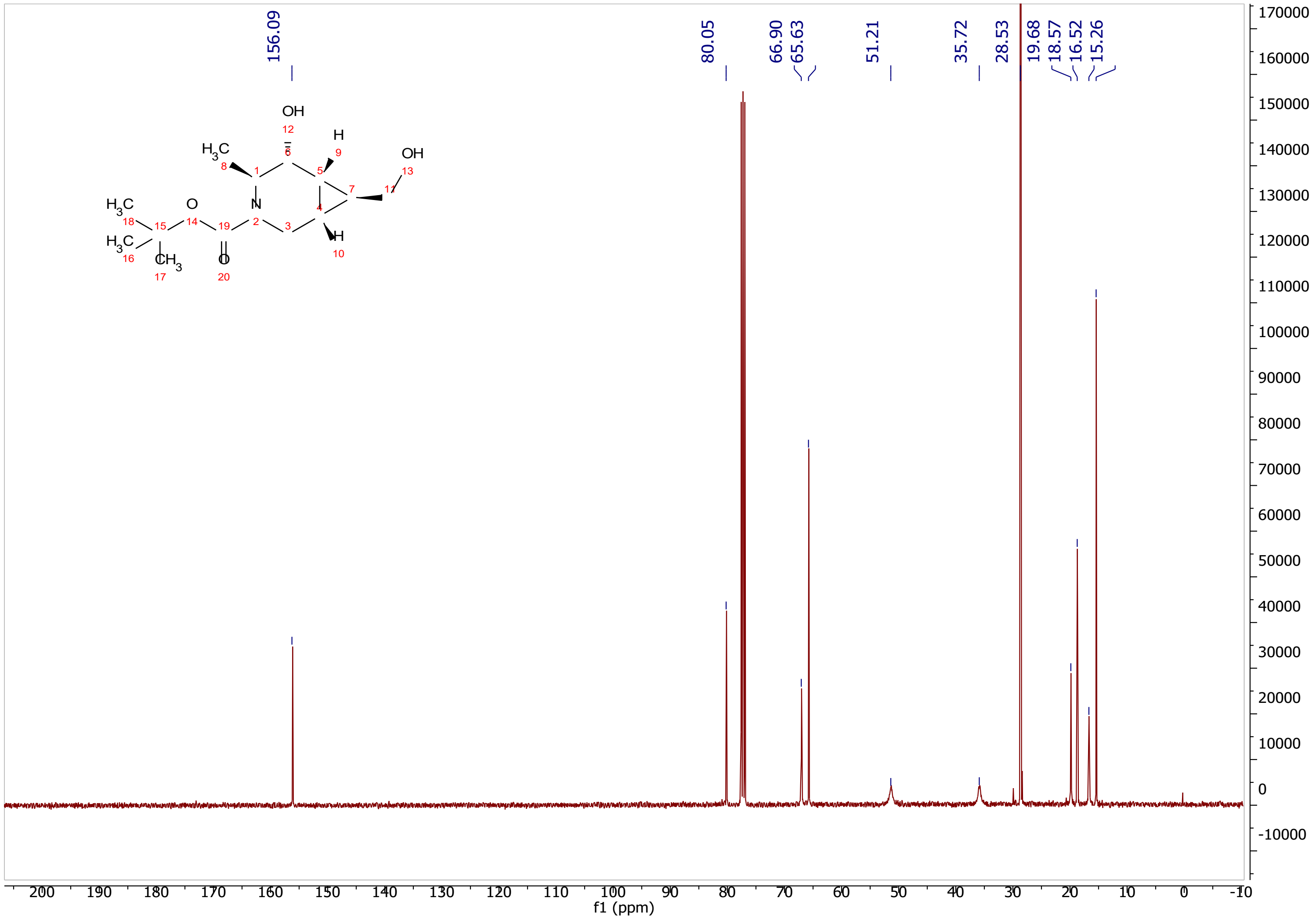

Figure S39: ^{1}H-NMR (400 MHz, MeOD) of **6b**.

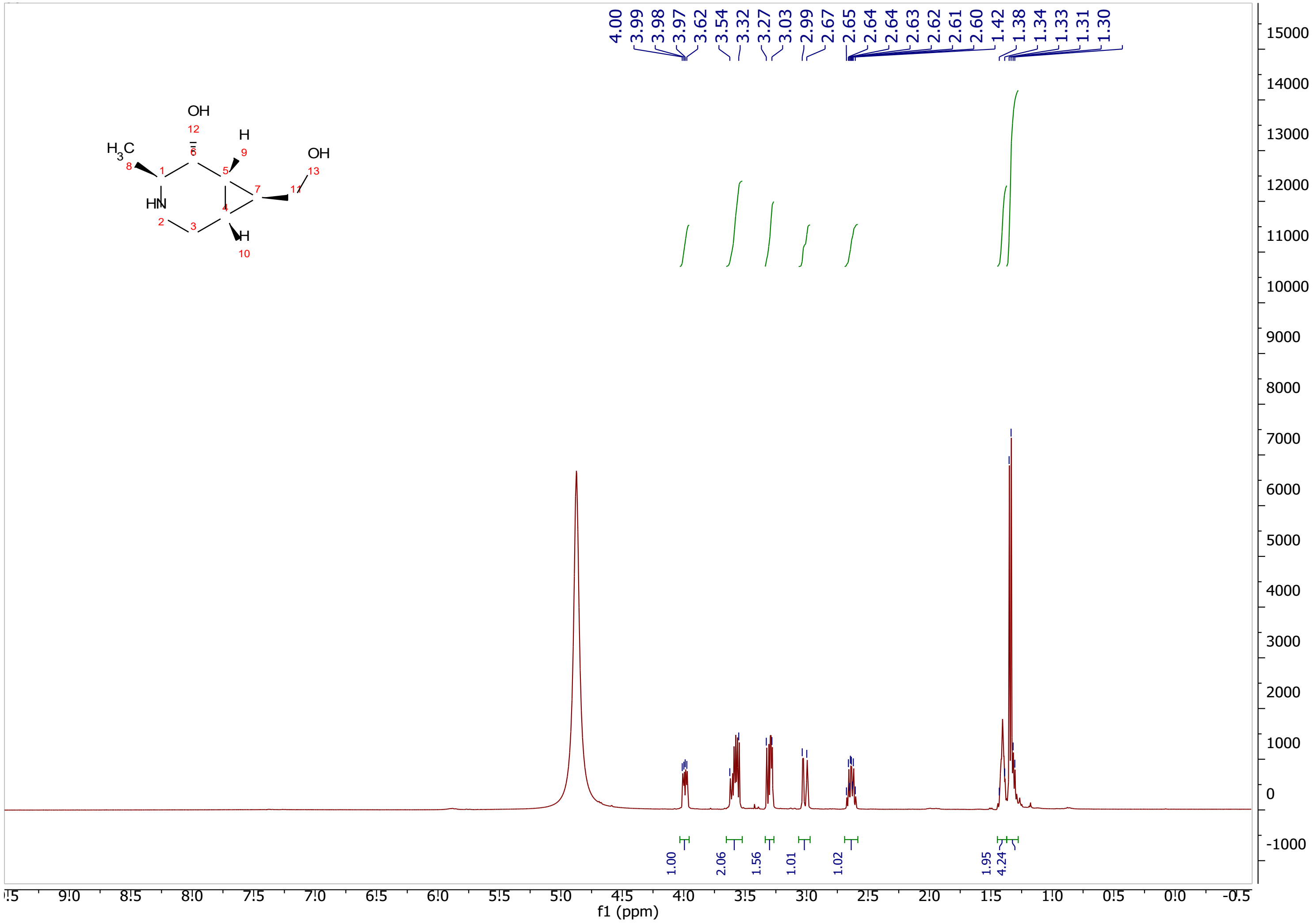

Figure S40: ^{13}C-NMR (100 MHz, MeOD) of **6b**.

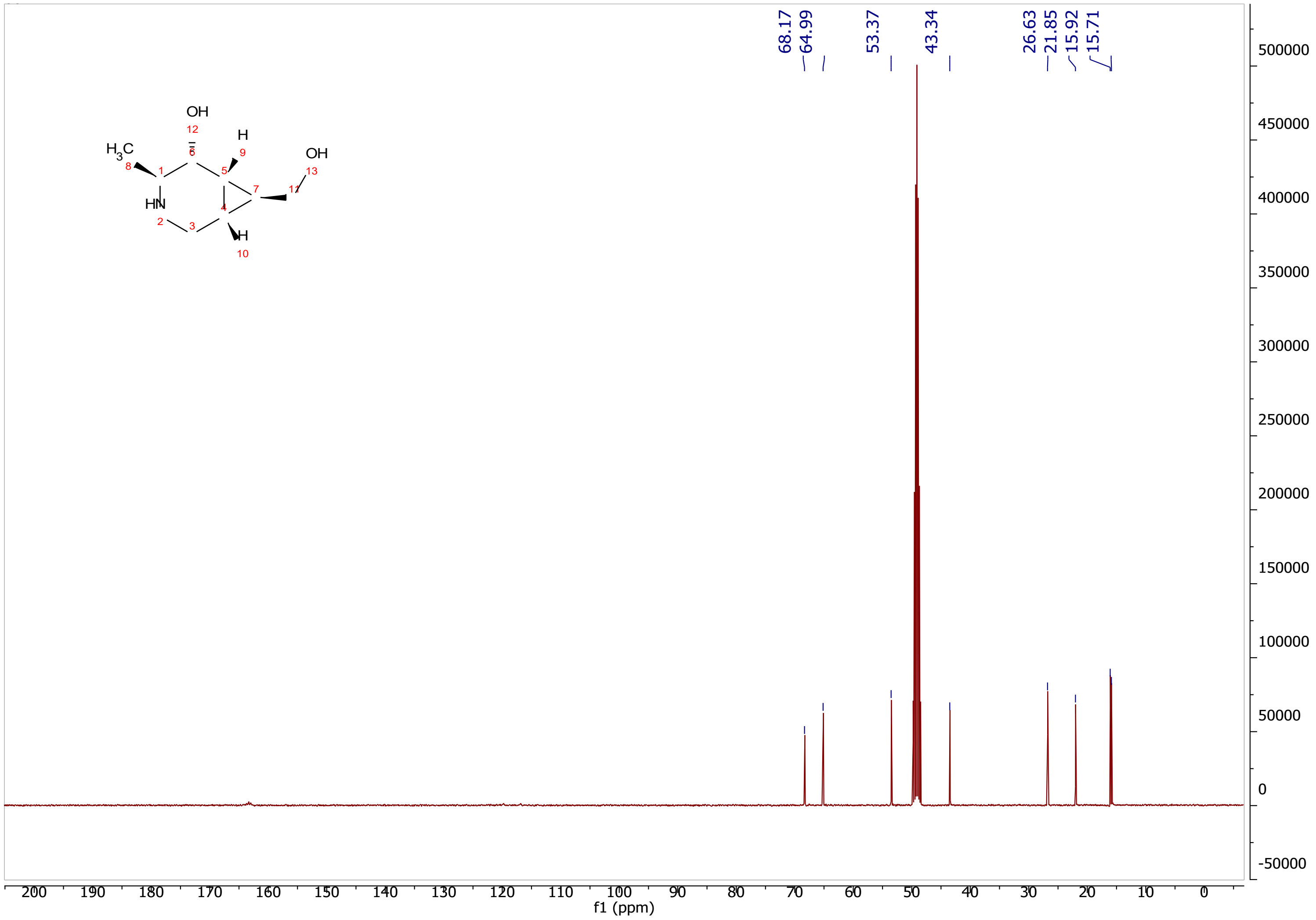

Figure S41: ^{1}H-NMR with water suppression (500 MHz, D_2O) of PNP-galactose, mixture of PNP-gal with galactosidase after 1 hour and mixture of PNP-gal, galactosidase and **6b** after 12 hours of enzymatic reaction respectively.

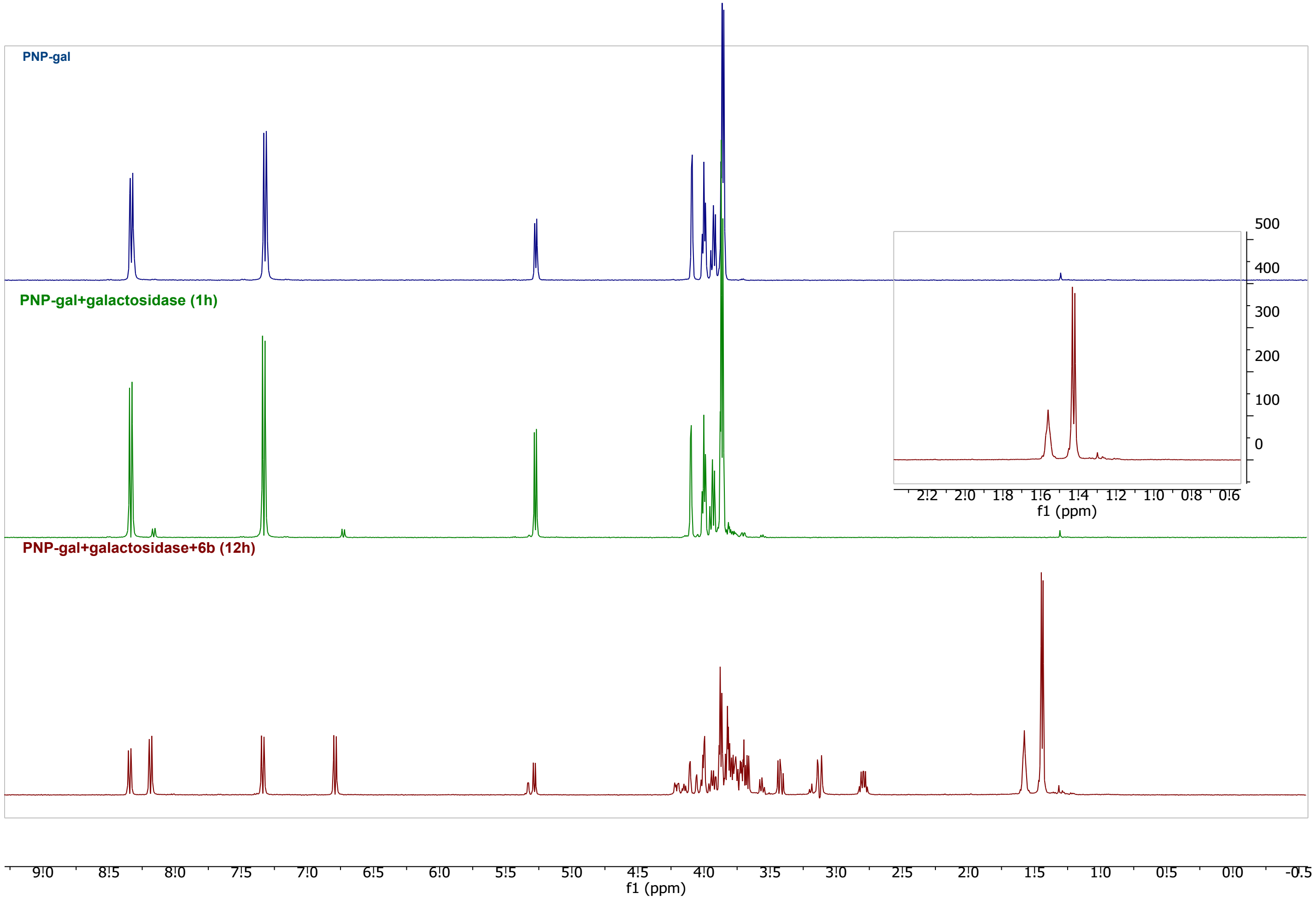

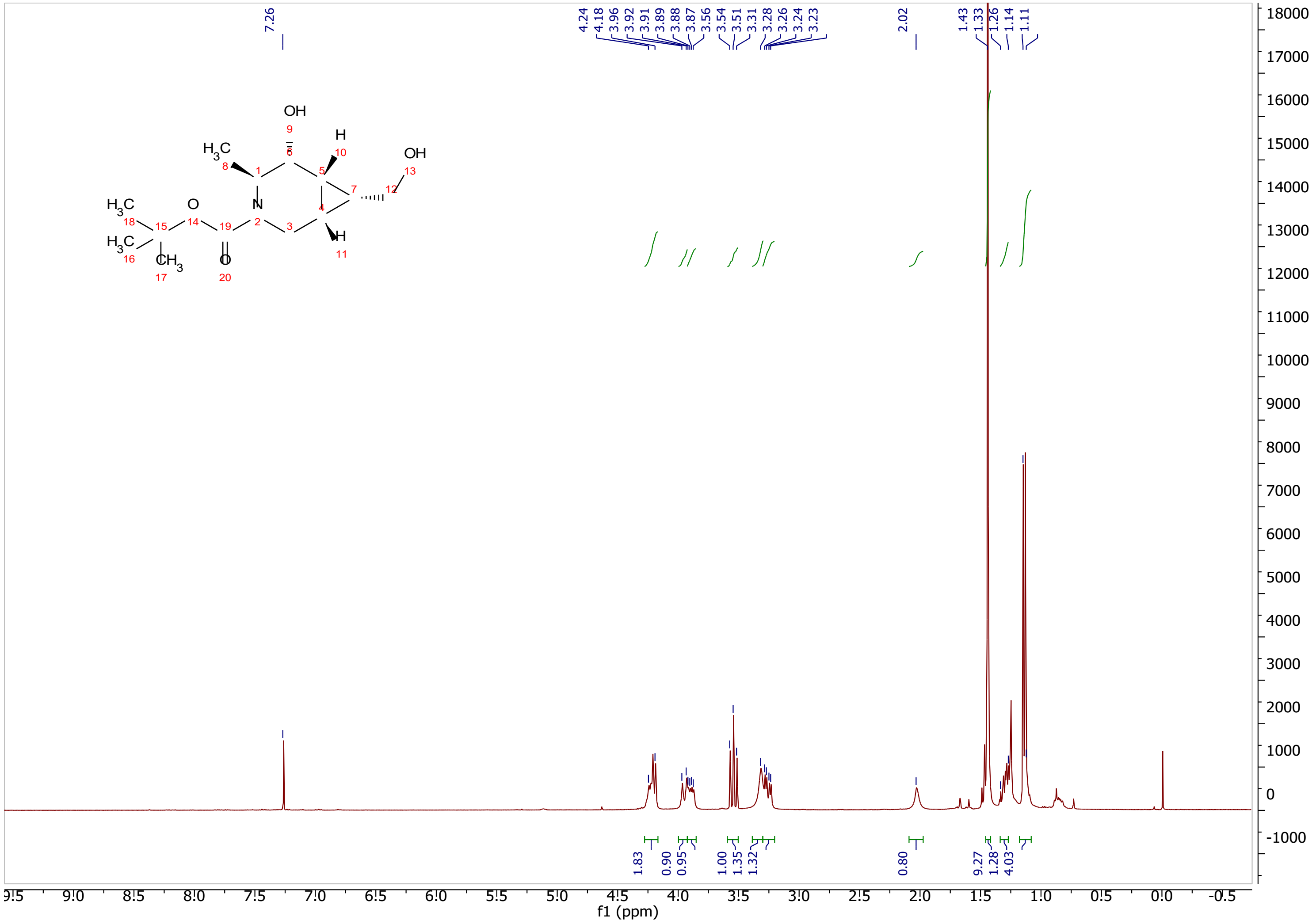

Figure S42: ^{1}H-NMR (400 MHz, $CDCl_3$) of ***tert*-butyl (1*S*,4*S*,5*R*,6*S*,7*R*)-5-hydroxy-7-(hydroxymethyl)-4-methyl-3-azabicyclo[4.1.0]heptane- 3-carboxylate**.

Figure S43: ^{13}C-NMR (100 MHz, $CDCl_3$) of ***tert*-butyl (1*S*,4*S*,5*R*,6*S*,7*R*)-5-hydroxy-7-(hydroxymethyl)-4-methyl-3-azabicyclo[4.1.0]heptane- 3-carboxylate**.

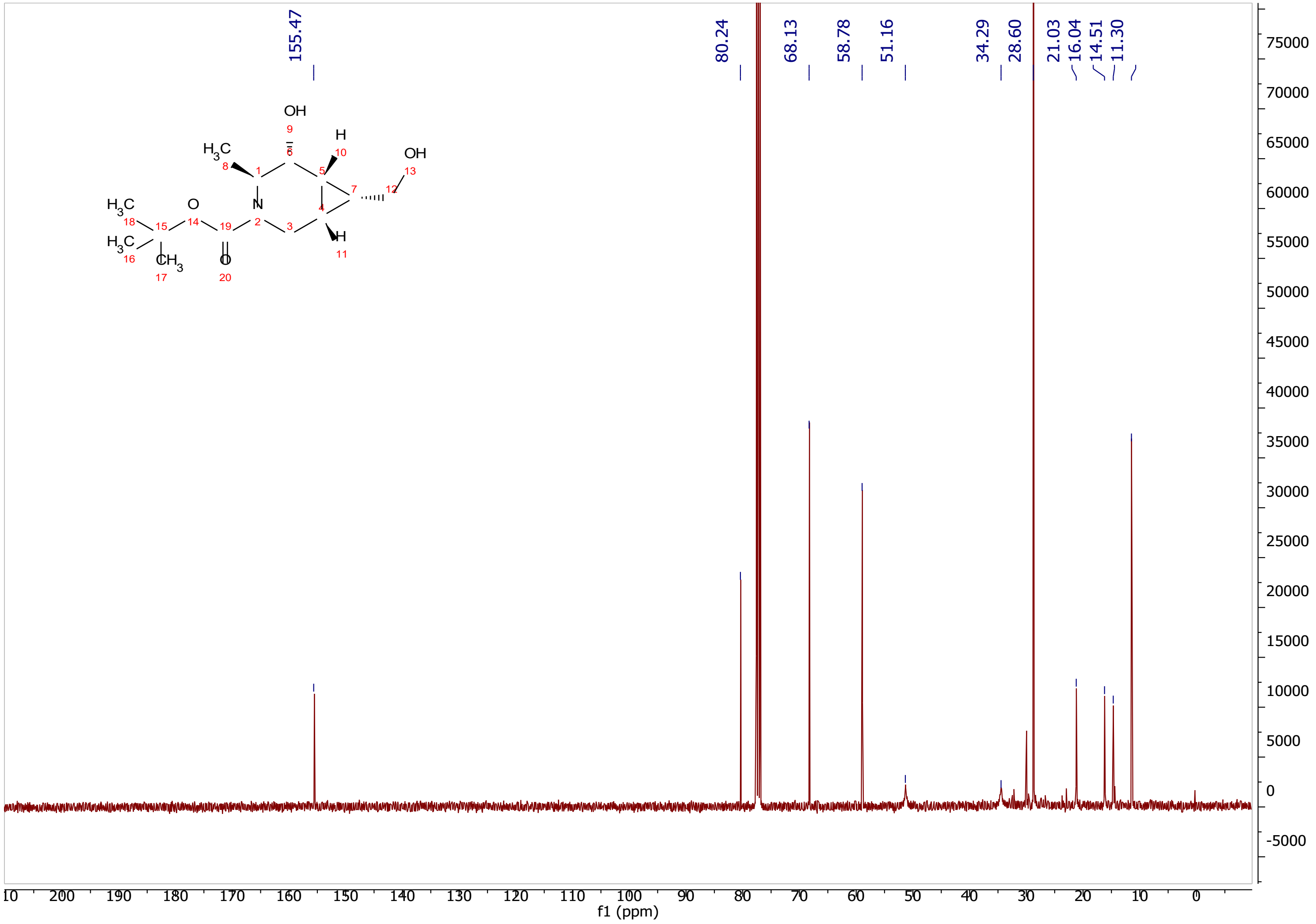

Figure S44: ^{1}H-NMR (400 MHz, MeOD) of **6c**.

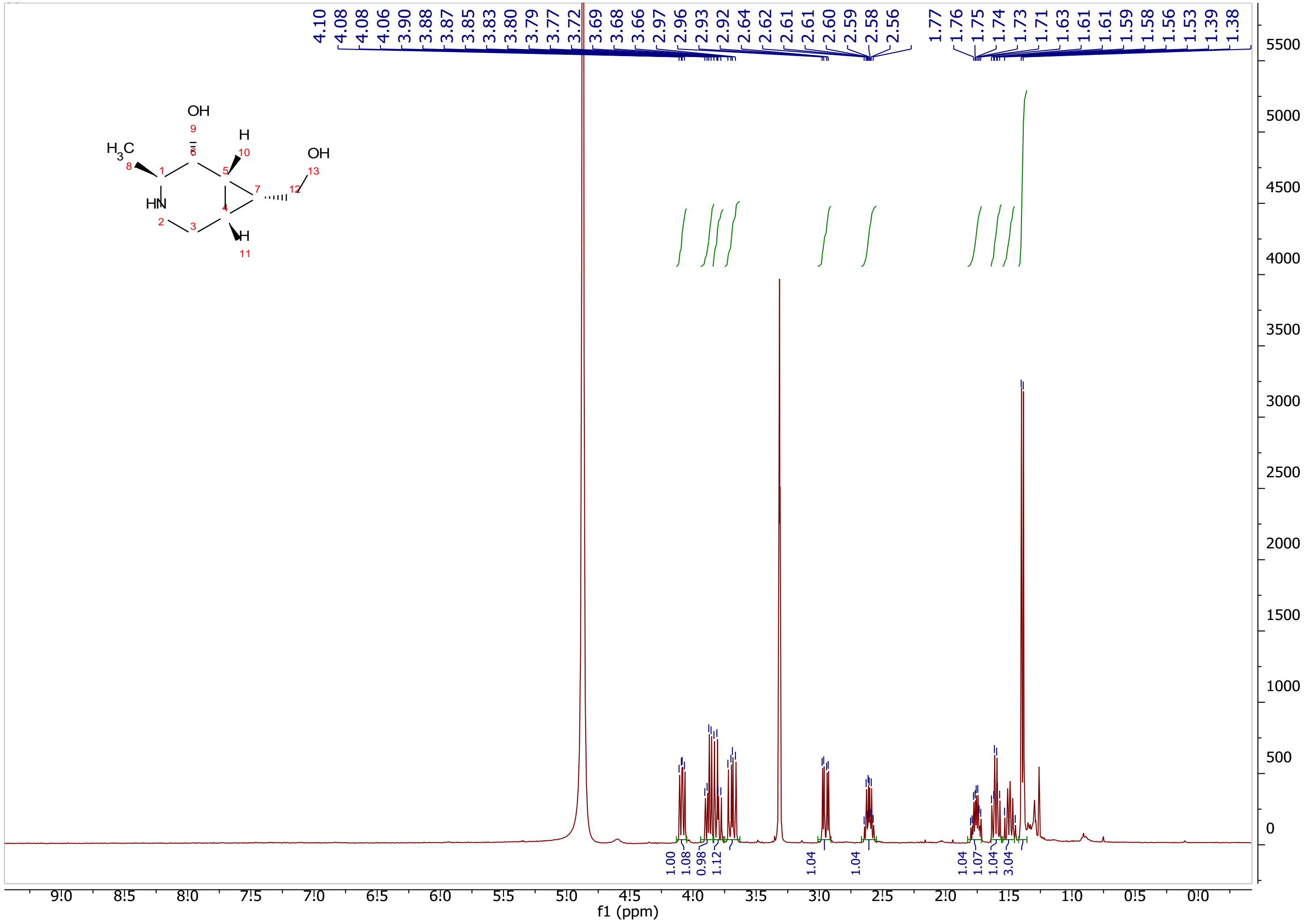

Figure S45: ^{13}C-NMR (100 MHz, MeOD) of **6c**.

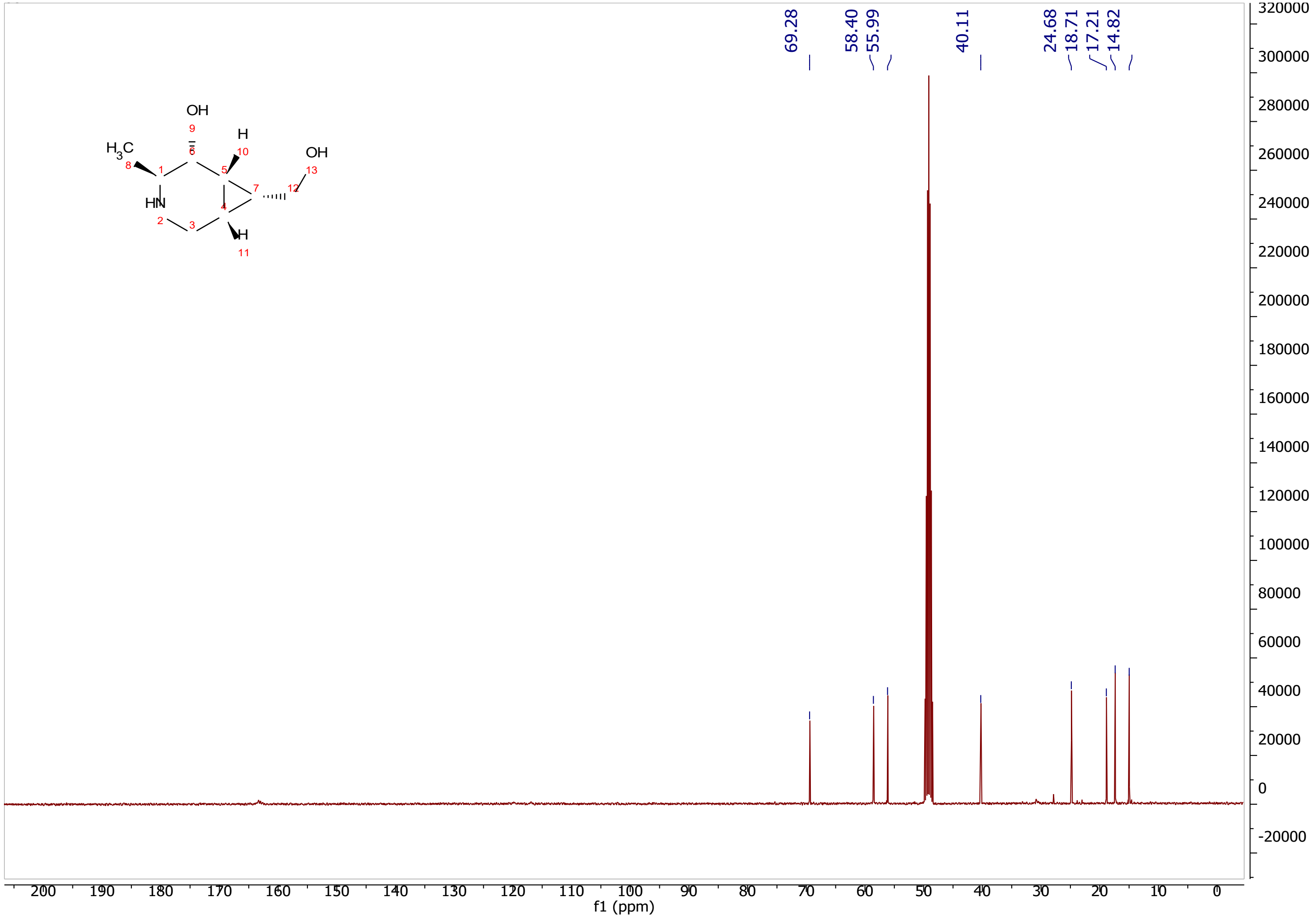

Figure S46: ^{1}H (400 MHz MeOD) bidimensional NOESY of **6c**.

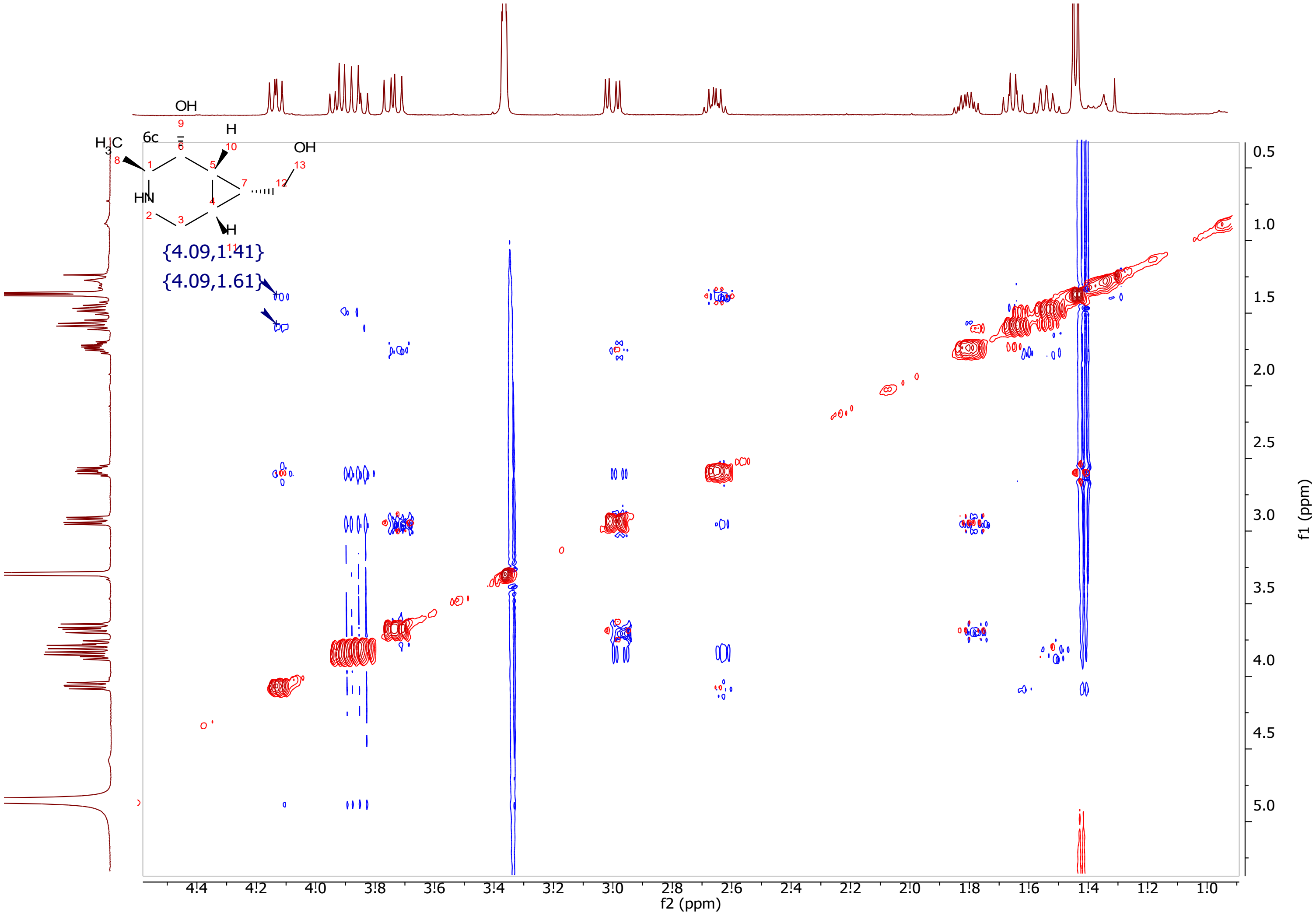

Figure S47: ^{1}H-NMR (400 MHz, $CDCl_3$) of **7**.

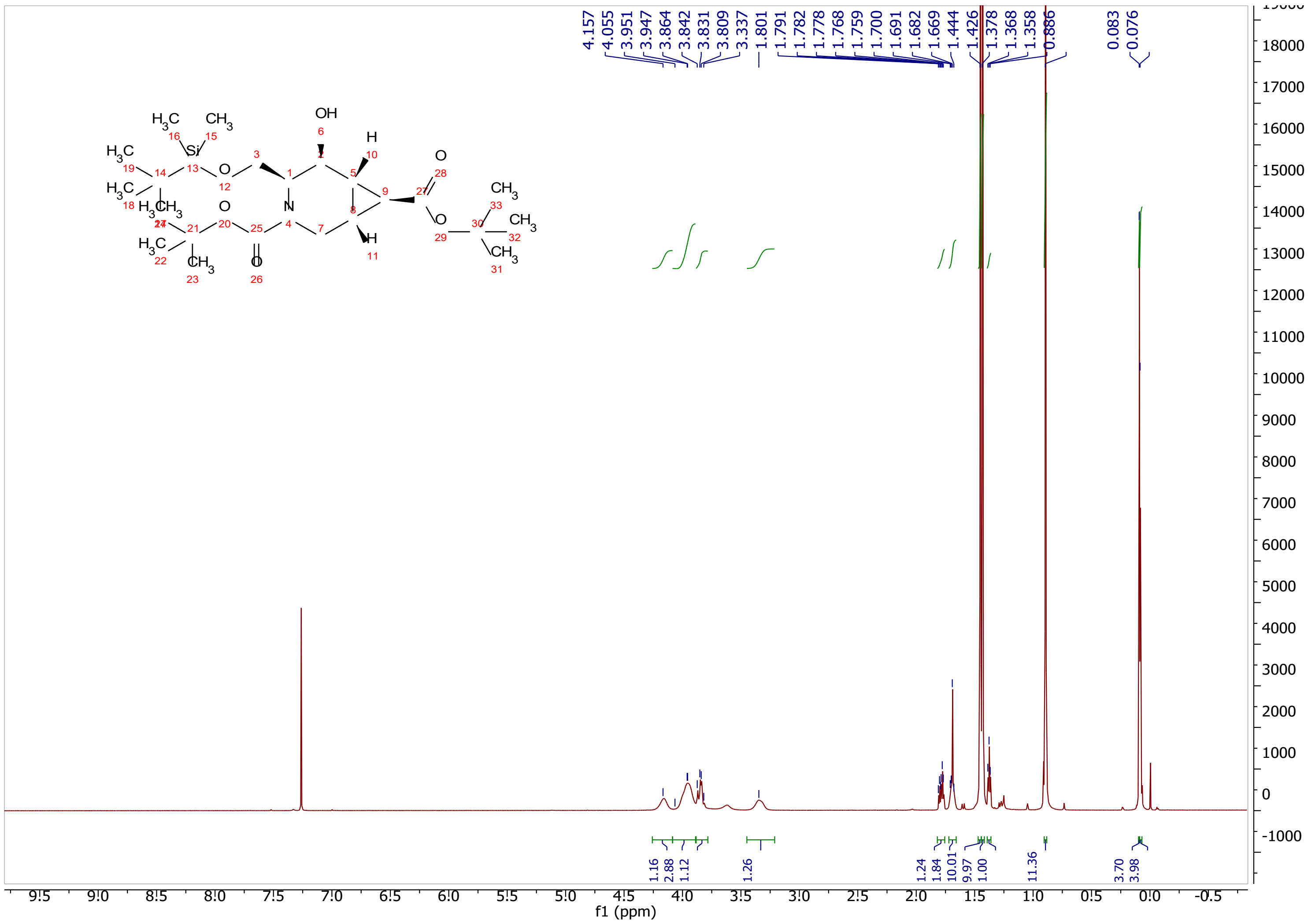

Figure S48: ^{13}C-NMR (100 MHz, $CDCl_3$) of **7**.

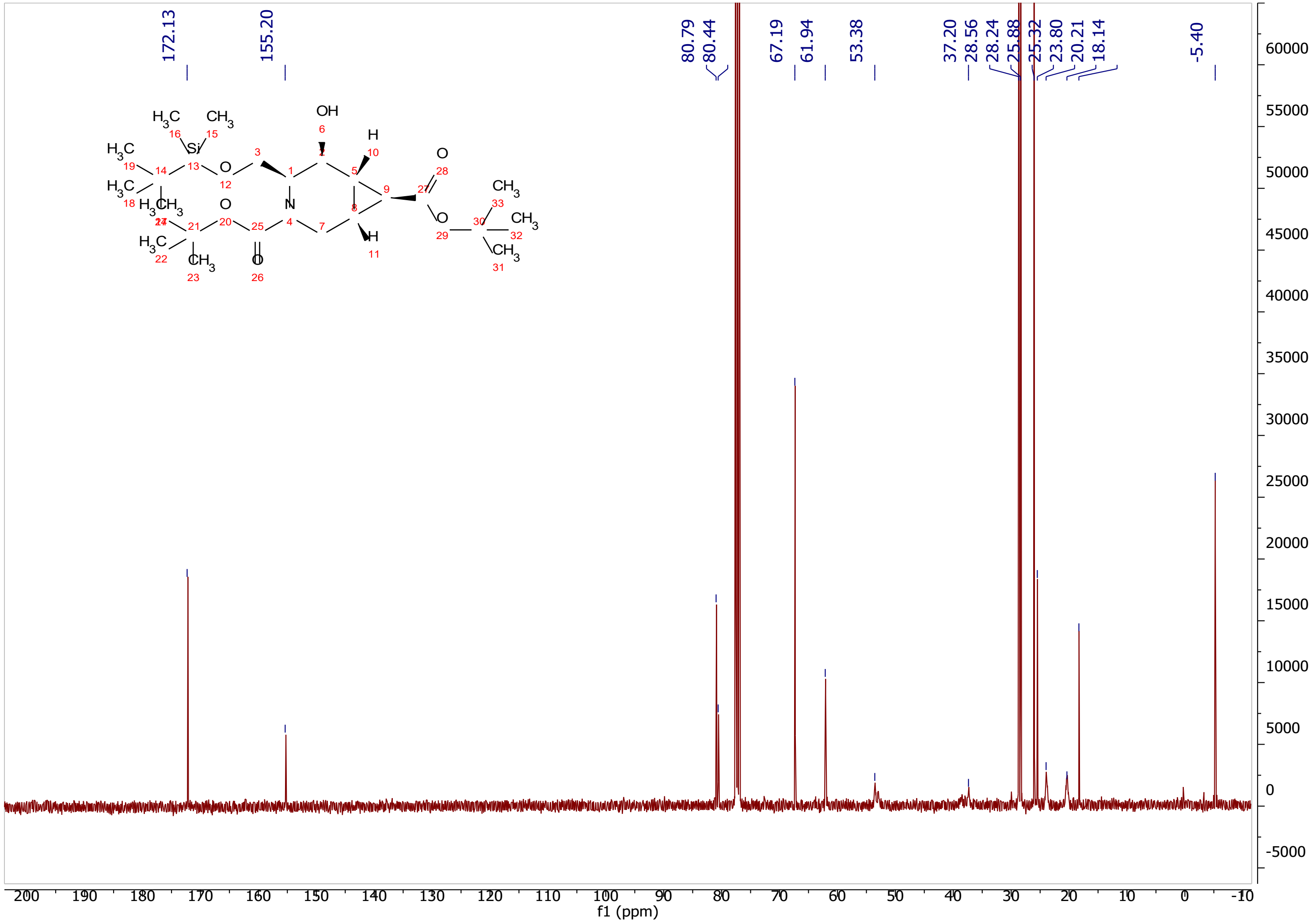

Figure S49: ¹H-NMR (400 MHz, MeOD) of **di-*tert*-butyl (1*R*,4*S*,5*S*,6*S*,7*S*)-5-hydroxy-4-(hydroxymethyl)-3-azabicyclo[4.1.0]heptane-3,7- dicarboxylate**.

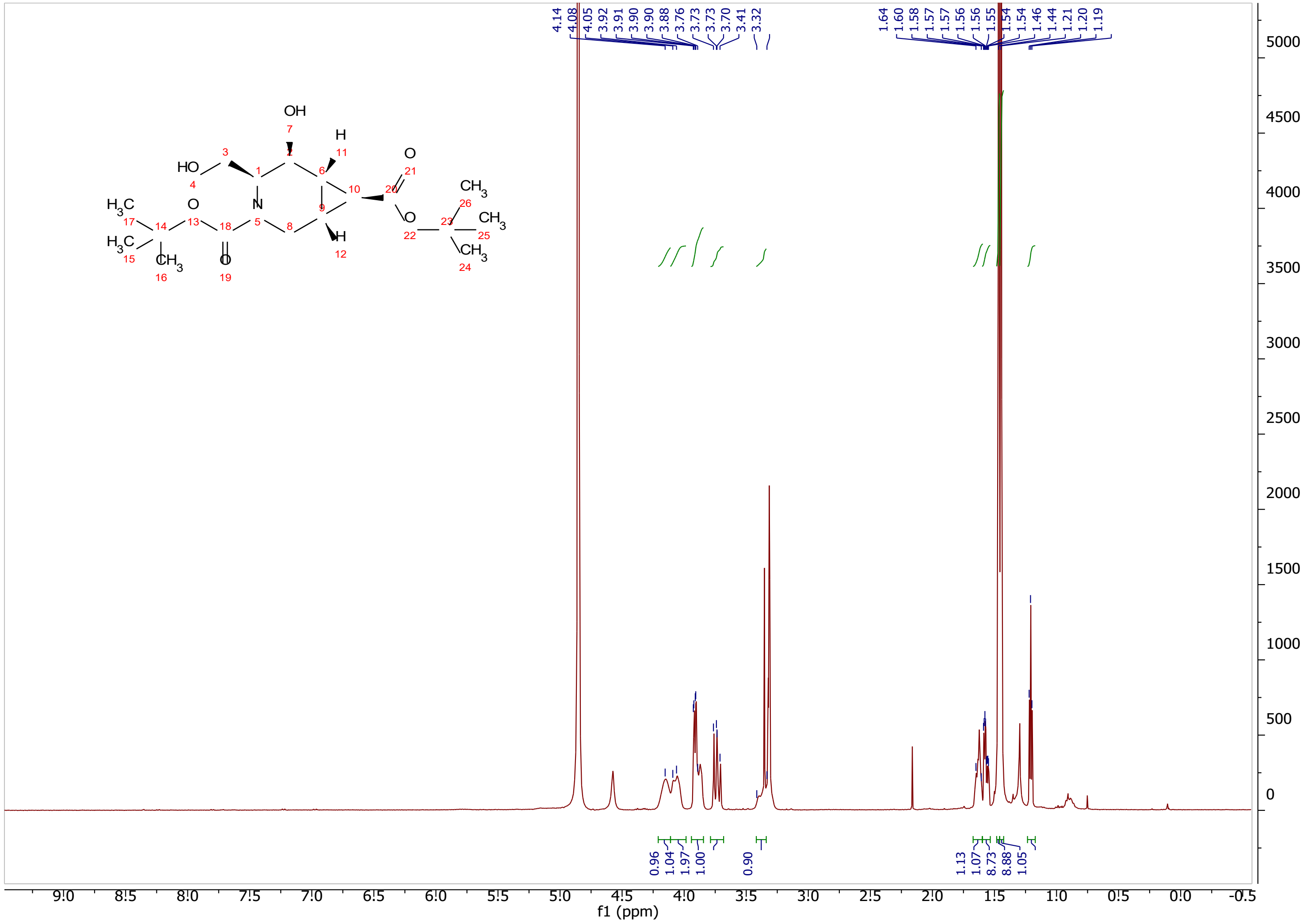

Figure S50: ¹³C-NMR (100 MHz, MeOD) of **di-*tert*-butyl (1R,4S,5S,6S,7S)-5-hydroxy-4-(hydroxymethyl)-3-azabicyclo[4.1.0]heptane-3,7- dicarboxylate.**

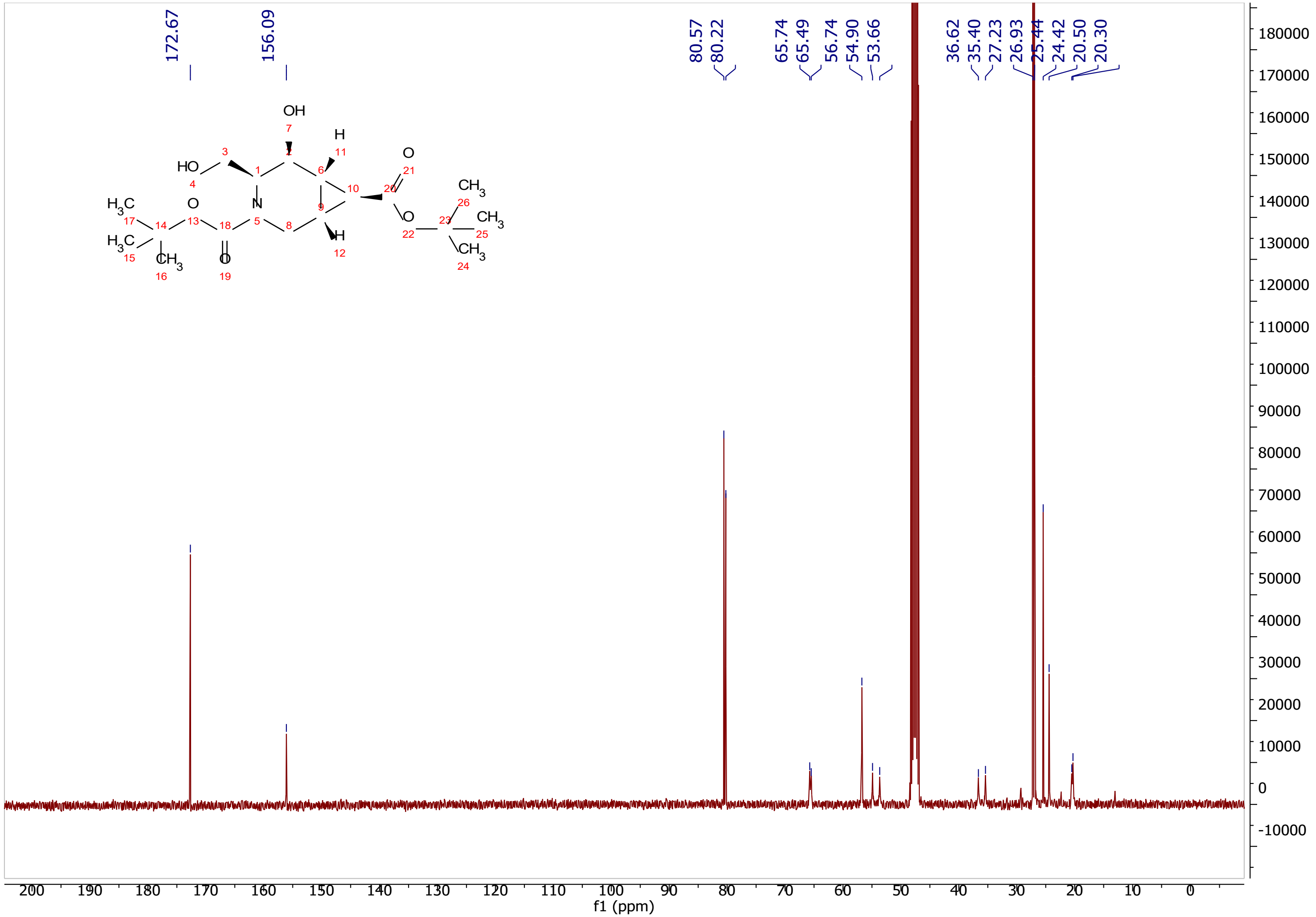

Figure S51: ¹H (400 MHz CDCl3) bidimensional NOESY of **di-*tert*-butyl (1*R*,4*S*,5*S*,6*S*,7*S*)-5-hydroxy-4-(hydroxymethyl)-3-azabicyclo[4.1.0]heptane-3,7-dicarboxylate.**

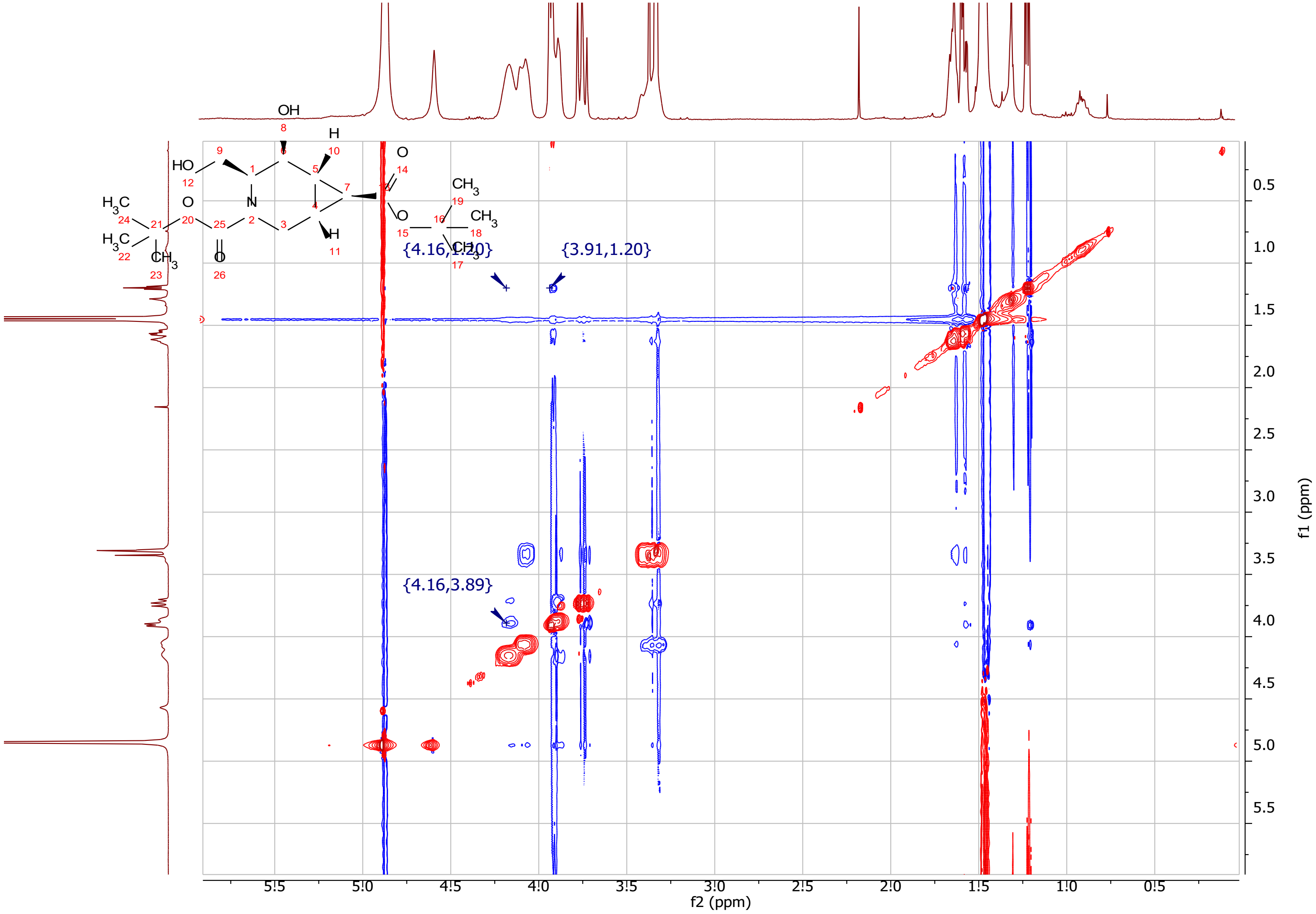

Figure S52: ¹H-NMR (400 MHz, D_2O) of **10a**.

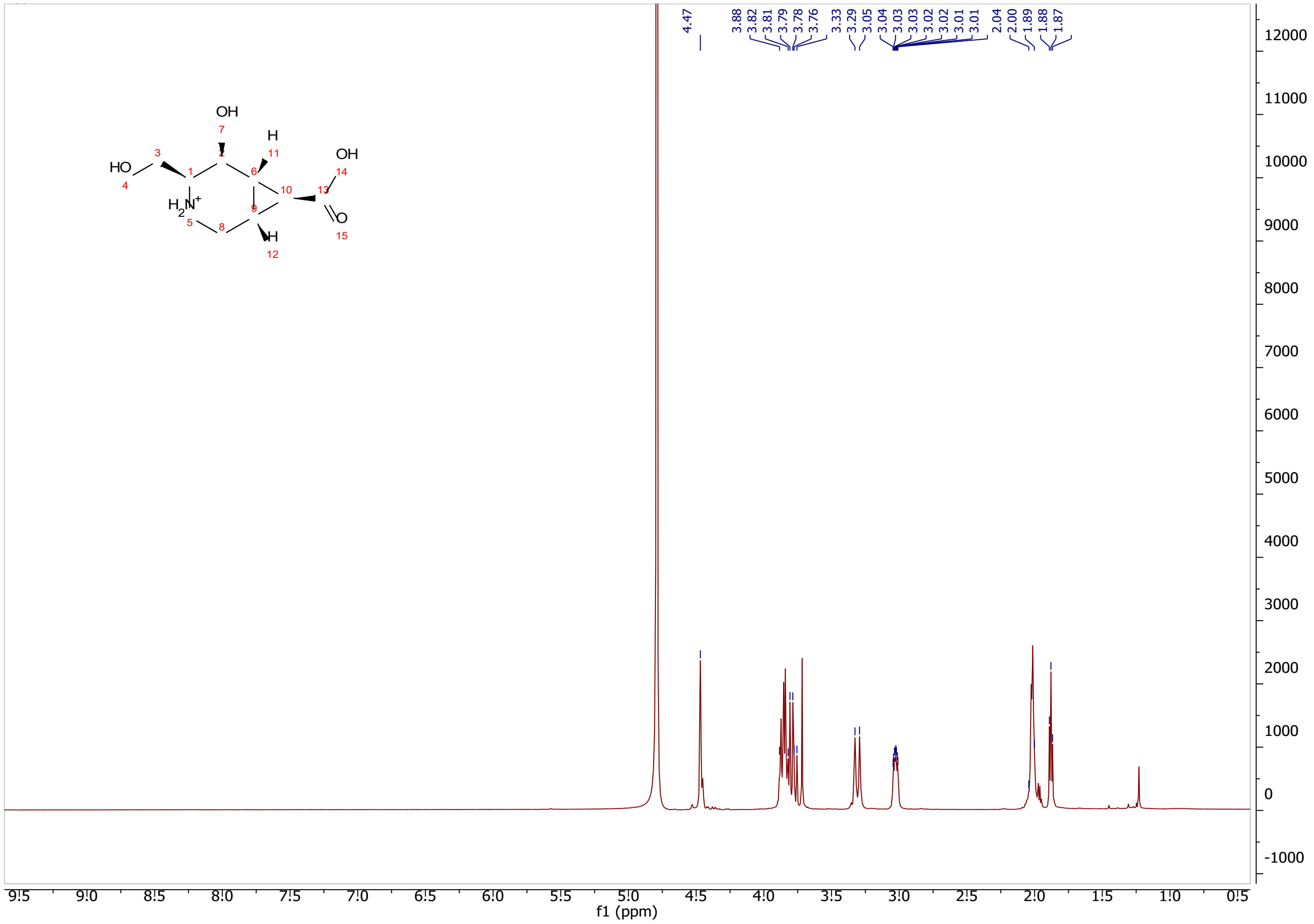

Figure S53: ^{13}C-NMR (100 MHz, D_2O) of **10a.**

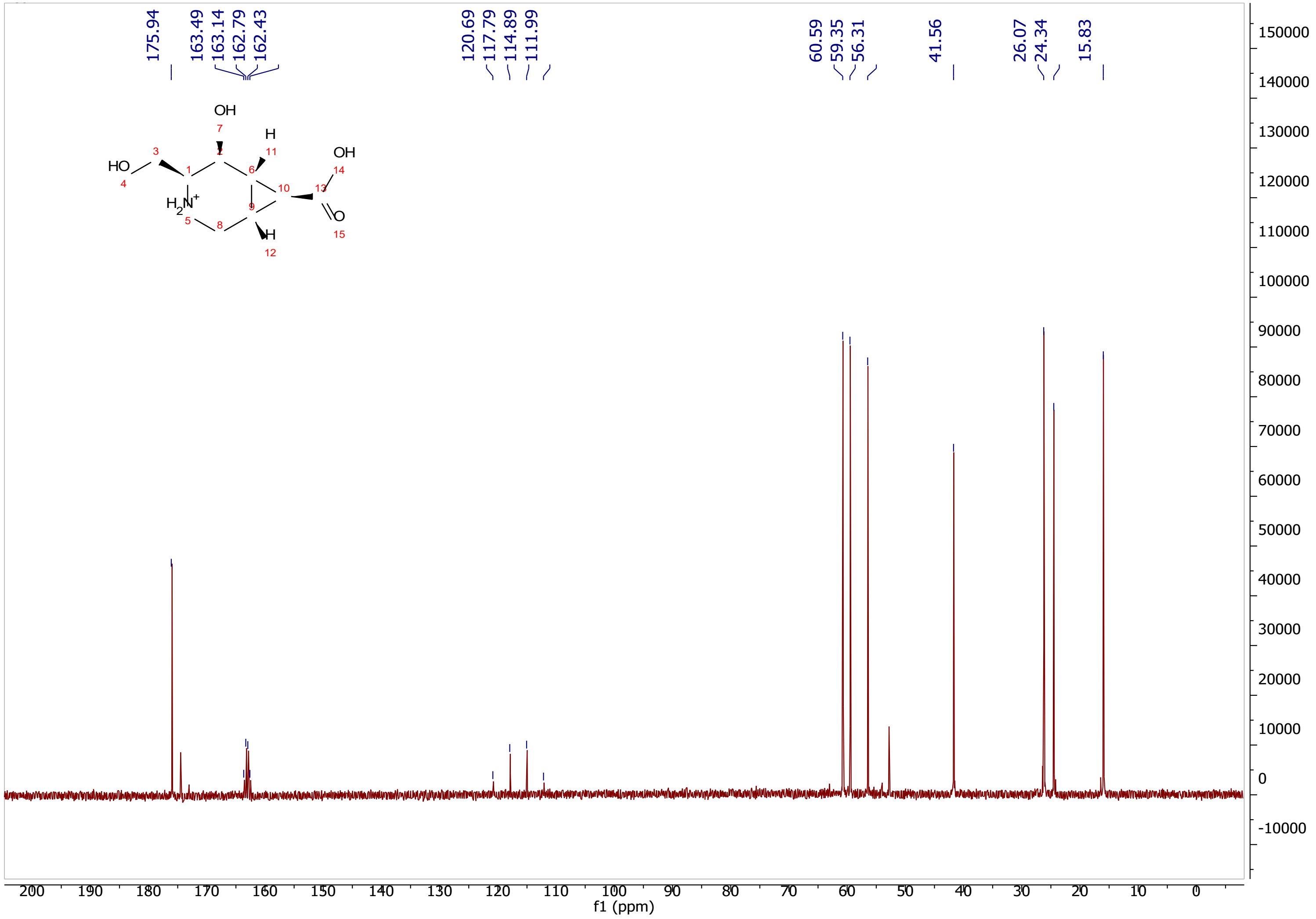

Figure S54: ¹H-NMR (400 MHz, $CDCl_3$) of ***tert*-butyl (1*S*,4*S*,5*S*,6*S*,7*S*)-4-(((*tert*-butyldimethylsilyl)oxy)methyl)-5-hydroxy-7- (hydroxymethyl)-3-azabicyclo[4.1.0]heptane-3-carboxylate.**

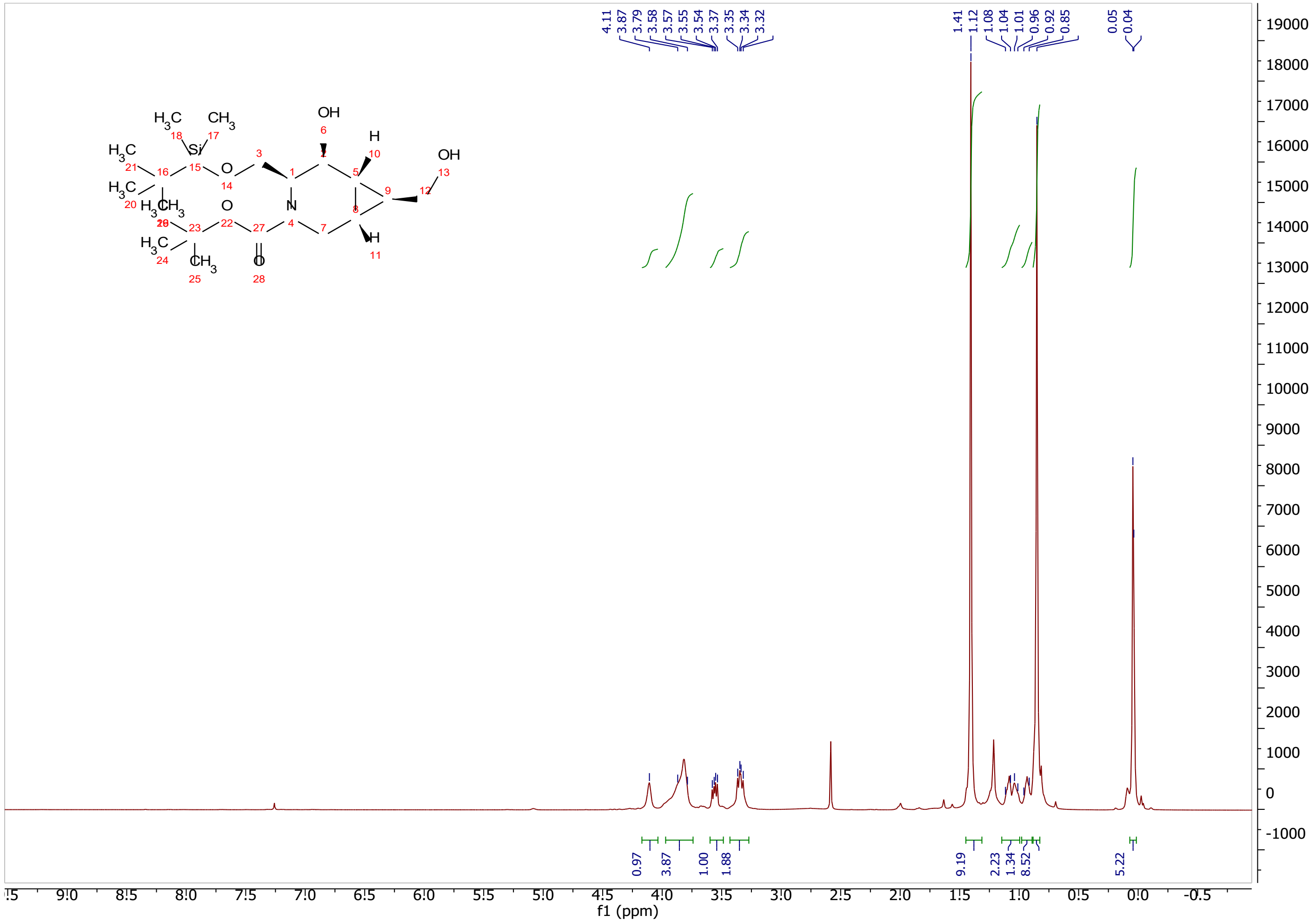

Figure S55: ^{13}C-NMR (100 MHz, MeOD) of ***tert*-butyl (1*S*,4*S*,5*S*,6*S*,7*S*)-4-(((*tert*-butyldimethylsilyl)oxy)methyl)-5-hydroxy-7- (hydroxymethyl)-3-azabicyclo[4.1.0]heptane-3-carboxylate**.

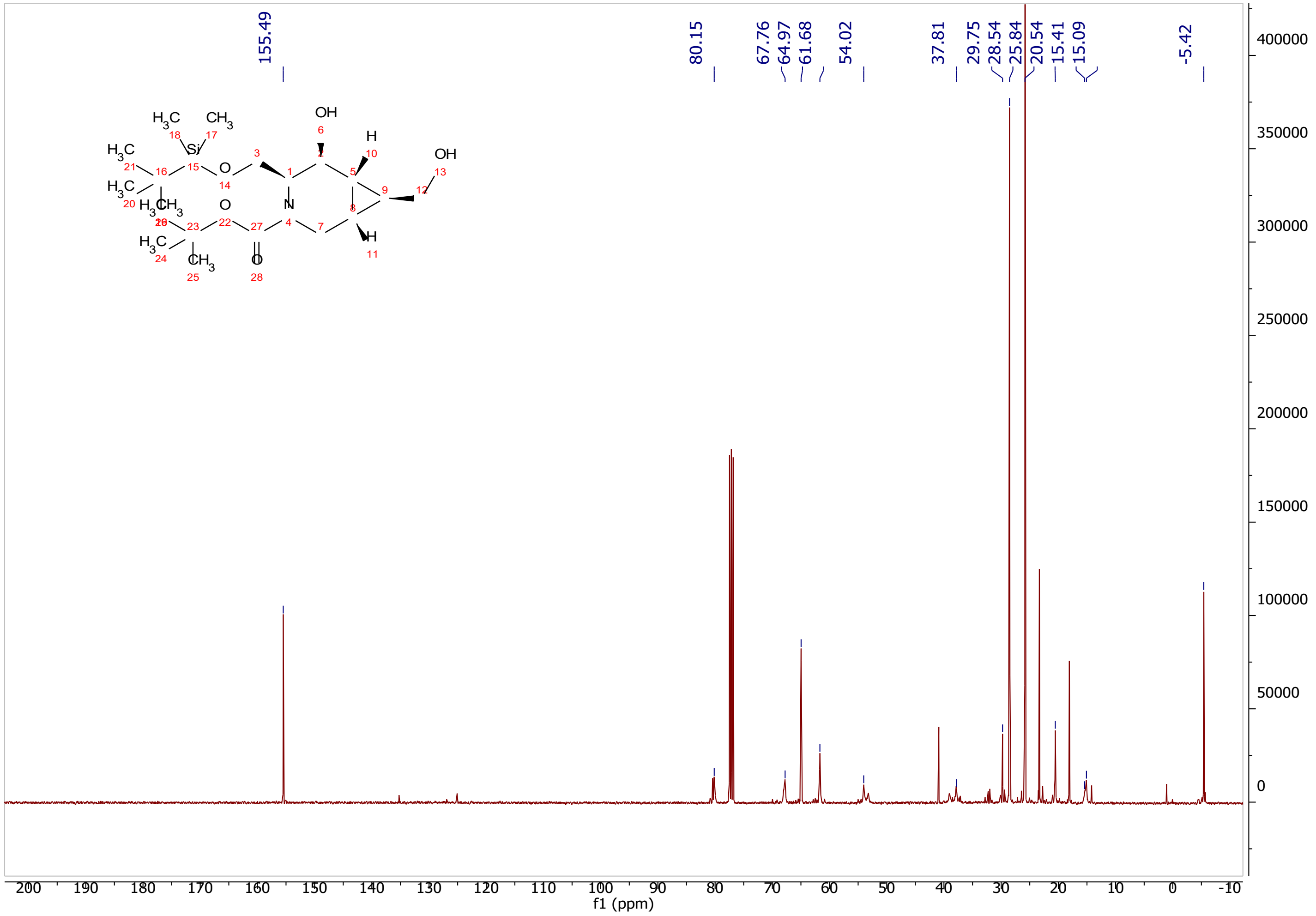

Figure S56: ^{1}H-NMR (400 MHz, MeOD) of ***tert*-butyl (1*S*,4*S*,5*S*,6*S*,7*S*)-5-hydroxy-4,7-bis(hydroxymethyl)-3-azabicyclo[4.1.0]heptane-3- carboxylate.**

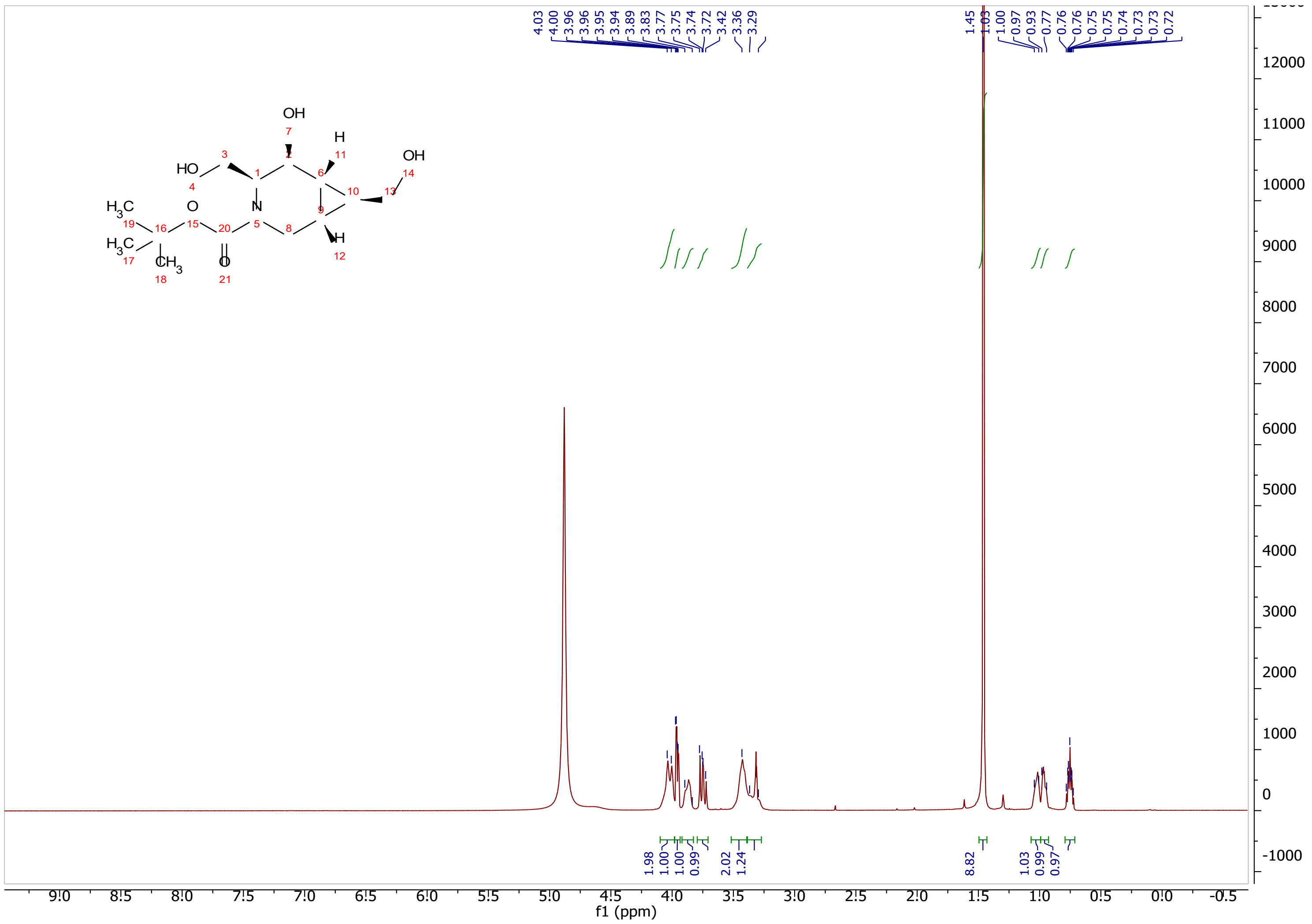

Figure S57: ^{13}C-NMR (100 MHz, MeOD) of ***tert*-butyl (1*S*,4*S*,5*S*,6*S*,7*S*)-5-hydroxy-4,7-bis(hydroxymethyl)-3-azabicyclo[4.1.0]heptane-3- carboxylate**.

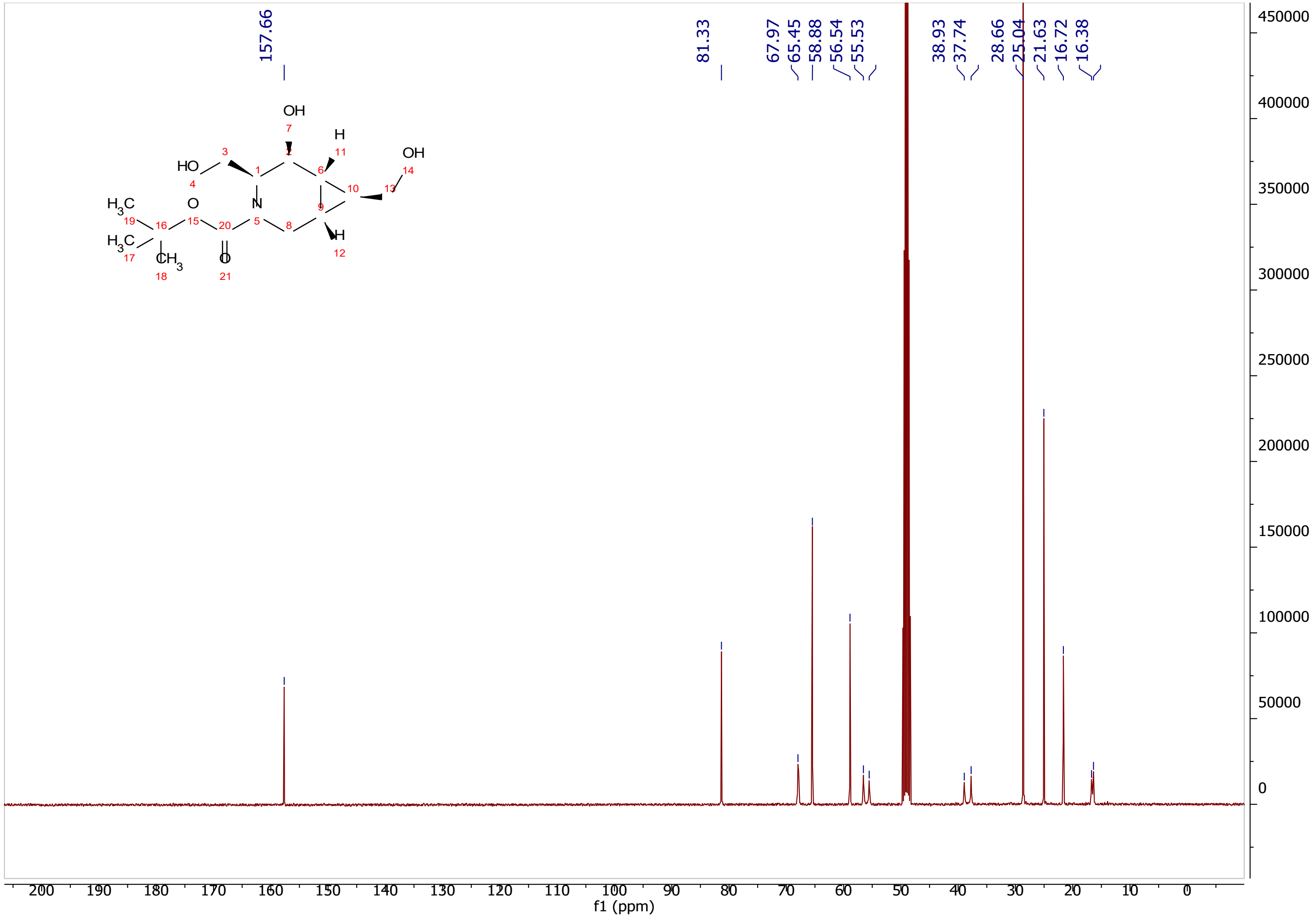

Figure S58: ¹H-NMR (400 MHz, MeOD) of **11a**.

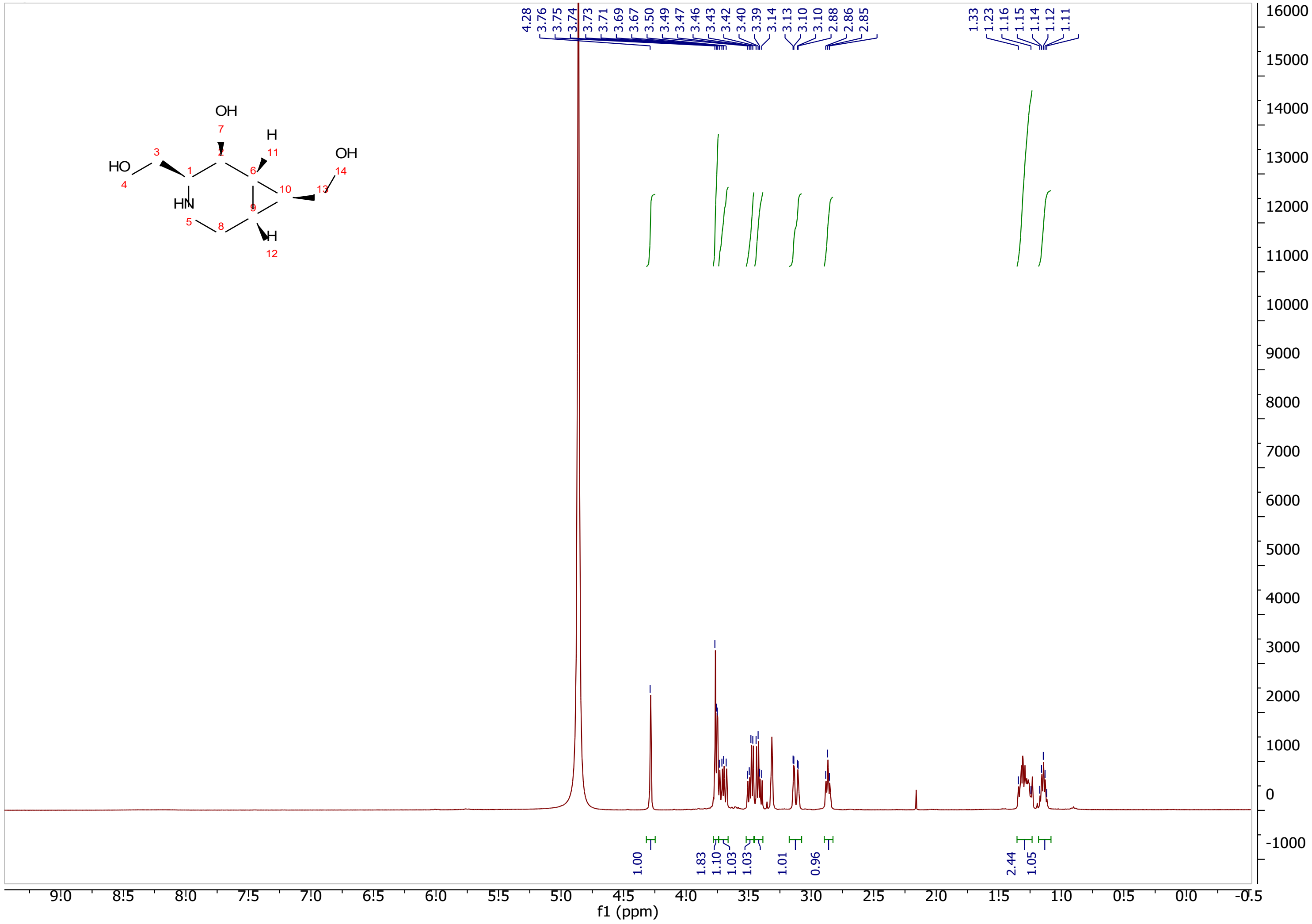

Figure S59: ^{13}C-NMR (100 MHz, MeOD) of **11a**.

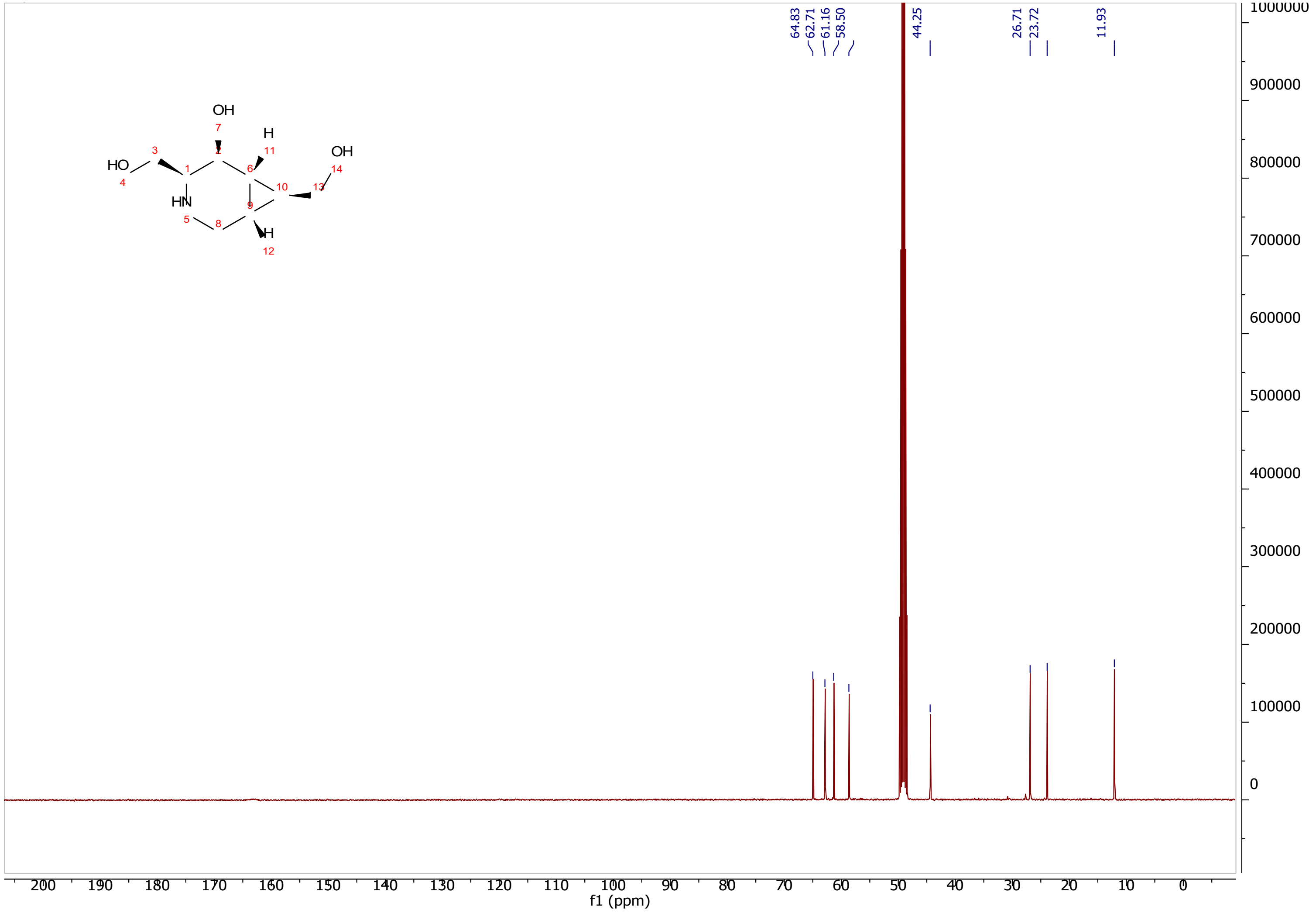

Figure S60: ¹H-NMR (400 MHz, $CDCl_3$) of **8**.

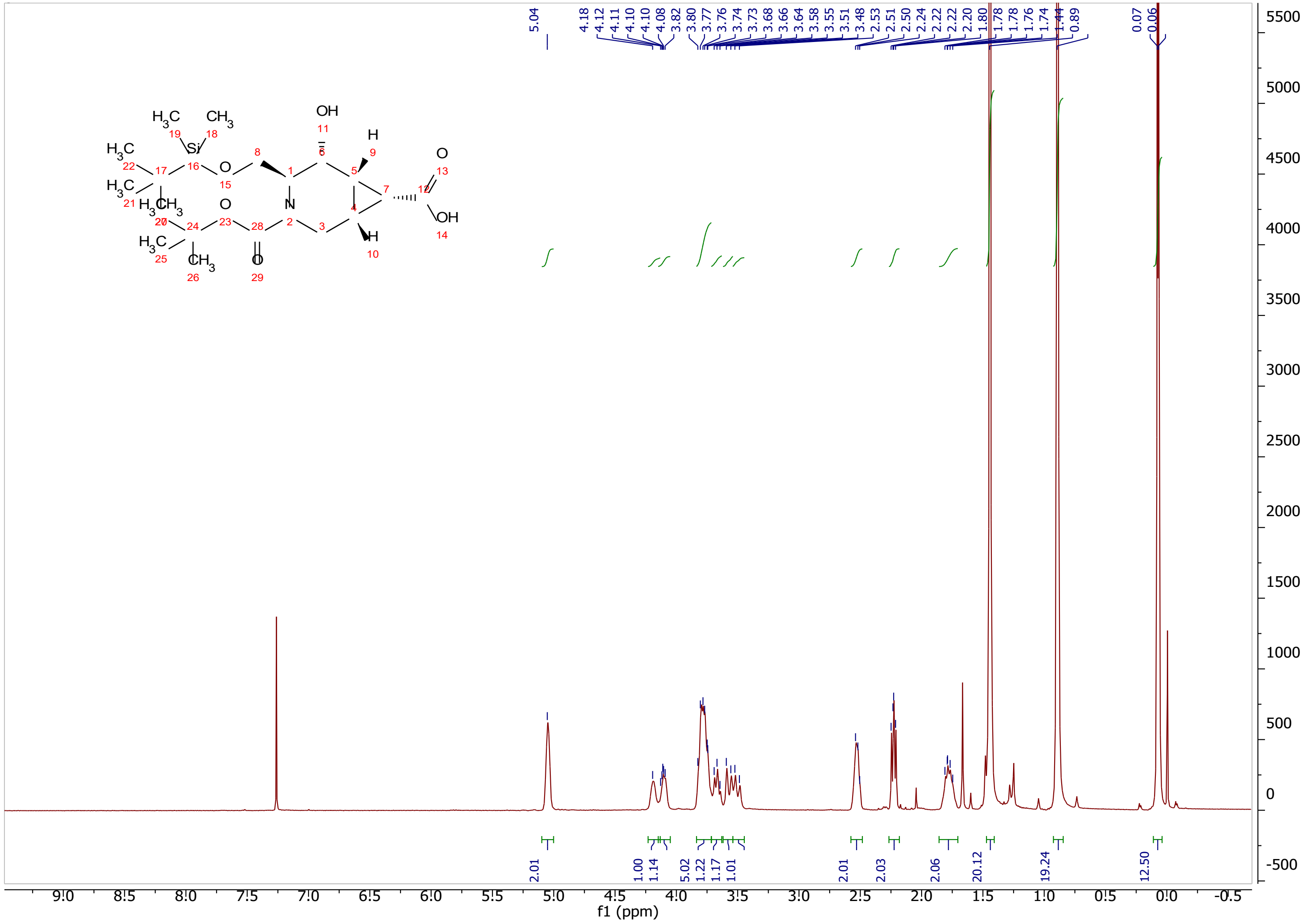

Figure S61: ^{13}C-NMR (100 MHz, $CDCl_3$) of **8**.

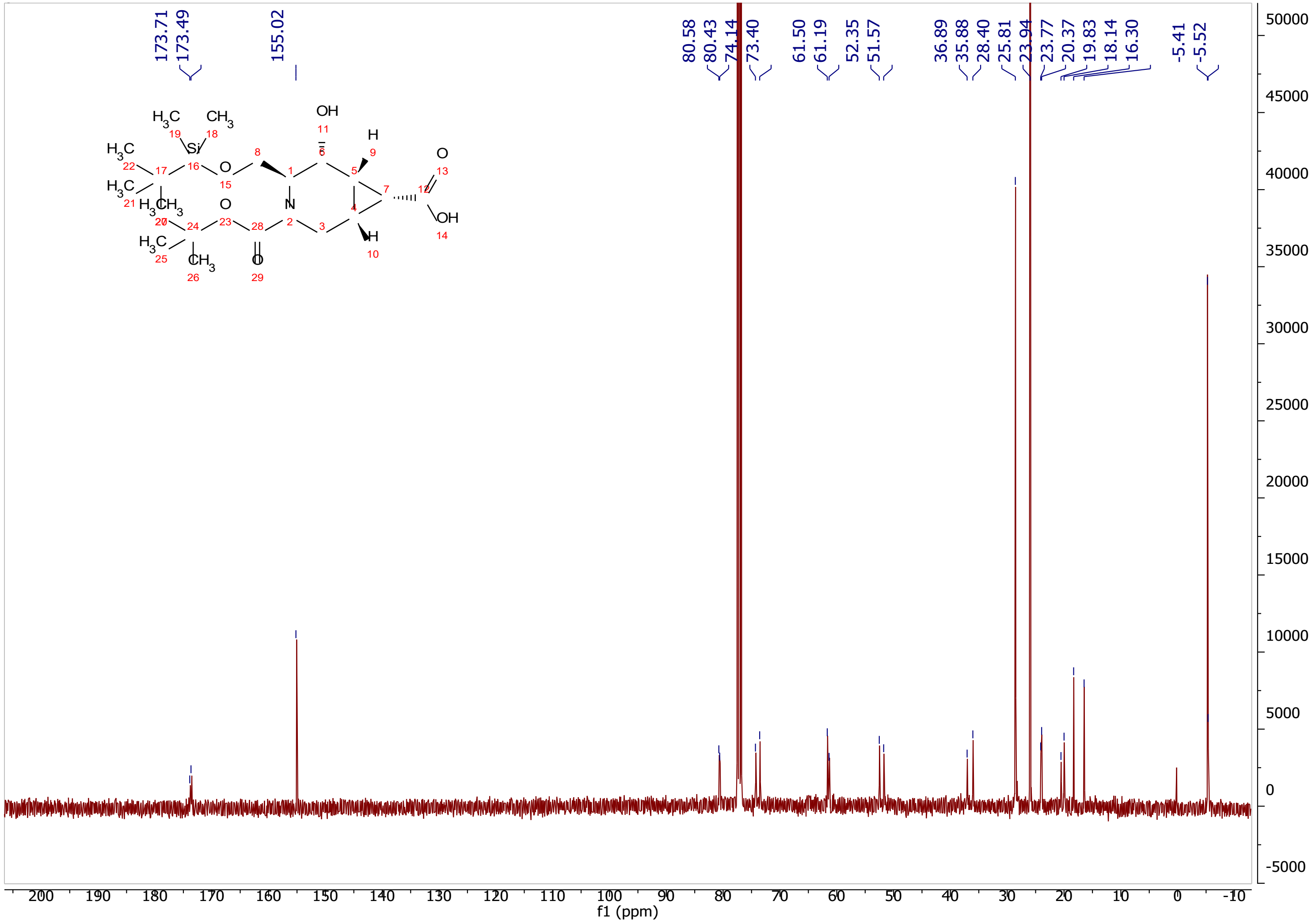

Figure S62: ^{1}H-NMR (400 MHz, $CDCl_3$) of **(1*R*,4*S*,5*S*,6*S*,7*R*)-3-(*tert*-butoxycarbonyl)-5-hydroxy-4-(hydroxymethyl)-3-azabicyclo[4.1.0]heptane-7-carboxylic acid**.

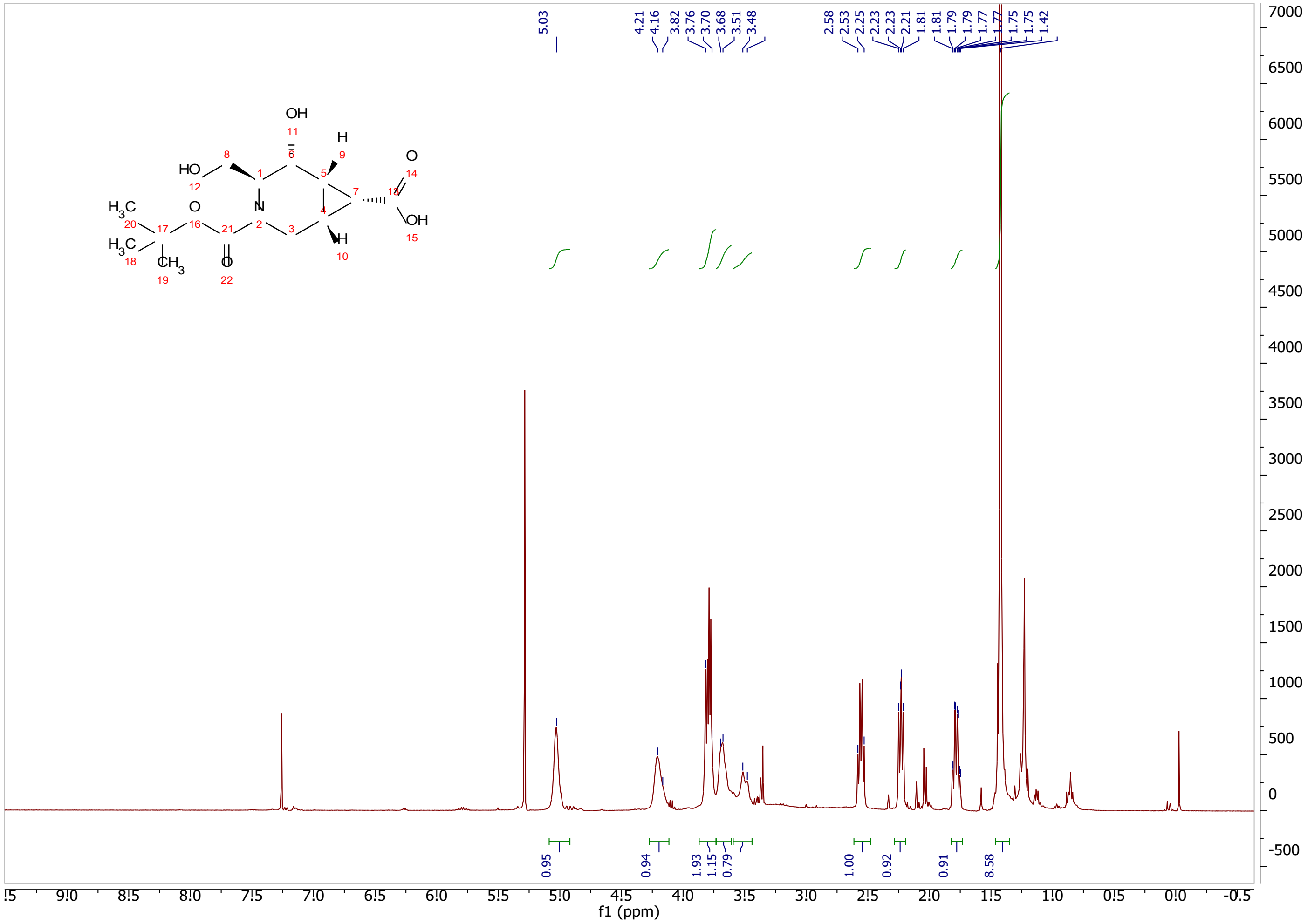

Figure S63: ^{13}C-NMR (100 MHz, $CDCl_3$) of **(1*R*,4*S*,5*S*,6*S*,7*R*)-3-(*tert*-butoxycarbonyl)-5-hydroxy-4-(hydroxymethyl)-3-azabicyclo[4.1.0]heptane-7-carboxylic acid**.

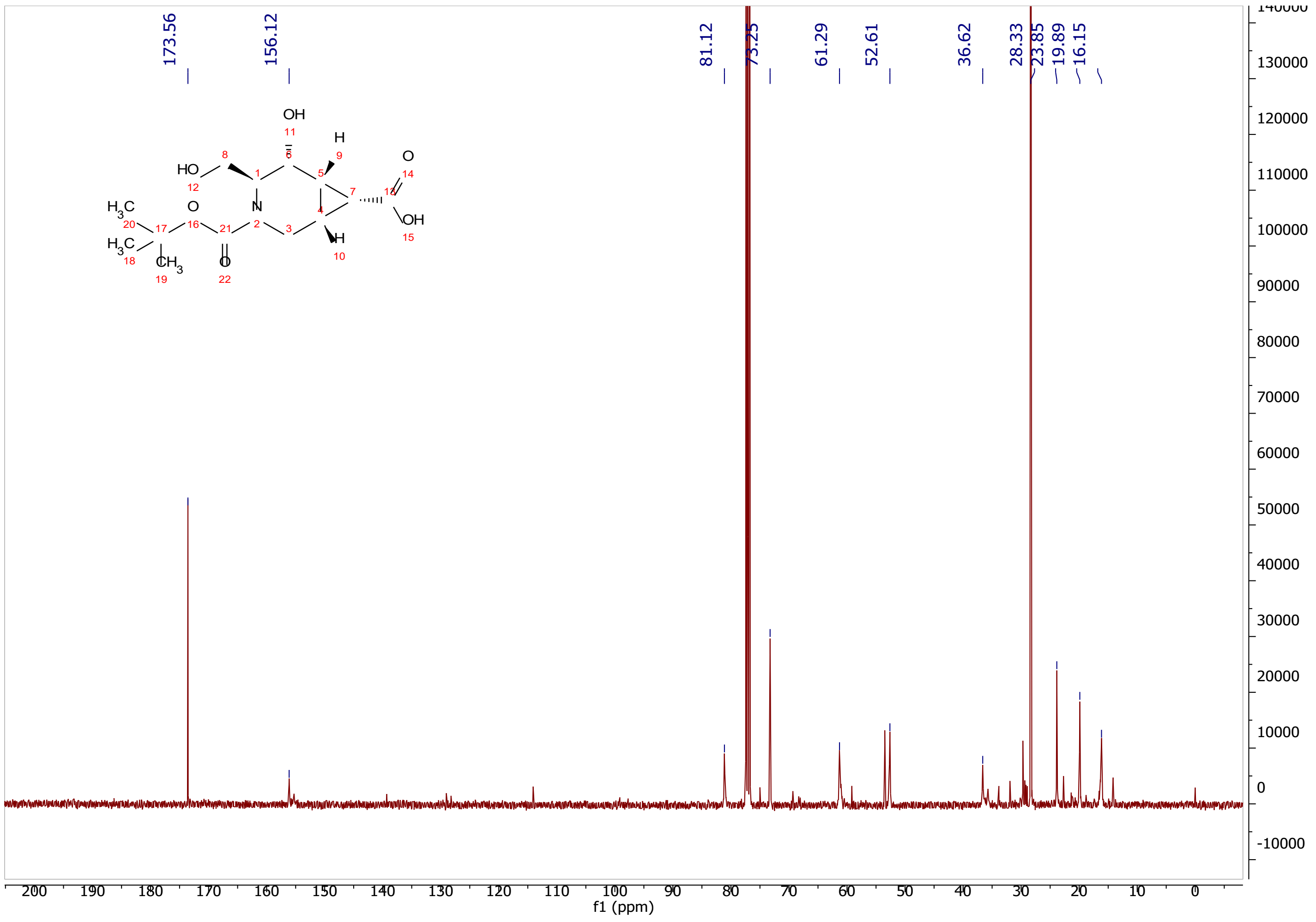

Figure S64: ^{1}H-NMR (400 MHz, D_2O) of **10b**.

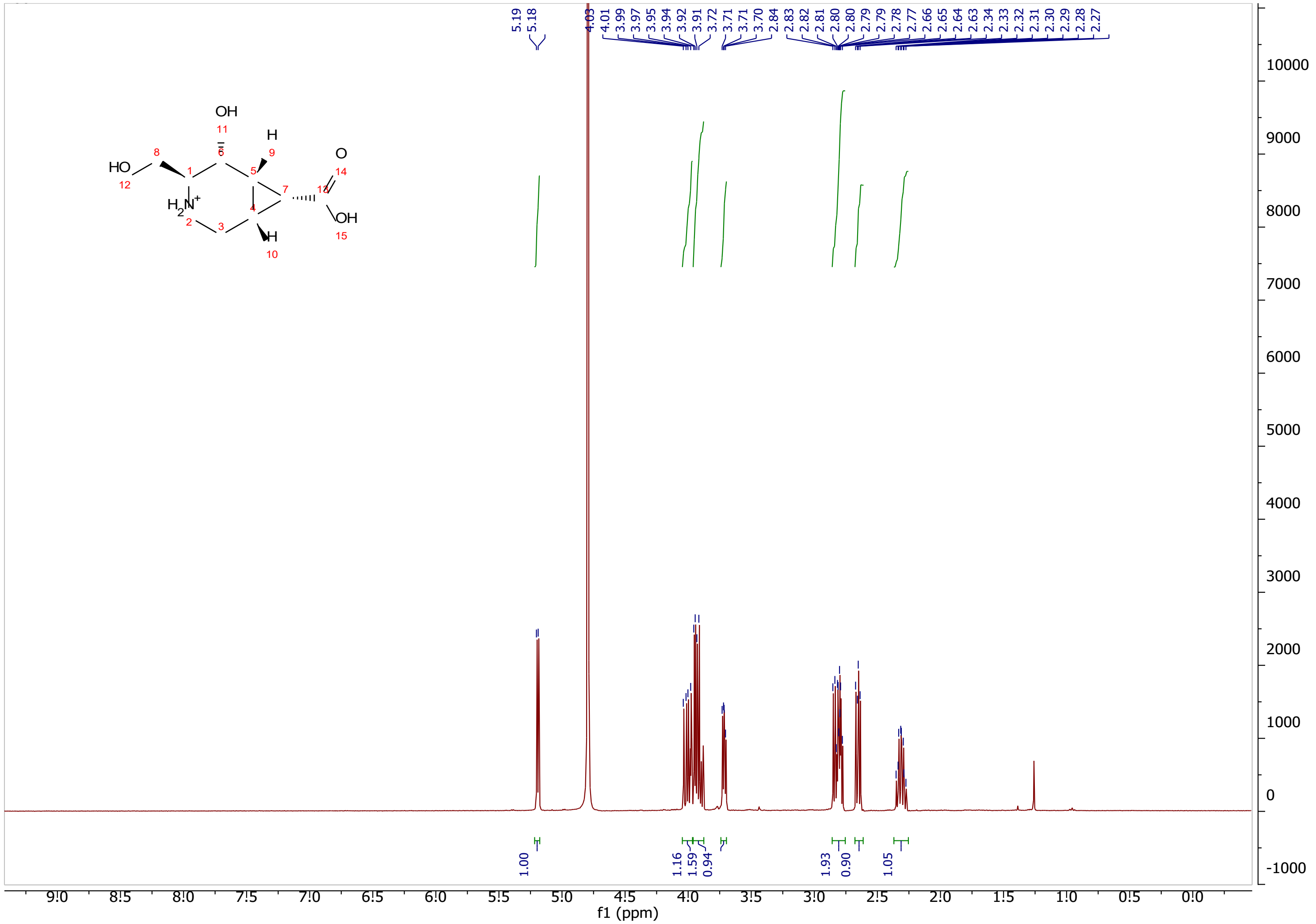

Figure S65: ^{13}C-NMR (100 MHz, D_2O) of **10b**.

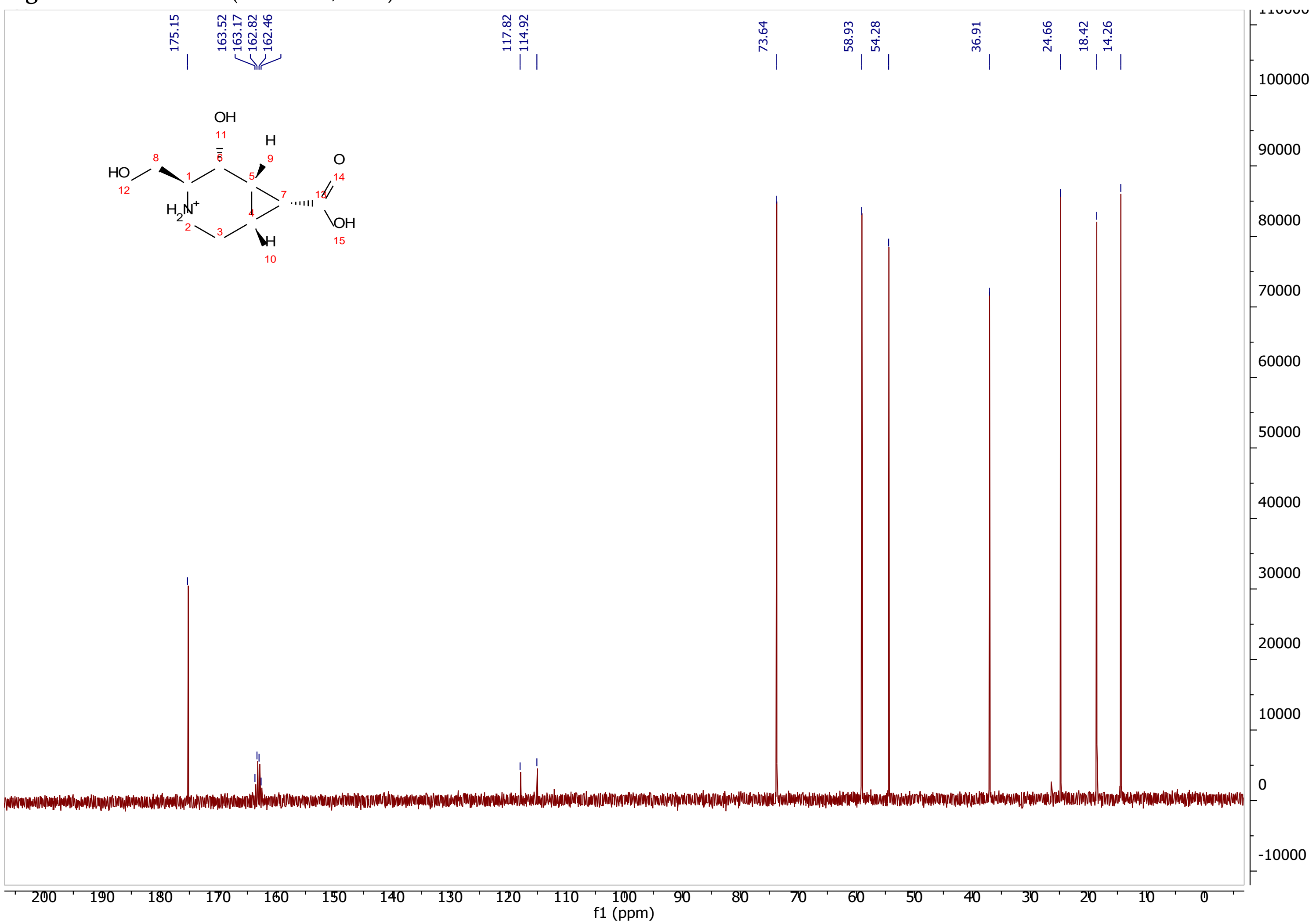

Figure S66: ^{1}H-NMR (400 MHz, $CDCl_3$) of **9**.

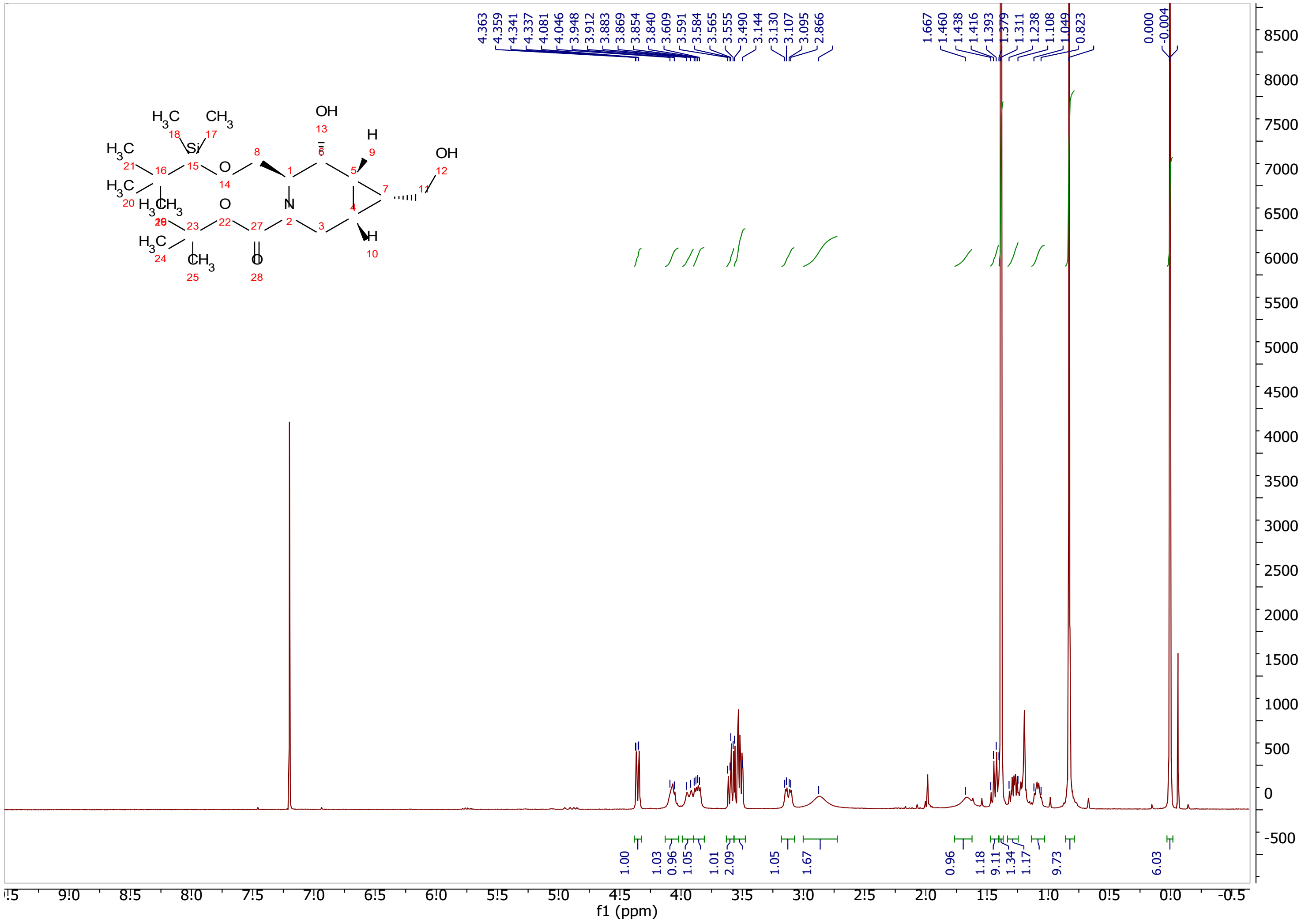

Figure S67: ^{13}C-NMR (100 MHz, $CDCl_3$) of **9**.

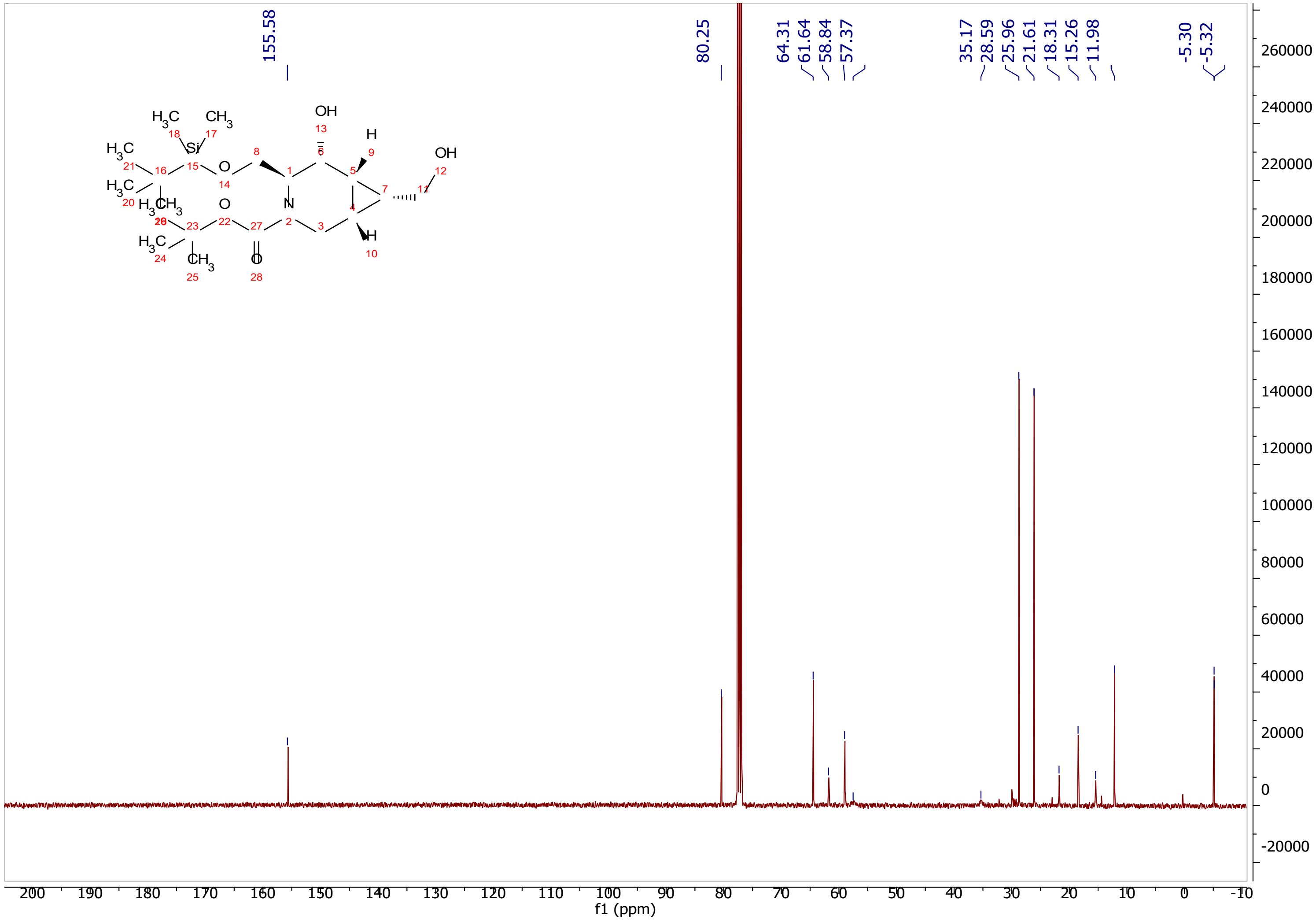

Figure S68: ^{1}H-NMR (400 MHz, MeOD) of ***tert*-butyl (1*S*,4*S*,5*S*,6*S*,7*R*)-5-hydroxy-4,7-bis(hydroxymethyl)-3-azabicyclo[4.1.0]heptane-3- carboxylate.**

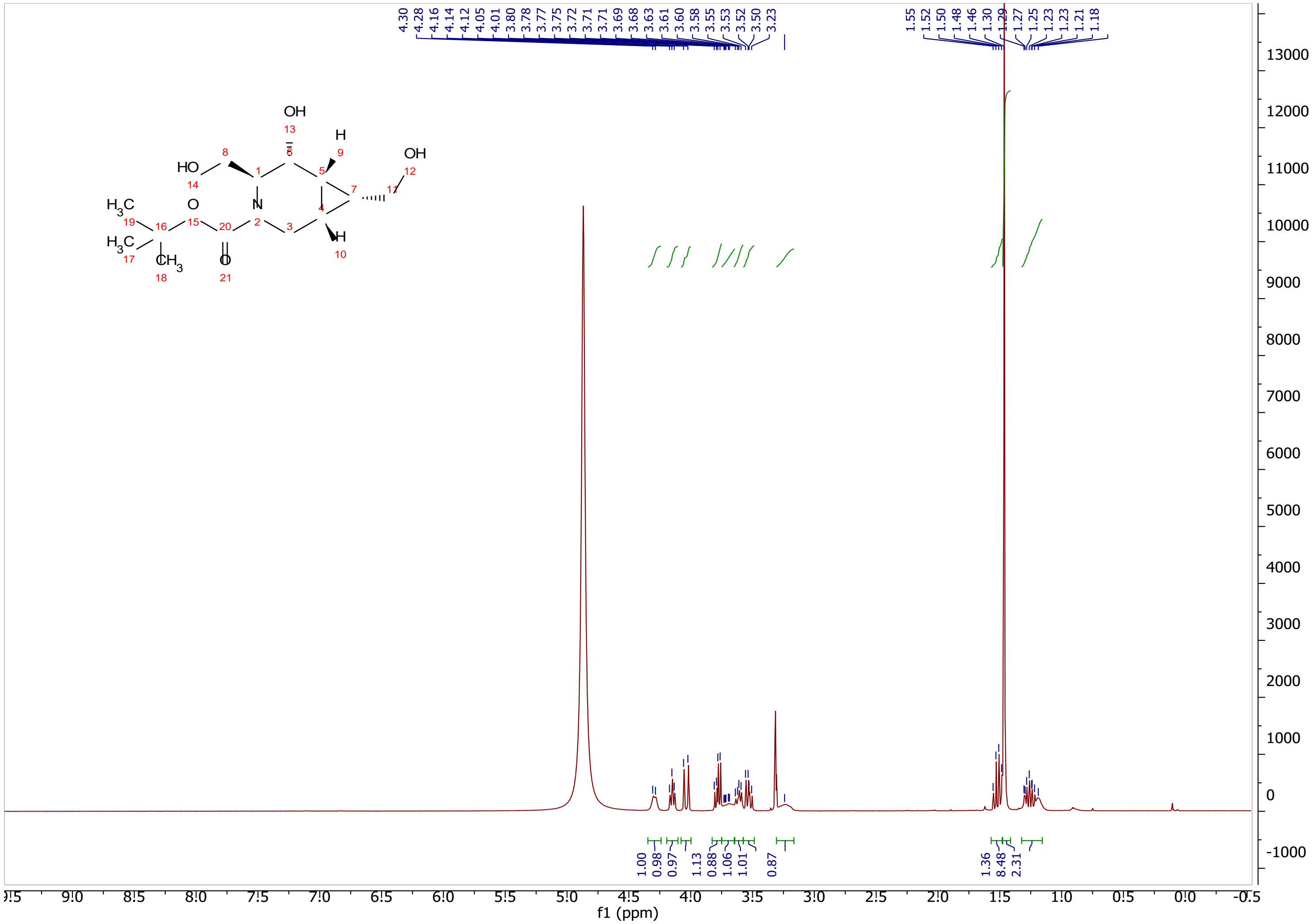

Figure S69: ^{13}C-NMR (100 MHz, MeOD) of ***tert*-butyl (1*S*,4*S*,5*S*,6*S*,7*R*)-5-hydroxy-4,7-bis(hydroxymethyl)-3-azabicyclo[4.1.0]heptane-3- carboxylate**.

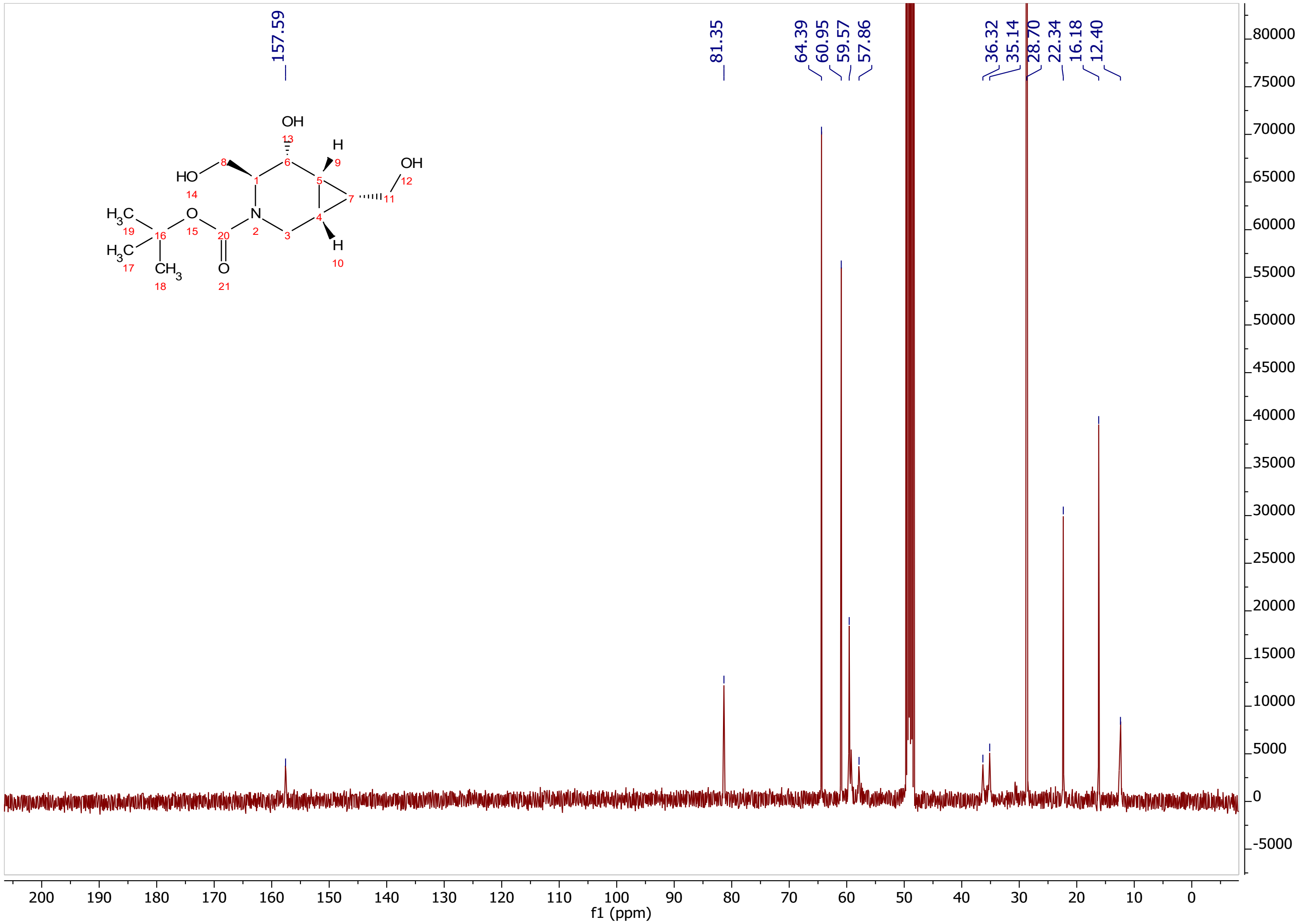

Figure S70: ¹H-NMR (400 MHz, MeOD) of **11b**.

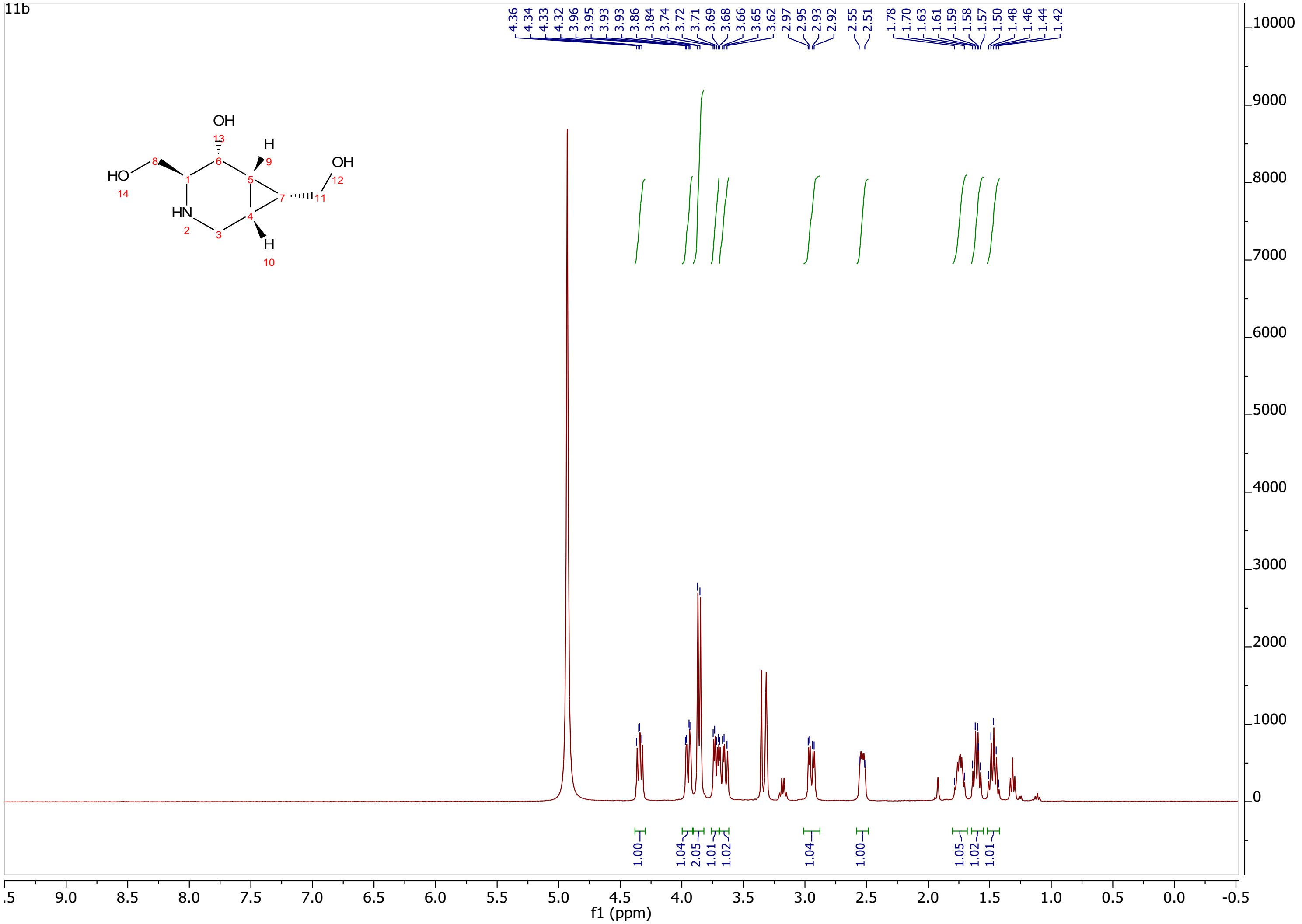

Figure S71: ^{13}C-NMR (100 MHz, MeOD) of **11b**.

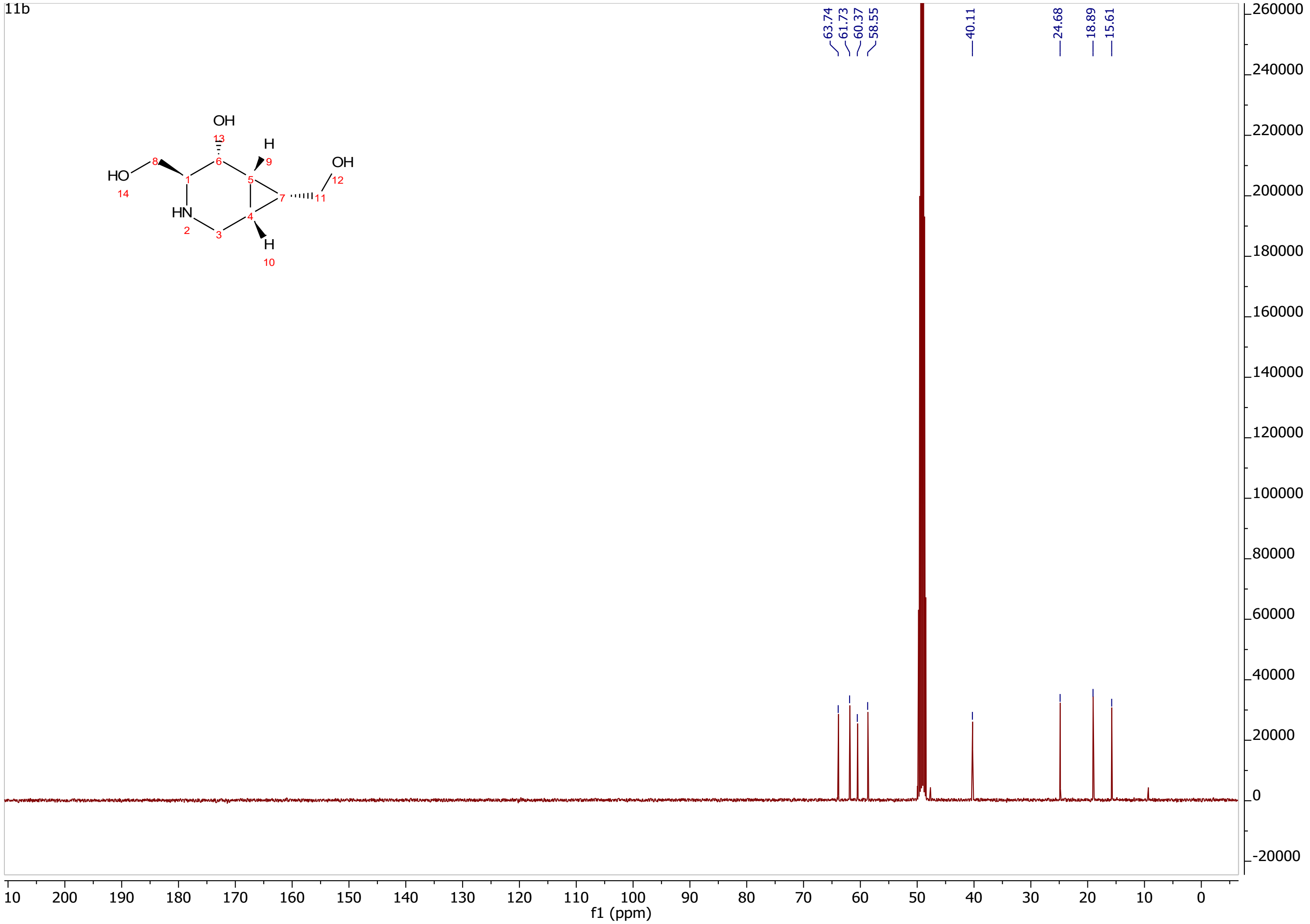

Figure S72: ^{1}H (400 MHz MeOD) bidimensional NOESY of **11b**.

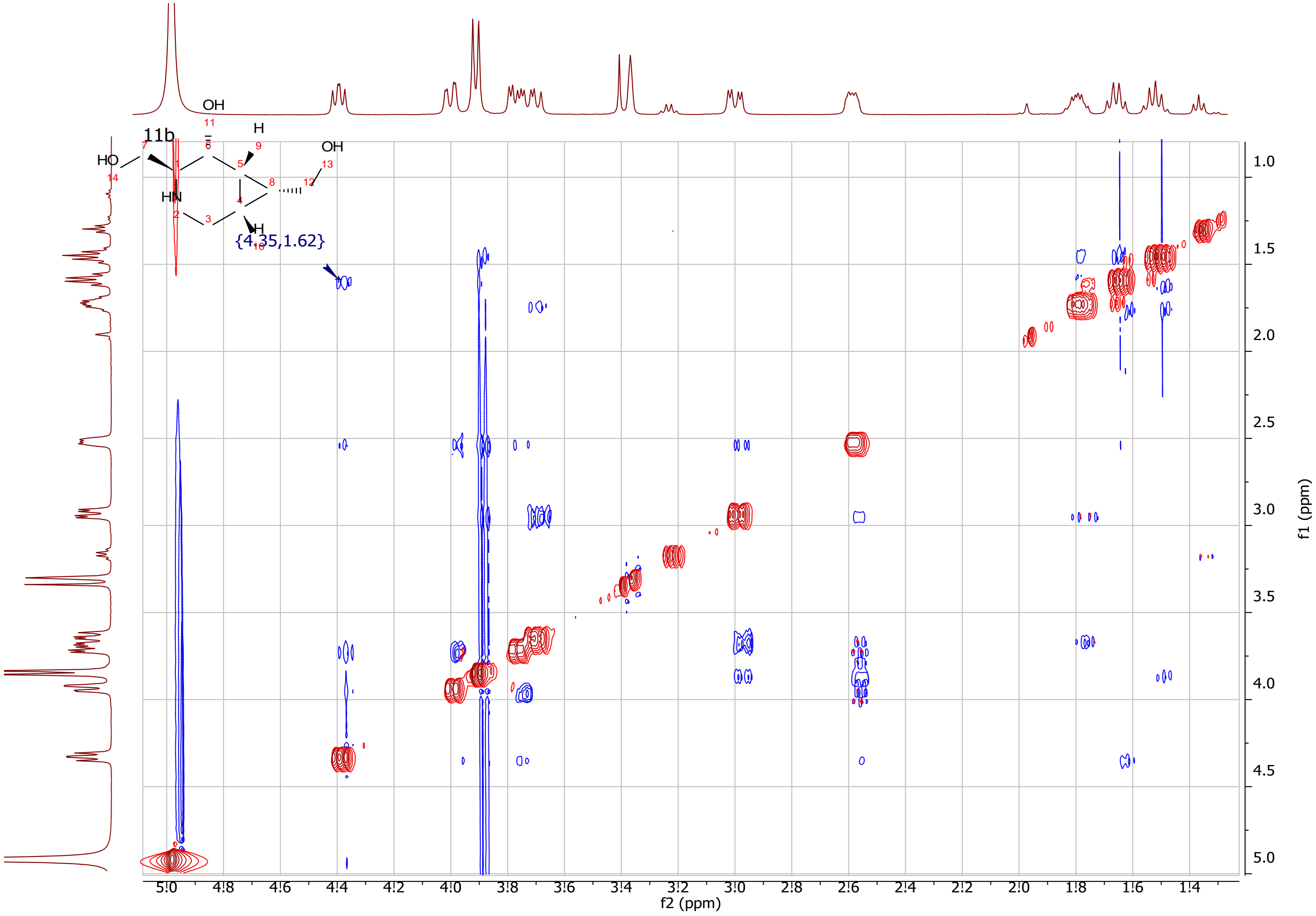

Table S1: Measured constant coupling from products derived from L-serine.

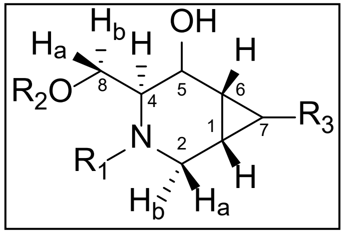

	3b	4b	7	10a	11a	8	28	10b	9	25b	11b
J_{8a-8b}	10.0	10.4		12.2				10.7	11.5	7.6	11.9
J_{8a-4}	2.9	2.7		8.5	6.4			5.4	5.7	7.6	5.1
J_{8b-4}	2.9	2.7		1.5	1.7			5.4	5.7	7.3	3.0
J_{4-5}				4.4	5.5			0	1.4		10.1
J_{5-6}			1.8					5.5	8.8	8.9	7.6
J_{6-7}	4.4	8.6	4.1	4.6	5.6	6.2	6.2	5.9	8.9	9.1	8.9
J_{6-1}		8.6	9.3				8.0	8.3	8.9	9.1	8.9
J_{7-1}	4.4	8.6	4.1	4.6	5.6	8.5	8.0	8.4			8.4
J_{1-2a}	1.9	3.2			8.3		8.0	8.7	5.3		9.6
J_{1-2b}	0	0		0	2.4		2.5	6.4	0	0	5.0
J_{2a-2b}	13.5	13.4		13.5	13.7		14.5	14.7	14.2	14.1	14
